# Increasing crop field size does not consistently exacerbate insect pest problems

**DOI:** 10.1073/pnas.2208813119

**Published:** 2022-09-06

**Authors:** Jay A. Rosenheim, Emma Cluff, Mia K. Lippey, Bodil N. Cass, Daniel Paredes, Soroush Parsa, Daniel S. Karp, Rebecca Chaplin-Kramer

**Affiliations:** ^a^Department of Entomology and Nematology, University of California, Davis, CA 95616;; ^b^Environmental Resources Analysis Research Group, Department of Plant Biology, Ecology and Earth Sciences, Universidad de Extremadura, Badajoz 06006, Spain;; ^c^Regional Office for Latin America and the Caribbean, Food and Agriculture Organization of the United Nations, Avenida Dag Hammarskjöld 3241, Vitacura Santiago 7630000, Chile;; ^d^Department of Wildlife, Fish, and Conservation Biology, University of California, Davis, CA 95616;; ^e^Institute on the Environment, University of Minnesota, St. Paul, MN 55108;; ^f^SPRING, Oakland, CA 94618;; ^g^Natural Capital Project, Woods Institute for the Environment, Stanford University, Stanford, CA 94618

**Keywords:** agroecology, field size, pest density, pesticide use, crop yield

## Abstract

Increasing diversity on farms can enhance many key ecosystem services to and from agriculture, and natural control of arthropod pests is often presumed to be among them. The expectation that increasing the size of monocultural crop plantings exacerbates the impact of pests is common throughout the agroecological literature. However, the theoretical basis for this expectation is uncertain; mechanistic mathematical models suggest instead that increasing field size can have positive, negative, neutral, or even nonlinear effects on arthropod pest densities. Here, we report a broad survey of crop field-size effects: across 14 pest species, 5 crops, and 20,000 field years of observations, we quantify the impact of field size on pest densities, pesticide applications, and crop yield. We find no evidence that larger fields cause consistently worse pest impacts. The most common outcome (9 of 14 species) was for pest severity to be independent of field size; larger fields resulted in less severe pest problems for four species, and only one species exhibited the expected trend of larger fields worsening pest severity. Importantly, pest responses to field size strongly correlated with their responses to the fraction of the surrounding landscape planted to the focal crop, suggesting that shared ecological processes produce parallel responses to crop simplification across spatial scales. We conclude that the idea that larger field sizes consistently disrupt natural pest control services is without foundation in either the theoretical or empirical record.

## Significance

Economies of scale in agricultural production continue to promote shifts to larger monocultural plantings of crop plants. Contrary to widely accepted views on resource concentration in monocultures, we find that larger field sizes do not consistently amplify the severity of arthropod pest impacts. Although smaller fields may enhance biodiversity and augment many ecosystem services, including pollination, our analysis shows that simply downsizing the scale of agriculture will not consistently ameliorate pest impacts. Additional work on pest and natural enemy overwintering and movement biology is needed to understand why larger field sizes can amplify, reduce, or have no effect on pest severity across different pest-crop systems.

Agroecology suggests that augmenting the diversity of plant communities in working landscapes can enhance biodiversity and a variety of important ecosystem services, including pollination and natural pest control (1–17). The effects of plant diversity are thought to operate across spatial scales. Within a single field, agroecologists have shown that polycultures, mixtures of different crop species grown within a single field, will often enhance the suppression of pest populations (2, 18). At the single-field scale, agroecologists have warned that increasing the spatial extent of single monocultural crops, a consistent feature of the increasing industrialization of farming (3, 14, 19, 20), will worsen pest problems (2, 4, 10, 11, 17, 21). Field size has thus become a proxy for diverse and sustainable production. And finally, at the landscape level, agroecologists have promoted the retention of natural habitat patches, the cultivation of a greater diversity of crop plant species, and the design of landscapes with greater edge densities (2, 4, 7, 9–12, 15, 16).

The prediction that larger monocultural fields exacerbate pest problems has a somewhat murky origin (22). Most authors seem to refer this expectation to the general idea that concentrating host plant resources either facilitates their exploitation by specialist herbivores (the resource concentration hypothesis; (23)) or impedes the effective action of natural enemies, which may require a diversity of plants to overwinter or acquire alternate prey, nectar, or pollen (the natural enemies hypothesis; 2, 4, 9, 21, 23). Nonetheless, formal mathematical and simulation models that examine the relationship between crop field size and pest dynamics after explicitly incorporating movement processes, overwintering, and predator-prey interactions do not support the expectation that increasing field size will consistently worsen pest problems. Instead, a variety of outcomes is predicted, with larger fields causing pest populations to either increase, decrease, show no response, or exhibit nonlinear dome- or U-shaped responses (22, 24–27).

An extensive experimental literature has established that larger plant patches can have a variety of effects on herbivore densities, with negative and neutral relationships observed as often as positive relationships (9, 24–26, 28–30). But it is unclear whether these experiments, performed only at the very small spatial scales that are tractable for experimentalists, are relevant to the much-larger spatial scales of production agriculture (31). Larger-scale observational studies of patch size effects on herbivores have been reported in natural ecosystems (e.g., 28, 32–35). However, it is again hard to extend these results to agricultural systems, where frequent strong disturbances (e.g., pesticide applications, plowing, and replanting) are likely to amplify the importance of colonization processes relative to in situ birth and death processes.

Surprisingly, direct empirical studies of the effect of commercial agricultural field size on pest pressure are rare. A recent elegant study by Gagic et al. (36) in Australian cotton fields confirmed the conventional wisdom that larger fields are associated with higher pest densities, increased pesticide use, and depressed crop yield. However, three other studies in Swedish willow plantations, Israeli citrus groves, and Canadian soybean fields instead found either no effect of larger fields on pest densities (37, 38) or a negative effect on pest densities (39) and a positive effect on predator densities (37).

Several studies have also attempted to understand relationships between pests and field size by examining pesticide applications, seemingly confirming the agroecological expectation that more pesticides would be applied on larger fields (10, 11). However, this finding may result from field size shaping pest populations or from field size changing the cost-benefit calculus of pesticide use, independent of pest population densities. Indeed, heavier use of pesticides on larger fields may be favored by reduced per-hectare costs of pesticide applications (economies of scale) or because mobile pests represent a “public bad” that can be managed more effectively by individual farmers on a single-field basis if fields are larger and the speed of pest recolonization from adjacent properties is slowed (40). Because an effect of field size on pest pressure could be registered either by changes in observed pest densities or by changes in pesticide use (5, 41), data on pesticide use are most readily understood when combined with data on underlying pest densities. Patterns of pesticide use are also important in their own right, as pesticide applications are an immediate cost for farmers and a key externality of agricultural production (42).

Ultimately, what many view as a core paradigm in agroecology, namely that larger monocultural crop fields worsen the impact of pests, appears to lack clear theoretical or empirical support. To address the paucity of empirical evidence, we compiled several large observational datasets of pest abundances, pesticide applications, and crop yields from commercial agriculture. Together, our datasets encompassed more than 20,000 field years of observations of 14 pest and 1 natural enemy species across both annual and perennial agroecosystems in the United States (cotton and citrus), Peru (potatoes), and Spain (grapes and olives). The pest taxa studied include, for each crop, the arthropod species that generate the greatest economic damage (43–48). Field sizes varied widely both between and within each of the studied cropping systems. Specifically, systems included subsistence farming conducted in very small fields (in Peru, potatoes: median field size = 360 m^2^, range: 50–1,430 m^2^), agriculture being performed at an intermediate level of intensification (in Spain, grapes: median field size = 41,700 m^2^, range: 600–242.78 × 10^4^ m^2^; olives: median field size = 62,700 m^2^, range: 50–2.22 × 10^7^ m^2^), and highly industrialized farming (in California, citrus: median field size = 83,400 m^2^, range: 2,020–57.53 × 10^4^ m^2^; cotton: median field size = 40.47 × 10^4^ m^2^, range: 4,100–239.98 × 10^4^ m^2^).

Our datasets also include information on field size and the amounts of the focal crop and, for four of the five datasets, natural habitat remnants in the broader landscape. To separate the effects of field size from other potentially correlated aspects of agricultural intensification, we included the identity of the farm (RanchID) or the pest control advisor who had responsibility for pest monitoring and control decisions (TechnicianID) in our statistical models. Finally, our dataset did not include information on smaller landscape elements, such as cover crops, insectary plantings, or hedgerows that are often specifically designed to enhance natural pest control. Thus, our analyses do not address the potential efficacy of these landscape elements.

We addressed four questions. First, is the classical expectation for a positive relationship between field size and pest density consistently supported or, instead, are a variety of outcomes (positive, negative, neutral, and nonlinear) observed? Second, how do pesticide application frequencies change as field sizes increase? Third, are crop yields also sensitive to changes in field size? Crop productivity is of central importance both for the agricultural economy and for global food security (4, 5, 9, 42). Fourth, and finally, is there a correlation between pest responses to field size and their responses to amount of the same crop species in the broader, surrounding landscape? Agricultural landscape theory and crop-patch size theory have largely been developed separately; a finding of parallel influences would suggest that common ecological processes may be operating across spatial scales.

## Results

### Effects of Field Size on Pest Densities and Pesticide Use.

**Fig. 1.**
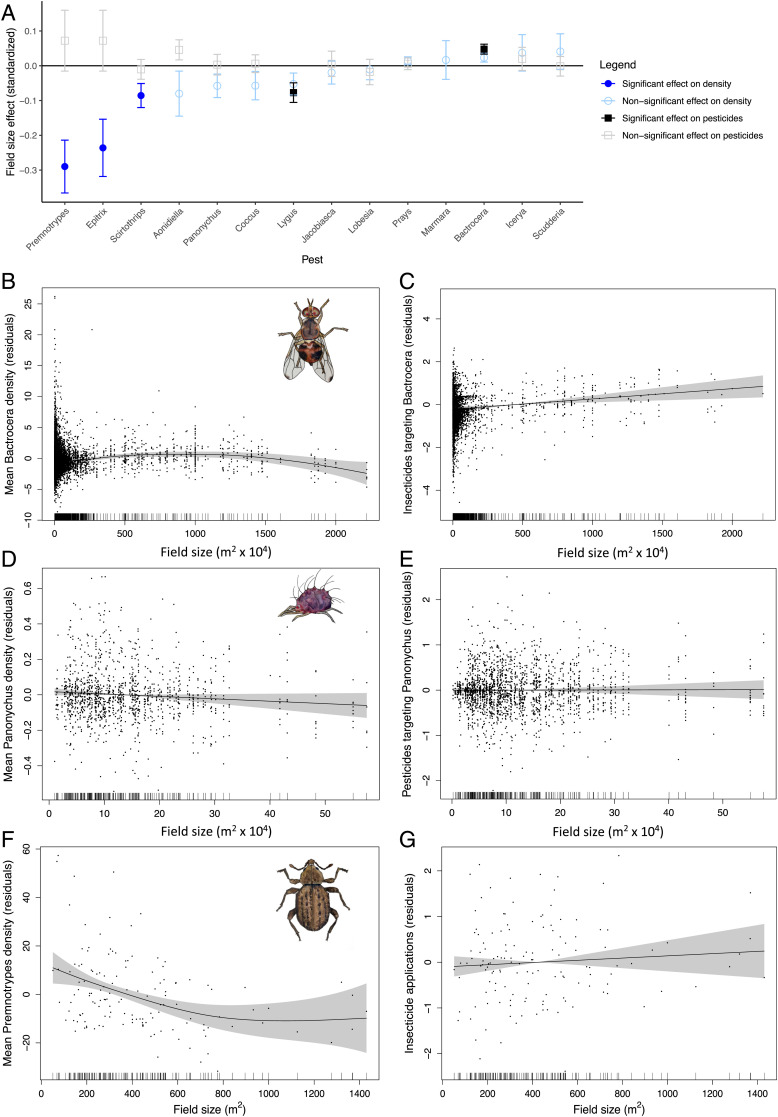
Influence of field size on (*A*) the density (circles) and no. of pesticide applications (squares) targeted to control each of 14 surveyed crop pests (shown are mean ± SE for standardized coefficients from linear terms in GAMMs); filled symbols indicate significant effects. Subsequent panels show examples for which field size is positively (*B* and *C*), neutrally (*D* and *E*), and negatively (*F* and *G*) associated with pest severity. Specifically, panels depict (*B*) the density of the olive fly (*B. oleae*) in Spanish olive orchards (GAMM, effect of field size, *n* = 16,207, *F* = 4.83, *P* < 0.0001), (*C*) the no. of pesticide applications targeting *B. oleae* (effect of field size, *n* = 9,340, χ^2^ = 11.0, *P* = 0.0009), (*D*) the density of the citrus red mite (*P. citri*) in California citrus groves (GAMM, effect of field size, *n* = 1,350, *F* = 2.92, *P* = 0.09 [not significant, NS]), (*E*) the no. of pesticide applications targeting *P. citri* (effect of field size, *n* = 2,176, *F* = 0.01, *P* = 0.92 [NS]), (*F*) the density of Andean potato weevils (*Premnotrypes* spp.) in Peruvian potato fields (GAMM, effect of field size, *n* = 138, *F* = 7.03, *P* = 0.0004), and (*G*) the no. of pesticide applications targeting *Premnotrypes* and *Epitrix* sp. (farmers target both beetle pests with the same insecticide applications; effect of field size, *n* = 138, *F* = 0.68, *P* = 0.41 [NS]). Panels *B*–*G* show residuals on the *y* axis, field size (m^2^) on the *x* axis, the smooth functions fit by the GAMM, and the 95% CIs (shaded area); each point represents a single field year.

Across the 14 pest species surveyed, we found only one, the olive fly (*Bactrocera oleae*), that exhibited higher pest pressure in larger fields (Fig. 1*A*). Pesticide applications targeting *B. oleae* increased in larger fields (Fig. 1*C*), with fly densities increasing initially with field size before declining in the largest fields (Fig. 1*B*). This decline in pest densities in the largest fields might reflect the very heavy pesticide use there, thereby producing a domed density function.

**Fig. 2.**
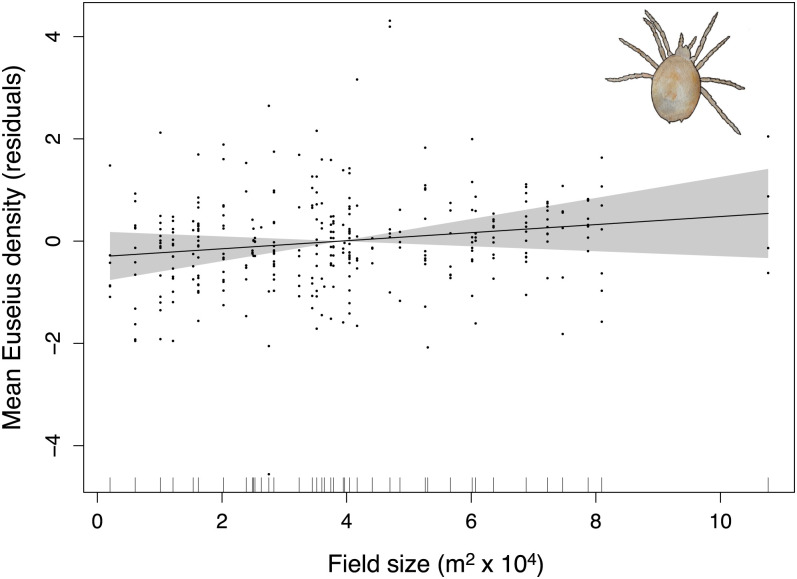
Influence of citrus grove size on the density of predatory mites (*Euseius* spp.; GAMM, effect of field size, *n* = 335, *F* = 1.54, *P* = 0.22 [NS]). Shown are residuals on the *y* axis, field size (m^2^ x 10^4^) on the *x* axis, the function fit by the GAMM, and the 95% CI (uncertainty in the slope value; shaded area). Each point represents a single field year.

The most common outcome of our analyses was for both pest densities and pesticide applications to be independent of field size (true for 9 of the 14 surveyed pests; Fig. 1*A* and *SI Appendix*, Figs. S1–S4). Field size also had no effect on two additional cotton pest species for which we lacked density estimates but could examine targeted pesticide applications (*SI Appendix*, Fig. S5 *A* and *B*). Densities of a key predator in citrus, *Euseius spp.*, were also independent of field size (Fig. 2).

The four remaining pest species exhibited diminishing pressure in larger fields. Three species showed declining densities in larger fields with no change in pesticide applications (*Premnotrypes* and *Epitrix* sp. attacking potatoes, Fig. 1*D* and *SI Appendix*, Fig. S6; *Scirtothrips citri* attacking citrus, *SI Appendix*, Fig. S1*A*), and one species showed no effect of field size on density, despite being targeted with declining nos. of pesticide applications in larger fields (*Lygus hesperus*, *SI Appendix*, Fig. S7). Finally, one cotton pest for which we lacked density data, *Spodoptera exigua*, was targeted with fewer pesticide applications in larger fields (*SI Appendix*, Fig. S5*C*). Thus, we find no overall support for the classical expectation that pest problems should consistently worsen in larger fields.

### Effects of Field Size on Crop Yield.

**Fig. 3.**
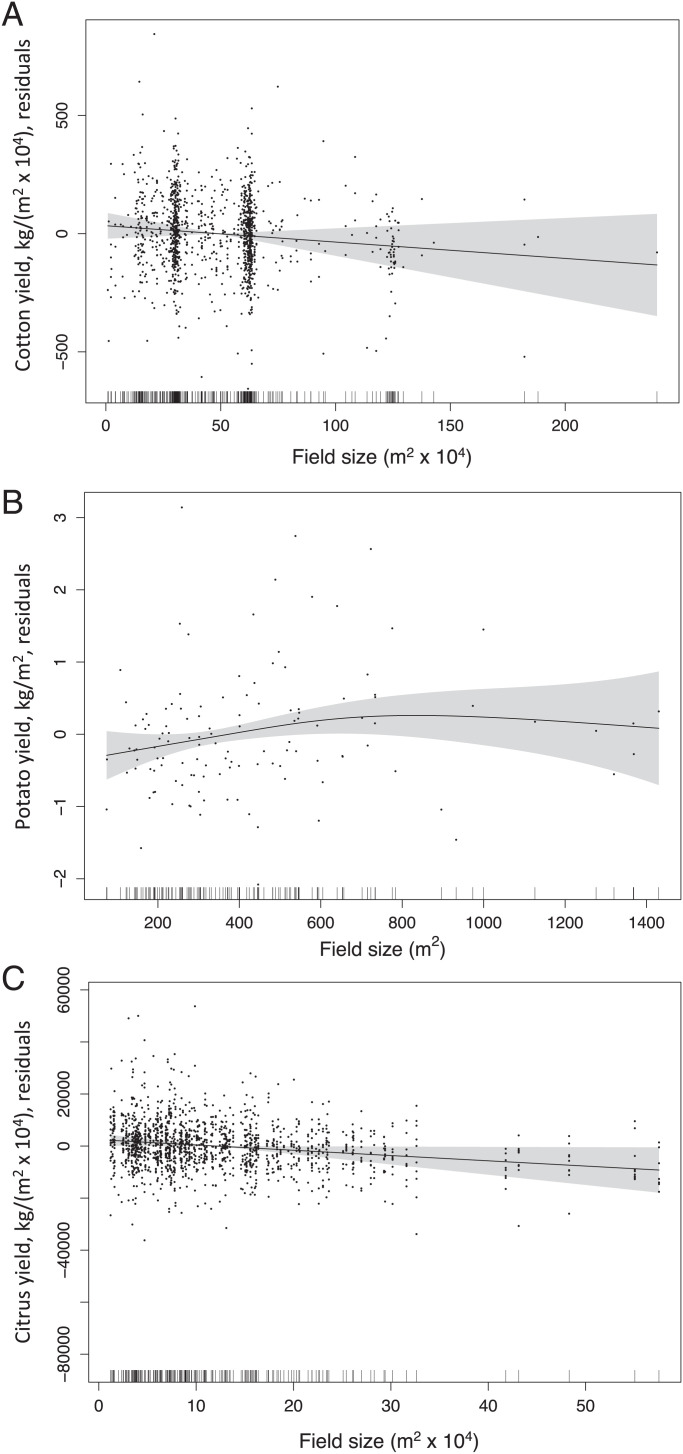
Influence of field size on yield of (*A*) California cotton (GAMM, effect of field size, *n* = 1,236, *F* = 1.50, *P* = 0.22 [NS]), (*B*) Peruvian potatoes (effect of field size, *n* = 125, *F* = 1.85, *P* = 0.14 [NS]), and (*C*) California citrus (effect of field size, *n* = 1,647, *F* = 4.49, *P* = 0.034). Panels show residuals on the *y* axis, the functions fit by the GAMM, and the 95% CIs (shaded area). Each point represents a single field year.

Increasing field size had no significant effect on yield of cotton or potatoes (Fig. 3 *A* and *B*) and a small negative effect on yield of citrus (Fig. 3*C*). Given that none of the citrus pest species showed worsening intensity in larger citrus plantings, lower yields in larger citrus plantings seem unlikely to be linked to the impact of these arthropod pests.

### Field Size and Same Crop in the Landscape.

**Fig. 4.**
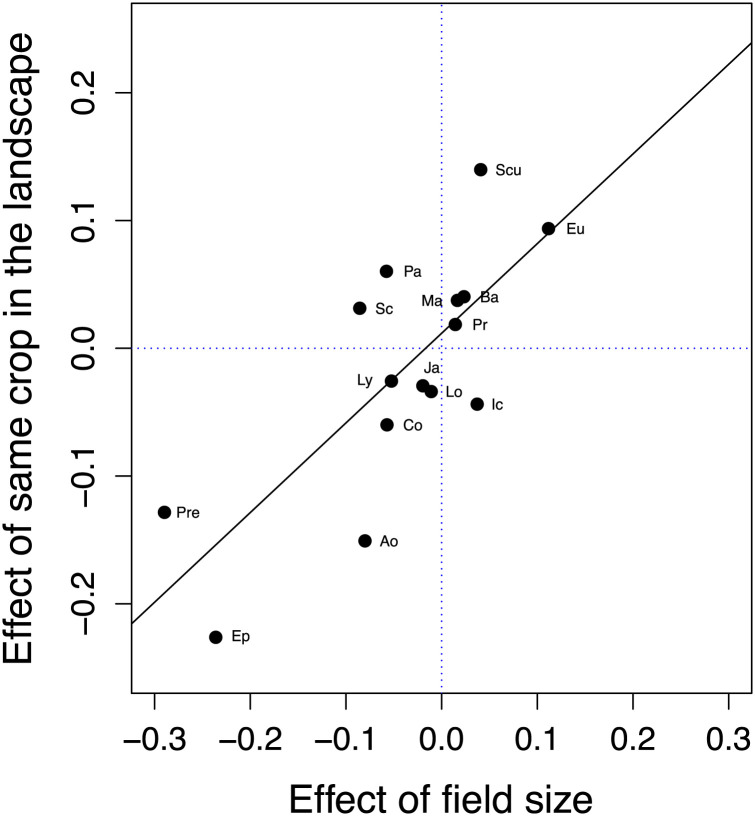
The influence of field size on arthropod density (GAMM coefficient, *β_field size_*) covaries with the influence of the same crop planted in the surrounding landscape on arthropod density (GAMM coefficient, *β_same-crop in landscape_*). Each point represents one of the 15 surveyed arthropod species (linear model, *r* = 0.76, df = 13, *P* = 0.001). Symbols include Ao, *Aonidiella aurantii* (California red scale); Ba, *Bactrocera oleae* (olive fruit fly); Co, *Coccus pseudomagnoliarum* (citricola scale); Ep, *Epitrix* sp. (flea beetles); Eu, *Euseius* sp. (predatory mites); Ic, *Icerya purchasi* (cottony cushion scale); Ja, *Jacobiasca* sp. (leafhoppers); Lo, *Lobesia botrana* (European grapevine moth); Ly, *Lygus hesperus* (western tarnished plant bug); Ma, *Marmara gulosa* (citrus peelminer); Pa, *Panonychus citri* (citrus red mite); Pr, *Prays oleae* (olive moth); Pre, *Premnotrypes* sp. (Andean potato weevil); Sc, *Scirtothrips citri* (California citrus thrips); and Scu, *Scudderia furcata* (fork-tailed bush katydid).

Across our sample of 15 arthropods for which density estimates were available (14 pests and 1 beneficial), we found that our two focal predictors (i) the abundance of the host plant at the smaller spatial scale of a single field (field size) and (ii) the abundance of the same host plant at the larger spatial scale of the surrounding landscape (same crop in landscape) were almost entirely uncorrelated with each other (*SI Appendix*, Table S1) but nevertheless had clearly parallel influences on arthropod densities (Fig. 4). When larger fields were associated with declining pest densities, pest densities also declined as more of the same crop was planted in the surrounding landscape. Conversely, when larger fields were associated with increased pest densities, pest densities also increased as more of the same crop was planted in the surrounding landscape. This suggests that common ecological processes may underlie population responses to the abundance of the host crop plant at multiple spatial scales.

## Discussion

Increasing the spatial extent of monocultural crop plantings is one element of a suite of changes associated with agricultural intensification (3, 14). There is now abundant evidence that increasing field size erodes biodiversity and some of the key ecosystem services provided by working agricultural lands, including pollination services (3, 7–9, 12–14, 17). With respect to pest management, large monocultures create a zone of low plant diversity, where alternate prey and plant-provided resources that are used by natural enemies, including nectar and pollen, are likely to be scarce (e.g., 49, 50). Larger fields are also more difficult for natural enemies with limited dispersal ability to colonize while concentrating the key host plant resource utilized by herbivorous crop pests. It is easy, therefore, to imagine that pest-control services, like other ecosystem services, should decline in larger crop plantings, leading to increases in pest density.

Our results, and previously published studies conducted in production agriculture (36–39), suggest, however, that the severity of pest pressure is not consistently amplified in larger agricultural fields. Larger fields create colonization challenges not only for predators but also for herbivores that may overwinter in other habitats. Moreover, shortages of nectar and pollen resources may not only impact predators but also the generalist pests that utilize these resources (e.g., 51).

Our finding of variable pest responses to increasing field size parallels recent work showing that pest responses to landscape simplification are also context dependent (52, 53). Furthermore, we found that the effects of field size and the effects of the same crop in the landscape are strongly correlated (Fig. 4). This suggests that, though theory for crop field size and theory for compositional effects of the broader agricultural landscape have largely been developed separately, common ecological processes may be operating across scales. Thus, a unified, cross-scale theory on concentration of crop plant resources may be attainable.

### Explaining Variation in Field-Size Effects.

Why might some pests exhibit decreasing densities in larger fields, others show the reverse, and many show no change at all? Models have shown that a suite of traits can push a system toward one pattern or the other, including the presence and efficacy of natural enemies and the overwintering biology and movement capacities of both pests and their natural enemies (21, 22, 25, 27). We rarely know enough, especially about movement of the interacting species, to make clear predictions. However, previous work provides some insight into two of our surveyed species, each of which displayed the unexpected pattern of reduced pest severity in larger crop fields.

First, Andean potato weevils, *Premnotrypes* spp., lack highly effective insect natural enemies and overwinter either in the soil of harvested fields or in potato tuber storage facilities found in each small family farm (54, 55). Adults are wingless and have limited abilities to colonize new plantings. Weevils colonizing new plantings oviposit along field edges, with field centers representing a partial refuge (55, 56). Weevil attack can thus be reduced by planting relatively large fields (i.e., lower perimeter-to-area ratios) in areas surrounded by other potato fields but far from overwintering sources (tuber storage facilities and previous-year potato fields; 55). Indeed, remarkably, the “sectoral farming system” practiced by indigenous Andean farming communities for centuries capitalized on the advantages of growing large, contiguous potato monocultures. Under communal land ownership, communities of farmers would plant their small potato fields directly adjacent to one another. The location of this aggregate monoculture was then moved each year across the landscape, creating diversity in time rather than in space. Under this system, Andean potato weevils were virtually unknown as pests. It was only when the sectoral fallow system was dismantled, replaced by private land ownership and a patchwork of farmers each establishing their own small potato plots on their tiny land holdings, that these weevils emerged as devastating pests (55, 56).

Second, the western tarnished plant bug *L. hesperus* overwinters as an adult and thus cannot overwinter in California cotton fields, where the crop residue must, by state regulations, be destroyed before the winter. *L. hesperus* is winged and mobile, with a partially effective community of natural enemies. Cotton is, however, a relatively poor host plant, supporting a slowly growing population (44). Models predict that, when crops are poor hosts, larger fields may be associated with lower pest densities (25, 27). Furthermore, studies suggest that *L. hesperus* does not “out-disperse” its natural enemy community: the predators appear to have similar, if not greater, mobility than the pest (57). Together, these results may explain why *L. hesperus* elicits fewer pesticide applications in larger fields (*SI Appendix*, Fig. S7) and also why studies have shown that *L. hesperus* densities decline when a cotton field is surrounded by other cotton plantings (58–60).

### Variation Resulting from Farm Management.

Farm management may have shaped the likelihood of observing effects of field size on pest abundances versus pesticide application frequencies. Some farmers apply pesticides on a calendar basis, without reference to in-season fluctuations in pest densities. For the datasets analyzed here, this was the case only for potatoes, where subsistence farmers in Peru do not perform in-season insect sampling. As a result, the effects of field size were expressed as strong effects on observed densities of two pests (*Premnotrypes* spp. and *Epitrix* sp.), with no effect on pesticide applications (Fig. 1*D* and *SI Appendix*, Fig. S6). However, in each of the remaining crops studied, professional pest control advisors performed detailed in-season sampling of pest population densities and made pesticide applications when pest densities reached levels that threatened the crop (e.g., 44, 45). In these cases, underlying effects of field size on pest pressure could be manifested either in increases in pest density, increases in pesticide applications targeting a pest, or both. We saw examples of each of these outcomes (effect on density only: *S. citri*, *SI Appendix*, Fig. S1 *A* and *B*; effect on pesticides only: *L. hesperus*, *SI Appendix*, Fig. S7; effects on both: *B. oleae*, Fig. 1*B*). Thus, as emphasized by O’Rourke and Jones (5), both pest density and targeted pesticide applications should be used as complementary measures of underlying variation in pest pressure when analyzing observational data derived from production agriculture.

### Parallels with Natural Systems.

Interest in how the size of natural habitats affects wild organisms has persisted for decades (61, 62). While conservation biologists are concerned about how smaller, fragmented habitat patches might interfere with their goal of sustaining species of conservation concern, agroecologists are concerned about how larger, monoculture fields might interfere with their goal of suppressing crop pests. Thus, these two communities of scientists have exactly inverted goals but are studying the same process. Here, we have concluded that increasing the size of agricultural habitat patches (crop fields) has a variety of effects on pest populations, with some increasing, some decreasing, and some unaffected. The habitat fragmentation literature seems to support the same conclusion: decreasing the size of natural habitat patches also has a variety of effects on animal populations, with some increasing, some decreasing, and some unaffected (63–66). Though agricultural ecosystems and natural ecosystems differ in many ways, the fact that both show highly varied responses to changing the spatial extent of habitat patches suggests that they may nevertheless share key ecological processes related to use of spatially heterogeneous landscapes.

### Conclusions.

We conclude that the view that larger monocultural crop plantings consistently exacerbate pest problems lacks either theoretical or empirical foundation. This conventional wisdom may have become established because larger field size often goes hand in hand with other features of agricultural intensification that can disrupt pest control (e.g., greater use of agrochemicals). A lack of sufficient testing may then have allowed the idea to persist. However, after introducing statistical control to isolate the effect of field size from covarying features of agricultural intensification, we saw no consistent worsening of pest problems in larger fields, exactly as predicted by mechanistic models (22, 24, 25, 27, 28). Looking forward, we need to replace this tenet of agroecology with a more flexible expectation that field size-pest intensity relationships will vary, depending on the underlying traits of the pest species and their associated natural enemies. Additional work on pest and natural enemy overwintering and movement biology is needed to reveal the mechanisms shaping pest dynamics and to inform sustainable management in different contexts. In those systems where larger fields suffer more frequent pest outbreaks because of the failure of natural enemies to colonize the full expanse of the field, it will be critical to develop effective methods of enhancing natural enemy colonization or retention, including the use of nursery or cover crops, the planting of in-field or near-field refuges (e.g., flowering strips and hedgerows), or augmentative releases of insectary-produced natural enemies. Agroecology needs to move beyond the monoculture/diversity dichotomy and develop a deeper understanding of the mechanistic roles played by different habitat elements at different scales in sustainable pest management. Reducing the size of crop fields may provide several benefits, including bolstering pollination and biodiversity conservation, but is unlikely to consistently improve control of arthropod pests.

## Materials and Methods

### Assembling Datasets from Production Agriculture.

We assembled observational datasets derived from conventional production agriculture describing pest density estimates, pesticide applications targeting the focal arthropod pests, and, when available, yield data. These data were, in all cases but one, originally gathered as part of standard crop monitoring performed by farm staff, independent consultants, or government-employed pest control advisors to assist farmers in making real-time crop and pest management decisions but are now being repurposed for research (i.e., agricultural ecoinformatics; 67). In each case, we analyzed data for all arthropod species for which quantitative density estimates were available. Datasets were gathered for five crops:

(i) Cotton, including *Gossypium hirsutum* (“upland cotton”) and *Gossypium barbadense* (“Pima cotton”), produced in California’s San Joaquin Valley from 1997 to 2008 (44). Density estimates and targeted pesticide applications (*n* = 1,467 and 1,464 field years, respectively) were available only for *L. hesperus* (Hemiptera: Miridae), the western tarnished plant bug. We analyzed pesticide applications targeting *L. hesperus* and also analyzed pesticides applied to control three other pests for which we lacked standardized density estimates: *Tetranychus* spp. (Acari: Tetranychidae; spider mites, *n* = 1,464), *Aphis gossypii* (Hemiptera: Aphididae; the cotton aphid, *n* = 1,464), and *S. exigua* (Lepidoptera: Noctuidae; the beet armyworm, *n* = 1,464). Pesticide application decisions for *Tetranychus* spp., *A. gossypii*, and *S. exigua* were always supported by field observations of high densities, but for these species, sampling methods were less quantitative or not standardized across the industry. Yield data (kg/(m^2^x 10^4^) of cotton lint produced; *n* = 1,236) were also analyzed.
(ii) Citrus, *Citrus* spp., in the San Joaquin Valley of California from 2003 to 2018 (46, 47). Density estimates and targeted pesticide applications were available for seven pest species: *S. citri* (Thysanoptera: Thripidae; California citrus thrips, *n* = 2,205 and 2,176, respectively), *Scudderia furcata* (Orthoptera: Tettigoniidae; fork-tailed bush katydid, *n* = 792 and 2,176, respectively), *Panonychus citri* (Acari: Tetranychidae; citrus red mite, *n* = 1,350 and 2,176, respectively), *Marmara gulosa* (Lepidoptera: Gracillariidae; citrus peelminer, *n* = 774 for density estimates; no pesticides targeted this pest), *Aonidiella aurantii* (Hemiptera: Diaspididae; California red scale, *n* = 793 and 2,176, respectively), *Coccus pseudomagnoliarum* (Hemiptera: Coccidae; citricola scale, *n* = 961 and 2,176, respectively), and *Icerya purchasi* (Hemiptera: Monophlebidae; cottony cushion scale, *n* = 750 and 2,176, respectively). Density data were also available for a single natural enemy taxon: predatory mites, *Euseius* spp. (Acari: Phytoseiidae; *n* = 335). Yield data (kg/(m^2^ x 10^4^) of total fruit harvested; *n* = 1,647) were also analyzed.
(iii) Potato, *Solanum tuberosum*, produced by subsistence farmers in the Andes Mountains of Peru from 2008 to 2009 (43). Density estimates (*n* = 138) and targeted pesticide application nos. (*n* = 138) were available for *Premnotrypes* spp. (Coleoptera: Curculionidae; Andean potato weevils) and *Epitrix* sp. (Coleoptera: Chrysomelidae; flea beetles). Pest density estimates were gathered by researchers, whereas pesticide use and other agronomic practices were recorded by farmers using forms received from researchers (“facilitated ecoinformatics”). Yield data (kg/m^2^; *n* = 125) were also analyzed.
(iv) Grapes, *Vitis vinifera*, in southern Spain from 2006 to 2018. These data were gathered by pest control advisors employed by the Regional Government of Andalusia to promote integrated pest management (RAIF (Red de Alerta e Informacion Fitosanitaria) Network; 45, 48). Density estimates and targeted pesticide applications were available for *Lobesia botrana* (Lepidoptera: Tortricidae; the European grapevine moth, *n* = 996 and 929, respectively) and *Jacobiasca* spp. (Hemiptera: Cicadellidae; leafhoppers, *n* = 1,113 and 929, respectively).
(v) Olives, *Olea europaea*, also in southern Spain from 2006 to 2018 and also part of the RAIF Network (45, 48). Density estimates and targeted pesticide applications were available for *B. oleae* (Diptera: Tephritidae; the olive fruit fly, *n* = 16,207 and 9,340, respectively) and *Prays oleae* (Lepidoptera: Yponomeutidae; the olive moth, *n* = 15,944 and 9,340, respectively).

Thus, in all, density data were available for 14 herbivorous pest species and 1 natural enemy, with targeted pesticide data available for an additional three pest species associated with California cotton. The crops represent a spectrum of disturbance frequencies in agroecosystems, including two annuals (cotton and potato), a deciduous perennial (grape), and two evergreen perennials (olive and citrus). Sampling methods used for each of the focal arthropods are described in *SI Appendix*, Table S2.

Pesticide use data were gathered from farmers or pest control advisors. In all cases except for potato production, the fields were intensively scouted during the growing season, and pesticide application decisions were triggered by detection of elevated pest densities (e.g., 44, 45; for potatoes, pesticides were applied without sampling for pests). Because our goal was to capture the intensity of pest-suppression efforts, we chose the no. of pesticide applications targeting each pest as our primary metric of pesticide use. As emphasized by a National Academy of Sciences workgroup (68), other metrics, such as kg of active ingredient applied per m^2^ or projected measures of vertebrate toxicity, may be preferred for research focused on environmental toxicity endpoints. However, these measures would be less useful here, because different pesticides can have application rates (kg/m^2^) and nontarget toxicity values that vary over orders of magnitude; thus, these alternate metrics are heavily influenced by which material the farmer chooses to apply. Farmers sometimes applied pesticides to only part of a field, in which case we scored a fractional application based on the proportion of the field area treated. Some technicians in the RAIF Network did not report any data on pesticide applications; thus, we retained only those records for which some pesticide applications (against arthropod, pathogen, or weed targets) were reported.

We largely followed the original researchers who assembled these datasets in choosing agronomic and landscape covariates that might influence pest densities. Agronomic covariates were included to provide statistical control for what would otherwise be unexplained variation in our response variables; in many cases, these covariates also provided useful internal checks on overall data quality, showing that the dataset was capable of revealing clear evidence of factors shaping pest densities. Indeed, in all cases, these large datasets had already been shown capable of detecting drivers of pest population densities or crop performance (43–48, 60). We were thus confident that they could reveal effects of field size on pest dynamics. As described previously, field sizes for each crop varied widely, often across orders of magnitude. Field size refers in each case to the land area covered by a continuous monocultural crop planting that was separated from other such plantings by some break (often at least a path or road). Indeed, fields were the basic agricultural management unit, with agronomic and pest-management practices generally implemented at the level of a single field.

Variables describing the landscape always included a measure of the proportion of the area surrounding the focal field that was planted with the same crop as the focal field. This allowed us to quantify the degree to which the host plant resources for our focal herbivores were found in the focal field itself (i.e., field size) or in the area immediately surrounding the focal field (i.e., same crop in the landscape; e.g., 16). For those datasets for which remote sensing data were used to calculate landscape metrics in a circular buffer surrounding the focal field (grape, olive, and citrus), the area of the focal field was subtracted from the areal cover estimates of the focal crop species. Field size and the proportion of the same crop in the landscape were, in all cases, nearly entirely uncorrelated (mean *R*^2^ = 0.017; *SI Appendix*, Table S1), facilitating inferences about their separate influences. Details on methods used to quantify landscape descriptors are presented in *SI Appendix*, Table S3.

### Statistical Modeling.

Separate statistical models were fit for each pest-crop combination and for each response variable (pest density, the no. of pesticide applications targeting the focal pest per year, and crop yield). Our primary predictors of interest, included in all models, were the size of the focal crop field and the proportion of the surrounding landscape planted to the same crop species. Because mathematical models of the influence of field size on pest densities often predict nonlinear effects (25, 27), and because nonlinear effects of patch size on herbivore density have been recorded in small-scale experimentation (e.g., 28), we used generalized additive mixed models (GAMMs) (package *mgcv* in R; 69), which fit flexible curves to the data.

Increased size of monocultural crop plantings is often one element of a suite of changes associated with the intensification of agriculture, which may include practices such as shortened crop-rotation cycles, heavier use of fertilizer and other agrochemical inputs, increased mechanization, increased tillage, and use of improved plant genotypes (3). Each of these practices could independently influence pest population dynamics. Consequently, as emphasized by Larsen and Noack (11), the effects of field size must be separated from this suite of potentially covarying traits to study the effects of field size on pest densities. The most effective way to do this is to include the identity of the ranch (ranchID) as a fixed effect in the statistical model (70, 71). A ranch is a contiguous group of fields, owned and managed by a single farmer and farm staff who make the pest control decisions. ranchID was included in the statistical models for the two datasets for which it was an available variable (cotton and citrus). Inclusion of ranchID as a fixed effect effectively demeans the response variable; thus, our analyses for cotton and citrus pests ask whether variation in field size within a given ranch is associated with deviations in pest density from the mean observed for that particular ranch.

The RAIF datasets for grapes and olives in Spain did not consistently include the identity of the farmer, but because pest control decision making was performed by the government-employed pest control advisor, we included the identity of this advisor (technicianID) in the statistical models for these crops, again as a fixed effect. Fixed effects are less efficient statistically than random effects, because they consume a larger no. of degrees of freedom, but are nonetheless recommended to most confidently control for any unmeasured features of a given farm or technician that could create spurious associations between key predictors (field size and same crop in landscape) and the response variables (70, 71). Finally, for subsistence farmers growing potatoes in Peru, the dataset included only a single field per farm, none of the farms exhibited features of industrialized agriculture, and farming practices were generally similar across farmers (43). Thus, no control for farm-to-farm variation in agronomic practices was included for this case. Because farming intensification is generally expected to worsen pest problems, to the extent that our statistical control for covarying features of intensification was incomplete, we should be more likely to see positive associations between field size and pest densities. As described in the *Results* section, we rarely observed such effects, suggesting that our central, qualitative conclusions were not distorted by covarying aspects of farming intensification.

Our statistical models also included statistical control for (i) repeated observations made on the same fields across years, implemented by including FieldID as a random effect and (ii) spatial autocorrelation of observations, implemented by including a smoothing term for the interaction of longitude and latitude. Separate smooths were fit for each year to accommodate the possibility that a patchy distribution of pest populations might change over time. Tests for spatial autocorrelation of model residuals performed using Moran’s I test (package *ape* in R; 72) confirmed that this approach was effective. Our models used Gaussian distributions for analysis of pest densities, pesticide applications when counts included partial applications (cotton, citrus, potatoes, and grapes), and yield. Poisson distributions (with a log link) were used for the no. of pesticide applications when these were recorded strictly as integer values (olive). All models were fit with the package *mgcv* in R (69). As an example of the full model structure, the model examining influences on densities of *S. citri* was


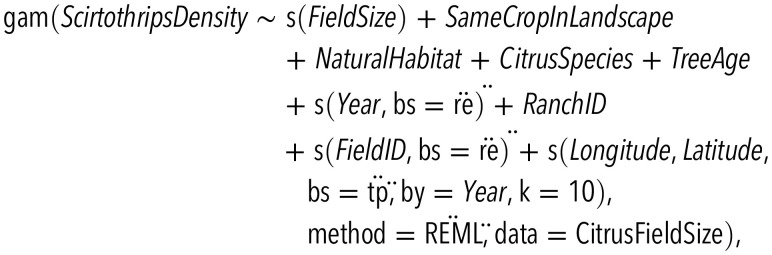


where the variable *FieldSize* was smoothed; *SameCropInLandscape* was measured as the proportion of the surrounding landscape planted to *Citrus* spp.; *NaturalHabitat* was the proportion of the surrounding landscape retaining natural plant communities (in this case, mostly oak woodland); *CitrusSpecies* was a categorical variable describing which citrus species was being grown; *TreeAge* was a continuous variable measuring years since the citrus grove was planted; *Year* was a categorical variable for the observed crop year, included as a random effect; and the remaining terms provided statistical control for the ranch, the repeated observations of a particular field, and the field’s location in space, as described above. Matches to distributional assumptions, as assessed with the *gam.check* function, were sometimes imperfect; we thus interpret our *P* values conservatively throughout. Functions fit by the GAMM collapse to a linear form in the absence of sufficient evidence supporting nonlinearity; in this case, we plot the CI for the GAMM function to display the uncertainty in only the slope value of the field size effect, since it is the slope value, and not uncertainty in the mean, that is the focus of our central hypothesis. Full details of the statistical models used for each crop and descriptions of all covariates are presented in *SI Appendix*, Table S4; fitted models are described in *SI Appendix*, Tables S7–S11.

To be sure that a potentially important effect of field size was not being masked by possible correlations of field size with any of the independent variables that measured features of the surrounding landscape, we conducted two additional sets of analyses. First, we computed the correlations between field size and the proportion of natural habitat in the surrounding landscape, which is the landscape feature most often expected to promote enhanced pest suppression (52). Field size and surrounding natural habitat were, in all cases, nearly entirely uncorrelated (mean *R*^2^ = 0.004; *SI Appendix*, Table S5). Second, we repeated our analyses of the influences of field size on pest densities and pesticide applications with simplified statistical models that omitted all independent variables describing features of the surrounding landscape. These simplified models produced exactly the same conclusions as the full models regarding which relationships were statistically significant and which were not, as well as the signs of any significant relationships (*SI Appendix*, Table S6).

Finally, to examine the relationship between (i) the effect of field size on pest density and (ii) the effect of the same crop in the landscape on pest density across our full set of 15 arthropod species (14 pests plus 1 predator), we repeated our analyses after normalizing the density response variable, the field size predictor, and the same crop in the landscape predictor and fitting a linear function for field size. This allowed us to calculate standardized regression model coefficients for the effects of field size (*β_field size_*) and same crop in the surrounding landscape (*β_same-crop in landscape_*). We then fit a simple linear model to ask whether these two coefficients were correlated.

## Supplementary Material

Supplementary File

## Data Availability

Anonymized [Field data and R scripts] data have been deposited in [DRYAD and Zenodo] (http://datadryad.org/stash/dataset/doi:10.25338/B8006R (73) and https://zenodo.org/record/6574740 (74)). To protect the privacy of data provided by commercial farmers in California, all datasets are anonymized and the latitude-longitude coordinates of sampled cotton and citrus fields are withheld.
