# Supplementary material for: Increasing crop field size does not consistently exacerbate insect pest problems
Source: Proc Natl Acad Sci U S A. 2022 Sep 6;119(37):e2208813119. doi: 10.1073/pnas.2208813119 (PMC9477394; doi:10.1073/pnas.2208813119)
Supplement: Supplementary File [file pnas.2208813119.sapp.pdf]

PNAS

Supplementary Information for

Increasing crop field size does not consistently exacerbate insect pest problems

Jay A. Rosenheim, Emma Cluff, Mia K. Lippey, Bodil N. Cass, Daniel Paredes, Soroush Parsa, Daniel S. Karp, Rebecca Chaplin-Kramer

Corresponding author: Jay Rosenheim
Email: jarosenheim@ucdavis.edu

This PDF file includes:

Figures S1 to S7
Tables S1 to S11
SI References

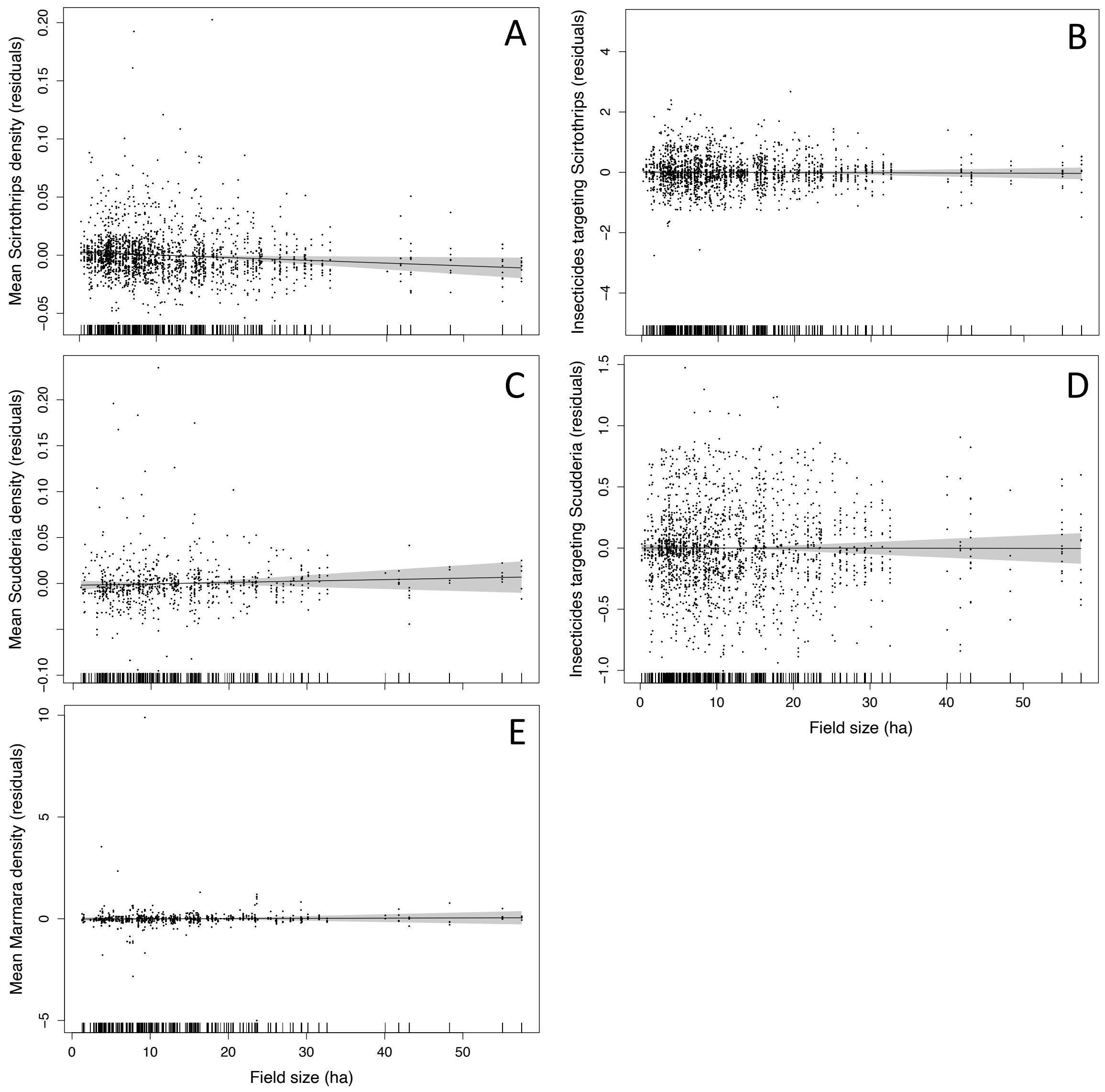

Fig. S1. Influence of citrus grove size on (A) the density of the California citrus thrips *S. citri* (GAM, effect of field size, $N = 2205$, $F = 6.09$, $P = 0.013$); (B) the number of pesticide applications targeting *S. citri* (effect of field size, $N = 2176$, $F = 0.13$, $P = 0.72$ (NS)); (C) the density of the fork-tailed bush katydid *S. furcata* (effect of field size, $N = 792$, $F = 0.64$, $P = 0.42$ (NS)); and (D) the number of pesticide applications targeting *S. furcata* (effect of field size, $N = 2176$, $F = 0.003$, $P = 0.96$ (NS)); (E) the density of the citrus peelminer *M. gulosa* (effect of field size, $N = 774$, $F = 0.09$, $P = 0.77$ (NS)). No pesticide applications were made targeting *M. gulosa*. All panels show residuals on the y-axis, field size (ha) on the x-axis, the smooth functions fit by the GAM model and the 95% confidence intervals (shaded area). Each point represents a single field-year.

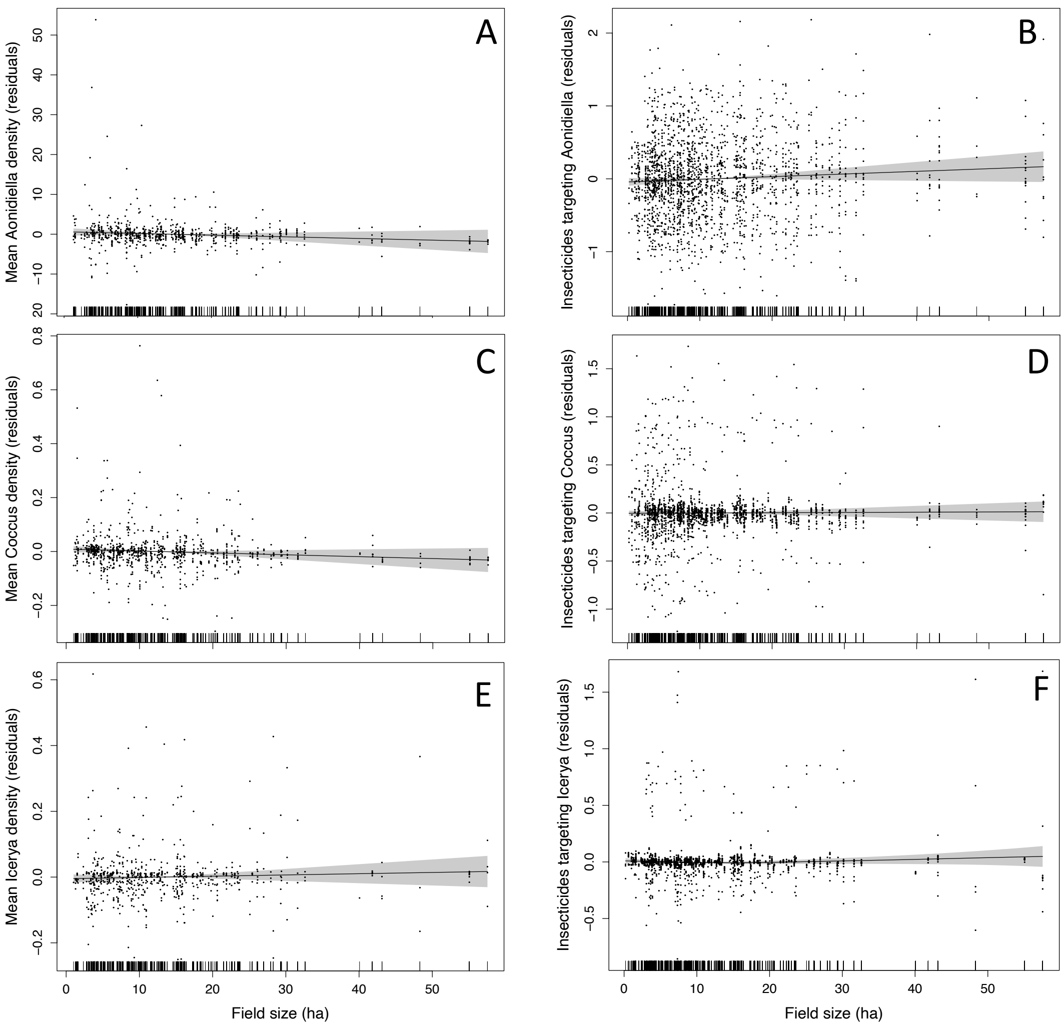

Fig. S2. Influence of citrus grove size on (A) the density of the California red scale *A. aurantii* (GAM, effect of field size, *N* = 793 , *F* = 1.53, *P* = 0.22 (NS)); (B) the number of pesticide applications targeting *A. aurantii* (effect of field size, *N* = 2176 , *F* = 2.51, *P* = 0.11 (NS)); (C) the density of the citricola scale *C. pseudomagnoliarum* (effect of field size, *N* = 961 , *F* = 1.97, *P* = 0.16 (NS)); and (D) the number of pesticide applications targeting *C. pseudomagnoliarum* (effect of field size, *N* = 2176 , *F* = 0.06, *P* = 0.81 (NS)); (E) the density of the cottony cushion scale *I. purchasi* (GAM, effect of field size, *N* = 750 , *F* = 0.50, *P* = 0.48 (NS)); (F) the number of pesticide applications targeting *I. purchasi* (effect of field size, *N* = 2176 , *F* = 1.37, *P* = 0.36 (NS)). All panels show residuals on the y-axis, field size (ha) on the x-axis, the smooth functions fit by the GAM model and the 95% confidence intervals (shaded area). Each point represents a single field-year.

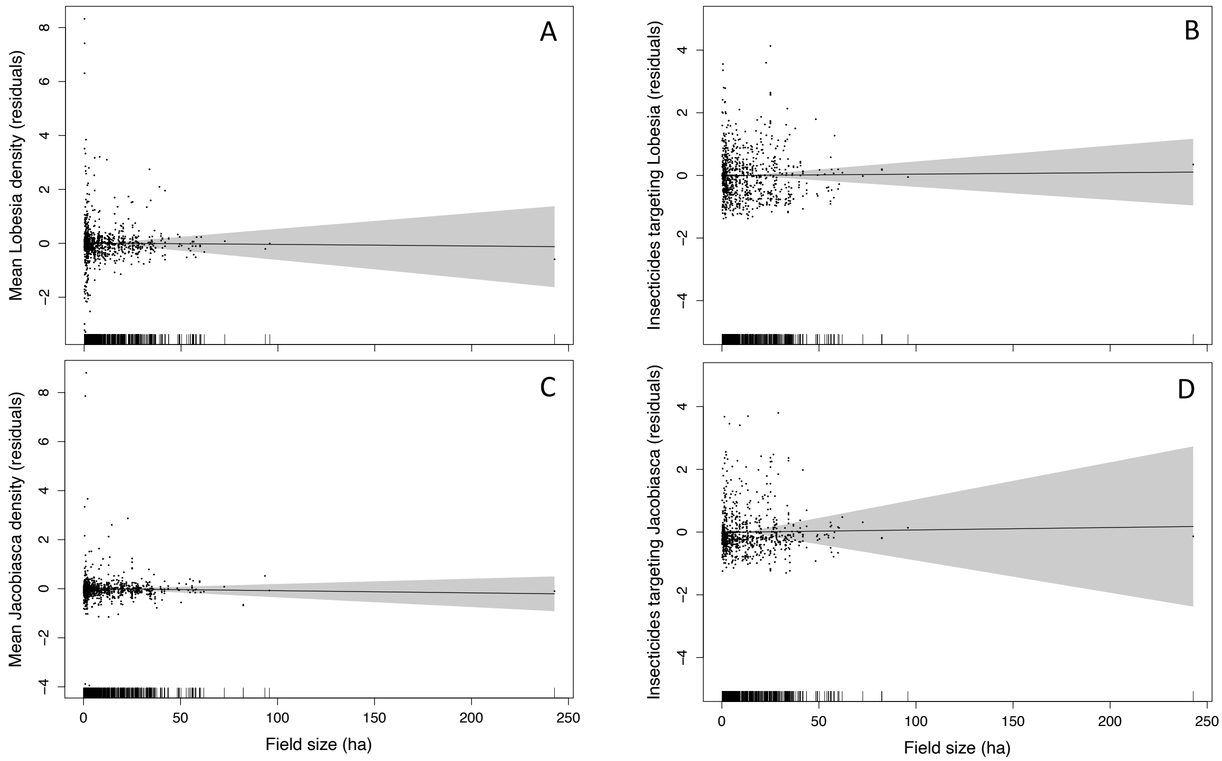

Fig. S3. Influence of grape vineyard size on (A) the density of the European grapevine moth *L. botrana* (GAM, effect of field size, N = 996 , F = 0.02, P = 0.94 (NS)); (B) the number of pesticide applications targeting *L. botrana* (effect of field size, N = 929 , χ^2 = 0.04, P = 0.84 (NS)); (C) the density of the leafhopper *Jacobiasca* sp. (effect of field size, N = 1113 , F = 0.34, P = 0.56 (NS)); and (D) the number of pesticide applications targeting *Jacobiasca* (effect of field size, N = 929 , χ^2 = 0.02, P = 0.89 (NS)). All panels show residuals on the y-axis, field size (ha) on the x-axis, the smooth functions fit by the GAM model and the 95% confidence intervals (shaded area). Each point represents a single field-year.

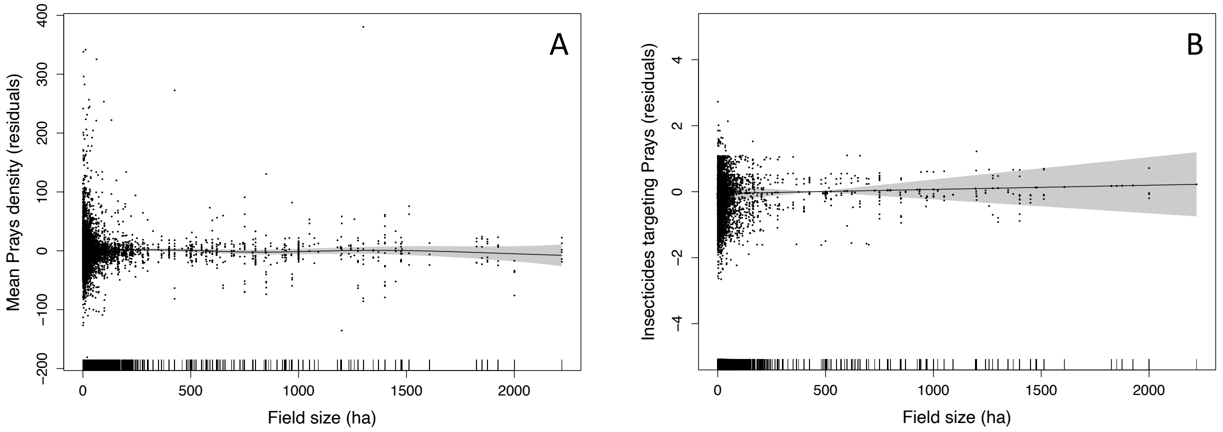

Fig. S4. Influence of olive orchard size on (A) the density of the olive moth *P. oleae* (effect of field size, *N* = 15,944, *F* = 1.99, *P* = 0.073 (NS)); and (B) the number of pesticide applications targeting *P. oleae* (effect of field size, *N* = 9,340 , χ^2 = 0.21, *P* = 0.64 (NS)). Both panels show residuals on the y-axis, field size (ha) on the x-axis, the smooth functions fit by the GAM model and the 95% confidence intervals (shaded area). Each point represents a single field-year.

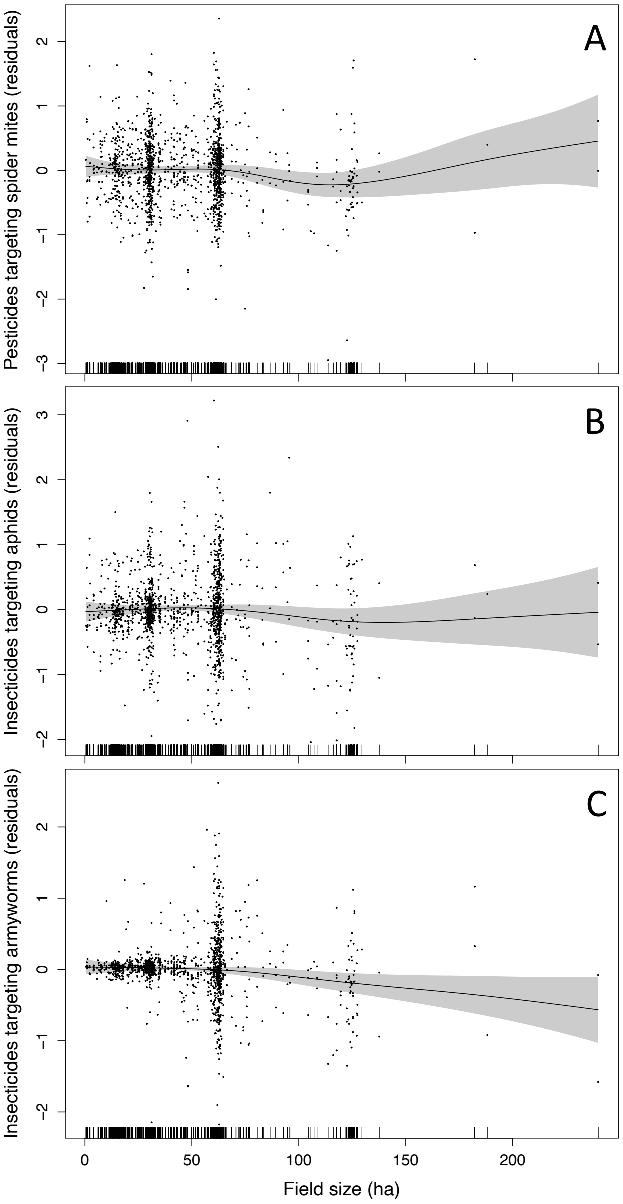

Fig. S5. Influence of cotton field size on the number of pesticide applications targeting (A) spider mites *Tetranychus* spp. (effect of field size, *N* = 1464 , *F* = 2.13, *P* = 0.062 (NS)); (B) the cotton aphid *A. gossypii* (effect of field size, *N* = 1464 , *F* = 1.31, *P* = 0.25 (NS)); and (E) the beet armyworm *S. exigua* (GAM, effect of field size, *N* = 1464 , *F* = 3.13, *P* = 0.024). All panels show residuals on the y-axis and the smooth functions fit by the GAM model and the 95% confidence intervals (shaded area). Each point represents a single field-year.

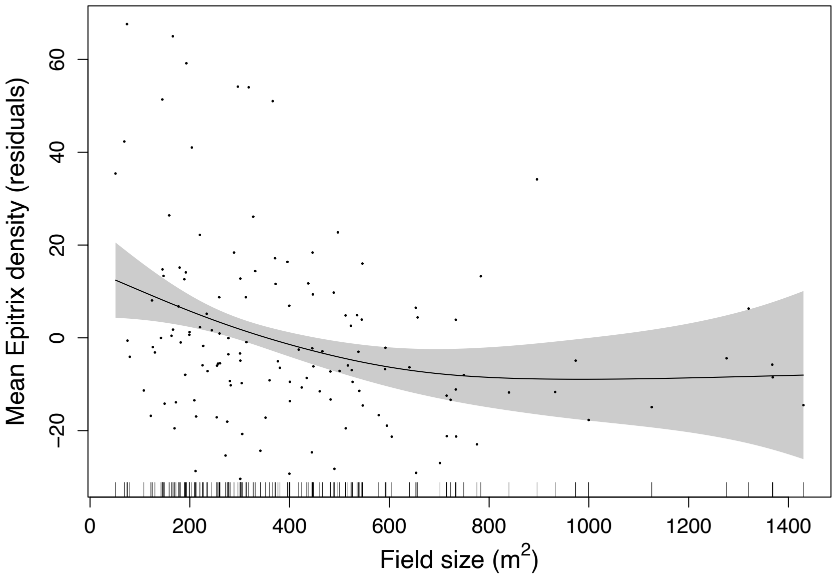

Fig. S6. Influence of potato field size on the density of the fleabeetle *Epitrix* sp. (effect of field size, N = 138 , F = 4.81, P = 0.0064). Shown are residuals on the y-axis, field size (m^2) on the x-axis, the smooth function fit by the GAM model and the 95% confidence interval (shaded area). Each point represents a single field-year.

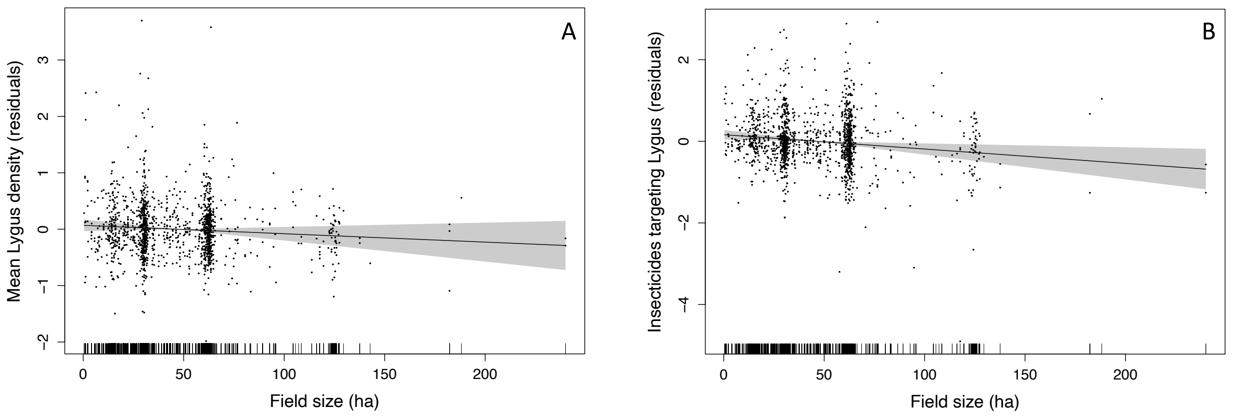

Fig. S7. Influence of cotton field size on (A) the density of the western tarnished plant bug *L. hesperus* (GAM, effect of field size, *N* = 1467 , *F* = 1.76, *P* = 0.18 (NS)); (B) the number of pesticide applications targeting *L. hesperus* (effect of field size, *N* = 1464 , *F* = 7.54, *P* = 0.006). Panels show residuals on the y-axis, field size (ha) on the x-axis, the functions fit by the GAM model and the 95% confidence intervals (shaded area). Each point represents a single field-year.

Table S1: Correlations between focal field size and proportion of the surrounding landscape planted to the same crop.

Crop	Pest taxon	Correlation coefficient (*R*)	R^2
cotton	*Lygus hesperus*	0.192	0.037
potato	*Trymnoprepes* spp.	0.176	0.031
potato	*Epitrix* sp.	0.176	0.031
grapes	*Lobesia botrana*	0.004	0.000
grapes*	*Jacobiasca* sp.	-0.025	0.001
olives	*Bactrocera oleae*	-0.062	0.004
olives	*Prays oleae*	-0.050	0.002
citrus	*Scirtothrips citri*	-0.259	0.067
citrus	*Scudderia furcata*	-0.095	0.007
citrus	*Panonychus citri*	-0.086	0.009
citrus	*Marmara gulosa*	-0.124	0.019
citrus	*Aonidiella aurantii*	-0.114	0.013
citrus	*Coccus pseudomagnoliarum*	-0.137	0.012
citrus	*Icerya purchasi*	-0.108	0.015
citrus	*Euseius* spp.	-0.034	0.001

* It might be expected that the correlation coefficient should be the same value for all pests sampled on a given crop. But, because density estimates were not available for all pests in all fields, each pest produces a different sample of fields and landscapes, and thus the resulting correlation coefficients may vary somewhat across different pests sampled in the same crop.

Table S2. Sampling methods used to estimate densities of 14 pests and one natural enemy in five different agroecosystems. For additional details, see the cited references.

Crop	Pest taxon	Sampling period	Sampling method (reference)
cotton	*Lygus hesperus*	May-July	Mean number of motile *Lygus hesperus* stages captured per sweep net sample. Sweep net samples (ca. 6-12 samples taken during each ca. weekly sample; each sample generated by 50 swings of a standard 38 cm diameter sweep net) with all motile stages (nymphs and adults) counted. (6)
citrus	*Scirtothrips citri*	Weekly, beginning at petal fall (typically late April) and continuing for 8 weeks	Mean proportion of young fruits harboring citrus thrips (presence/absence sampling). Approximately 100 fruits were checked per sample, with sample size varying with the size of the citrus field (range: 25-300 fruits/sample). A weighted mean of successive estimates was calculated (area under the density curve). (1))
citrus	*Scudderia furcata*	Weekly, beginning at petal fall (typically late April) and continuing for 8 weeks	Mean proportion of foliage 'windows' (60 cm x 60 cm areas) harboring fork-tailed bush katydid nymphs or adults (presence/absence sampling); one window was sampled per tree, and approximately 100 trees (range: 10-480, depending on field size) sampled per citrus field. (1)
citrus	*Panonychus citri*	Widely spaced sampling times across the growing season	Mean proportion of individual leaves (both upper and lower surfaces inspected) with motile stages of citrus red mite present (presence/absence sampling). Approximately 100 leaves (range: 10-480, depending on field size) sampled per citrus field. (2)
citrus	*Marmara gulosa*	at harvest	"Bin samples" were taken, in which the visible surfaces of the top layer of just-harvested fruit were visually scanned, and the number of fruit with mines of the citrus peelminer was recorded. The

			median number of bins checked per field per harvest was 214 (range 2-2038). (1)
citrus	*Aonidiella aurantii*	Widely spaced sampling times across the growing season	Mean proportion of trees with scales present (presence/absence sampling). Inner wood was checked early in the season, and outer branches, foliage and fruit examined were checked after the third generation flight of male scales. Approximately 100 trees (range: 25-300, depending on field size) sampled per citrus field. (1)
citrus	*Coccus pseudomagnoliarum*	Widely spaced sampling times across the growing season	Mean proportion of individual leaves (both upper and lower surfaces inspected) with motile stages of citricola scale present (presence/absence sampling). Occasionally, ca. 15-cm portions of branches were used as the sampling unit instead of leaves. Approximately 100 leaves/branches (range: 10-480, depending on field size) sampled per citrus field. (2)
citrus	*Icerya purchasi*	Widely spaced sampling times across the growing season	Mean proportion of trees (60-cm portions of tree trunks or inner branches checked) with nymphal or adult cottony cushion scales present (presence/absence sampling). Approximately 100 trees (range: 10-480, depending on field size) sampled per citrus field. (2)
citrus	*Euseius* spp.	Spring sampling, near the time of petal fall	Firve branch terminals from within the tree canopy were sampled per 10 ha citrus field. On each terminal, the undersides of five leaves were examined to count the motile stages of *Euseius* spp. Density estimates are the mean number of mites per five leaves, averaged to the nearest integer. (3)
potato	*Premnotrypes* spp.	at harvest	Mean proportion of potato tuber biomass infested with weevil larvae at harvest. Mean calculated as a weighted average of tubers sampled from 20 potato

			plants in the field edge (peripheral 3 m wide band) and 20 plants sampled in the field center (rest of field), weighted by the relative areas of these two parts of the field. (5)
potato	*Epitrix* sp.	at harvest	Mean proportion of potato tuber biomass infested with *Epitrix* sp. at harvest. Mean calculated as a weighted average of tubers sampled from 20 potato plants in the field edge (peripheral 3 m wide band) and 20 plants sampled in the field center (rest of field), weighted by the relative areas of these two parts of the field. (5)
grapes	*Lobesia botrana*	March-August	Mean number of moths captured per pheromone trap per day, averaged across the three annual generations. (7)
grapes	*Jacobiasca* sp.	mid-March to late September	Mean number of leafhoppers counts per leaf, averaged across the entire growing season, with approximately weekly sampling.
olives	*Bactrocera oleae*	mid-August to mid-December	Mean fly density (number of flies caught per pheromone-baited yellow sticky trap per day), with approximately weekly sampling. (8)
olives	*Prays oleae*	mid-March to mid-August	Mean number of moths caught per pheromone-baited funnel trap per day, with approximately weekly sampling. (8)

Table S3: Methods used to describe the landscape surrounding focal agricultural fields. For additional details, see the cited references.

Crop	Sampling method (reference)
cotton	Ground-based sampling. The identity of each field located in the eight positions around each focal field (N, NE, E, SE, S, SW, W, NW; the Moore neighborhood) was identified, when possible. Landscape metrics were computed as the proportion of the eight locations occupied by a given crop or land-use type, including alfalfa, almonds, barley, corn, cotton, garbanzo beans, garlic, grapes, lettuce, melons, onions, pistachios, potatoes, safflower, sugar beets, tomatoes, wheat, natural habitat, and fallow. Individual positions could be divided into subunits, as needed. Natural habitat was defined as riparian, desert, or natural pasture that was not irrigated. One land-use category (unknown + other crops) was omitted from the statistical model to avoid over-specification of the landscape. (4)
potato	Ground-based sampling. Three landscape features located within 100 m of the focal were field were mapped: other potato fields, fields that had been planted to potatoes during the previous year, and potato tuber storage units. Storage units and previous-year potato fields were putative sources of overwintered pests. (5)
grape	Remote sensing based sampling. We calculated the proportion of the land surrounding each focal grape vineyard to a distance of 2.0 km from the center of the focal field, using the CORINE Land Cover inventory. Land uses were categorized as grapes, olives, cereals, grasslands, natural shrubs, and forests. A final category (unknown + agriculture) was omitted from the statistical model to avoid over-specification of the landscape. (8)
olive	Remote sensing based sampling. We calculated the proportion of the land surrounding each focal olive grove to a distance of 2.0 km from the center of the focal field, using the CORINE Land Cover inventory. Land uses were categorized as olives, natural shrubs, grasslands, and forests. A final category (unknown + agriculture) was omitted from the statistical model to avoid over-specification of the landscape. (8)
citrus	Remote sensing based sampling. We calculated the proportion of the land surrounding each focal citrus grove to a distance of 1.0 km from the center of the focal field, using the USDA Crop Data Layer. Land uses were categorized as citrus or natural plant communities (mostly oak woodland. A final category (other agriculture, urban, and unknown) was omitted from the statistical model to avoid over-specification of the landscape.

Table S4: Examples of statistical models and explanations of all variables for each of the five crops studied. Model structures were consistent across the three response variables examined: pest density, targeted pesticide applications, and yield.

Statistical model for cotton pests:	*gam(LygusDensity ~ s(FieldSize) + CottonSpecies + RotationCrop + AlfalfaMatrix + AlmondsMatrix + BarleyMatrix + CornMatrix + CottonMatrix + BeansMatrix + GarlicMatrix + GrapesMatrix + LettuceMatrix + MelonsMatrix + OnionsMatrix + PistachiosMatrix + PotatoesMatrix + SafflowerMatrix + SugarbeetsMatrix + TomatoesMatrix + WheatMatrix + NaturalMatrix + FallowMatrix* + s(*Year*, bs = "re") + *RanchID* + s(*FieldID,* bs = "re") + s(*Longitude, Latitude*, bs = "tp", by = *Year,* k = 10), method = "REML", data = *CottonFieldSize*)
Variable	Explanation
LygusDensity	Mean density of *Lygus* spp. observed from May - July
FieldSize	Field size (hectares)
CottonSpecies	Cotton species: *G. hirsutum* (reference level)*, G. barbadense*, or unknown (categorical variable)
RotationCrop	Identity of the crop grown in the field during the year prior to the focal cotton crop
AlfalfaMatrix to *WheatMatrix*	Proportion of the surrounding 8 fields (NW, N, NE, E, SE, S, SW, W) planted to the indicated crop
NaturalMatrix	Proportion of the surrounding 8 fields that harbors natural plant communities, including riparian habitat, desert, and natural pastures
Year	Year the crop was grown (random effect)
RanchID	Unique identifier for each ranch (RanchID0 = reference level), (categorical variable)
FieldID	Unique identifier for each field (random effect)
Longitude, Latitude	Longitude and latitude

Statistical model for citrus pests:	gam(*ScirtothripsDensity* ~ s(*FieldSize*) + *CitrusSpecies* + *PlantingAge* + *CitrusMatrix* + *NaturalMatrix* + s(*Year*, bs = "re") + *RanchID* + s(*FieldID*, bs = "re") + s(*Longitude*, *Latitude*, bs = "tp", by = *Year*, k = 10), method = "REML", data = *CitrusFieldSize*)
Variable	Explanation
ScirtothripsDensity	Mean density of *Scirtothrips citri*
FieldSize	Field size (hectares)
CitrusSpecies	Citrus species: *C. clementina*, *C. clementina* x *sinensis*, *C. limettioides*, *C. limon*, *C. maxima*, *C. meyeri*, *C. paradisi*, *C. reticulata*, *C. sinensis*, *C. tangelo*, *C. unshiu*, or unknown (reference level), (categorical variable)
PlantingAge	Years since the citrus block was planted (1 = year of planting)
CitrusMatrix	Proportion of the surrounding 1-km radius circle planted to citrus
NaturalMatrix	Proportion of the surrounding 1-km radius circle with natural plant communities (mostly oak woodland or natural pasture)
Year	Year the crop was grown (random effect)
RanchID	Unique identifier for each ranch (categorical variable)
FieldID	Unique identifier for each field (random effect)
Longitude, *Latitude*	Longitude and latitude

Statistical model for potato pests:	gam(*PremnotrypesDensity* ~ s(*FieldSize*) + *Cultivar* + *RotationCrop* + *PriorPotatoMatrix* + *Storage* + *CurrentPotatoMatrix* + s(Longitude_X, Latitude_Y, bs = "tp", k = 10) + *Observer*, method = "REML", data = PotatoFieldSize)
Variable	Explanation
PremnotrypesDensity	Mean density of *Premnotrypes* spp.
FieldSize	Field size (m^2)
Cultivar	Potato cultivar grown: yungay or larga (reference level), (categorical variable)
RotationCrop	Identity of the crop grown in the field during the year prior to the focal potato crop (potato, fallow, or other (reference level)), (categorical variable)
PriorPotatoMatrix	Proportion of the surrounding 100 m radius circle area planted to potatoes during the previous year
StorageMatrix	Number of potato storage units within 100 m of the focal potato field
CurrentPotatoMatrix	Proportion of the surrounding 100 m radius circle area planted to potatoes during the current year
Longitude, Latitude	Longitude and latitude
Observer	Identity of the scout who gathered the field data (categorical variable)

Statistical model for grapes pests:	gam(*LobesiaDensity* ~ s(*FieldSize*) + *Cultivar* + *GrapesMatrix* + *CerealsMatrix* + *OlivesMatrix* + *ShrubsMatrix* + *GrasslandMatrix* + *ForestMatrix* + s(*Year*, bs = "re") + *TechnicianID* + s(*FieldID*, bs = "re") + s(*Longitude*, *Latitude*, bs = "tp", by = yr, k = 10), method = "REML", data = *GrapesFieldSize*)
Variable	Explanation
LobesiaDensity	Mean density of *Lobesia botrana*
FieldSize	Field size (hectares)
Cultivar	Grapes cultivar grown: airen, cabernet, chardonnay, jaenblanco, merlot, montepila, muscatel, palomino, pedroximenez, syrah, tempranillo, verdejo, zalema, or unknown (reference level), (categorical variable)
GrapesMatrix	Proportion of the surrounding 2-km radius circle planted to grapes
CerealsMatrix	Proportion of the surrounding 2-km radius circle planted to cereals
OlivesMatrix	Proportion of the surrounding 2-km radius circle planted to olives
ShrubsMatrix	Proportion of the surrounding 2-km radius circle with natural shrubland
GrasslandMatrix	Proportion of the surrounding 2-km radius circle with natural grassland
ForestMatrix	Proportion of the surrounding 2-km radius circle with natural forest
Year	Year the crop was grown (random effect)
TechnicianID	Unique identifier for each pest control advisor technician (categorical variable)
FieldID	Unique identifier for each field (random effect)
Longitude, Latitude	Longitude and latitude

Statistical model for olives pests:	bam(*BactroceraDensity* ~ s(*FieldSize*) + *Cultivar* + *Irrigation* + *Altitude* + *OlivesMatrix* + *ShrubsMatrix* + *GrasslandMatrix* + *ForestMatrix* + s(*Year*, bs = "re") + *TechnicianID* + s(*FieldID*, bs = "re") + s(*Longitude*, *Latitude*, bs = "tp", by = *Year*, k = 10), method = "fREML", data = *OlivesFieldSize*, discrete = TRUE)
Variable	Explanation
BactroceraDensity	Mean density of *Bactrocera oleae*
FieldSize	Field size (hectares)
Cultivar	Olive cultivar grown: alo, arbe, corni, gordal, hoji, lechin, lucio, manz, morona, nevadillo, picolimon, picual, picudo, verdial, or unknown (reference level), (categorical variable)
Irrigation	Irrigation: dryland (reference level), irrigated, or unknown (categorical variable)
Altitude	Altitude, meters above sea level
OlivesMatrix	Proportion of the surrounding 2-km radius circle planted to olives
ShrubsMatrix	Proportion of the surrounding 2-km radius circle with natural shrubland
GrasslandMatrix	Proportion of the surrounding 2-km radius circle with natural grassland
ForestMatrix	Proportion of the surrounding 2-km radius circle with natural forest
Year	Year the crop was grown (random effect)
TechnicianID	Unique identifier for each pest control advisor technician (categorical variable)
FieldID	Unique identifier for each field (random effect)
Longitude, *Latitude*	Longitude and latitude

Table S5: Correlations between focal field size and proportion of the surrounding landscape retaining natural plant communities, two predictors used in analyses of pest densities.

Crop	Pest taxon	Correlation coefficient (*R*)	R^2
cotton	*Lygus hesperus*	-0.043	0.002
potato*	*Trymnoprepes* spp.	NA	NA
potato	*Epitrix* sp.	NA	NA
grapes	*Lobesia botrana*	-0.076	0.006
grapes**	*Jacobiasca* sp.	-0.096	0.009
olives	*Bactrocera oleae*	0.059	0.003
olives	*Prays oleae*	0.099	0.010
citrus	*Scirtothrips citri*	0.063	0.004
citrus	*Scudderia furcata*	-0.042	0.002
citrus	*Panonychus citri*	-0.041	0.002
citrus	*Marmara gulosa*	-0.072	0.005
citrus	*Aonidiella aurantii*	-0.068	0.005
citrus	*Coccus pseudomagnoliarum*	0.006	0.000
citrus	*Icerya purchasi*	0.015	0.000

* Natural habitat in the surrounding landscape was not measured in the study of potato farming in the Andes.

** It might be expected that the correlation coefficient should be the same value for all pests sampled on a given crop. But, because density estimates were not available for all pests in all fields, each pest produces a different sample of fields and landscapes, and thus the resulting correlation coefficients may vary somewhat across different pests sampled in the same crop.

Table S6: Effects of field size on (i) pest density and (ii) the number of annual pesticide applications targeting the pest; shown are coefficients from a Generalized Additive Mixed Model (GAMM) with standardized response and predictor variables, and with all independent variables describing the composition of the surrounding landscape omitted.

		Effect of field size on pest density		Effect of field size on pesticide applications	
Crop	Pest taxon	Coefficient	*P*	Coefficient	*P*
cotton	*Lygus hesperus*	-0.0496	0.139	-0.1056	0.00025
potato	*Trymnoprepes* spp.	-0.3125	0.00011	0.1041	0.239
potato	*Epitrix* sp.	-0.2667	0.00156	0.1041	0.239
grapes	*Lobesia botrana*	-0.0091	0.765	-0.0222	0.546
grapes	*Jacobiasca* sp.	-0.0272	0.422	0.0150	0.677
olives	*Bactrocera oleae*	0.0246	0.063	0.0439	0.0019
olives	*Prays oleae*	-0.0014	0.904	0.0088	0.598
citrus	*Scirtothrips citri*	-0.0888	0.0096	-0.0111	0.694
citrus	*Scudderia furcata*	0.0402	0.427	-0.0068	0.808
citrus	*Panonychus citri*	-0.0455	0.171	-0.0077	0.793
citrus	*Marmara gulosa*	0.0186	0.734	NA	NA
citrus	*Aonidiella aurantii*	-0.0700	0.285	0.0357	0.211
citrus	*Coccus pseudomagnoliarum*	-0.0407	0.320	0.0045	0.860
citrus	*Icerya purchasi*	0.0390	0.450	0.0223	0.499

Table S7. Effects of field size on cotton pest densities, targeted pesticide applications, and yield. Descriptions of GAM modeling results.

1. Effects on *Lygus* spp. densities

> summary(*LygusDensity*)

Family: gaussian
Link function: identity

Formula:

LygusDensity ~ s(FieldSize) + CottonSpecies + RotationCrop + AlfalfaMatrix + AlmondsMatrix + BarleyMatrix + CornMatrix + CottonMatrix + BeansMatrix + GarlicMatrix + GrapesMatrix + LettuceMatrix + MelonsMatrix + OnionsMatrix + PistachiosMatrix + PotatoesMatrix + SafflowerMatrix + SugarbeetsMatrix + TomatoesMatrix + WheatMatrix + NaturalMatrix + FallowMatrix + s(*Year*, bs = "re") + *RanchID* + s(*FieldID,* bs = "re") + s(*Longitude, Latitude*, bs = "tp", by = *Year,* k = 10), method = "REML", data = *CottonFieldSize*

Parametric coefficients:

	Estimate	Std. Error	t value	Pr(>|t|)
(Intercept)	1.175022	0.729867	1.610	0.107673
CottonSpeciesPima	0.030621	0.049184	0.623	0.533675
CottonSpeciesunknown	0.109710	0.119571	0.918	0.359045
RotationCropBarley	-0.383489	0.303588	-1.263	0.206759
RotationCropCarrots	-0.990780	0.305784	-3.240	0.001227 **
RotationCropCorn	-0.122378	0.328786	-0.372	0.709800
RotationCropCotton	-0.173920	0.141013	-1.233	0.217674
RotationCropBeans	-0.143852	0.207918	-0.692	0.489149
RotationCropGarlic	-0.011944	0.172601	-0.069	0.944840
RotationCropLettuce	-0.372837	0.605279	-0.616	0.538024
RotationCropMelons	0.124893	0.430867	0.290	0.771969
RotationCropOnions	0.151031	0.204629	0.738	0.460611
RotationCropPeppers	-1.122957	0.620967	-1.808	0.070788 .
RotationCropPotatoes	-0.222552	0.180854	-1.231	0.218720
RotationCropSafflower	0.095182	0.217745	0.437	0.662097
RotationCropSugarbeets	-0.303558	0.217072	-1.398	0.162237
RotationCropTomatoes	-0.170322	0.155898	-1.093	0.274818
RotationCropunknown	-0.173326	0.151395	-1.145	0.252489
RotationCropWheat	-0.138994	0.146087	-0.951	0.341561
AlfalfaMatrix	-0.203465	0.234326	-0.868	0.385400
AlmondsMatrix	0.468063	0.361778	1.294	0.195983
BarleyMatrix	-0.084569	0.631512	-0.134	0.893491

CornMatrix	-0.295810	0.500272	-0.591	0.554429
CottonMatrix	-0.113164	0.108869	-1.039	0.298798
BeansMatrix	-0.043777	0.444115	-0.099	0.921494
GarlicMatrix	0.077832	0.360379	0.216	0.829045
GrapesMatrix	0.626210	0.759653	0.824	0.409908
LettuceMatrix	0.360723	0.809959	0.445	0.656138
MelonsMatrix	0.008465	0.708647	0.012	0.990471
OnionsMatrix	0.285358	0.373111	0.765	0.444533
PistachiosMatrix	0.400524	0.447747	0.895	0.371213
PotatoesMatrix	1.125906	0.365887	3.077	0.002136 **
SafflowerMatrix	1.924438	0.491497	3.915	9.52e-05 ***
SugarbeetsMatrix	0.582946	0.645207	0.904	0.366436
TomatoesMatrix	0.178792	0.198377	0.901	0.367619
WheatMatrix	0.064255	0.159016	0.404	0.686224
NaturalMatrix	1.299200	0.333117	3.900	0.000101 ***
FallowMatrix	-0.574873	0.358324	-1.604	0.108896
RanchID1	1.475974	2.241382	0.659	0.510333
RanchID2	0.266809	0.487988	0.547	0.584647
RanchID3	0.866227	1.030480	0.841	0.400732
RanchID4	-0.295828	0.403990	-0.732	0.464145
RanchID5	-0.062349	0.254589	-0.245	0.806574
RanchID6	0.292112	0.215972	1.353	0.176448
RanchID7	0.805762	2.042406	0.395	0.693268
RanchID8	0.884273	2.063859	0.428	0.668394
RanchID9	0.180102	0.240807	0.748	0.454658
RanchID10	1.153319	1.397026	0.826	0.409217
RanchID11	-0.300654	0.257158	-1.169	0.242573
RanchID12	1.475075	2.148814	0.686	0.492552
RanchID13	1.817875	2.126348	0.855	0.392757
RanchID14	-0.208794	0.239818	-0.871	0.384123
RanchID15	1.313063	1.419788	0.925	0.355235
RanchID16	-0.166404	0.968551	-0.172	0.863617
RanchID17	0.474188	0.914375	0.519	0.604138
RanchID18	-0.425754	0.259308	-1.642	0.100869
RanchID19	0.438222	0.881179	0.497	0.619057
RanchID20	0.206813	0.205701	1.005	0.314899
RanchID21	0.451590	0.960162	0.470	0.638205
RanchID22	1.253023	0.931258	1.346	0.178706
RanchID23	1.156984	2.092308	0.553	0.580384
RanchID24	0.114354	0.872005	0.131	0.895687
RanchID25	0.410175	0.252472	1.625	0.104496
RanchID26	-0.059376	1.011502	-0.059	0.953200
RanchID27	0.121700	0.319584	0.381	0.703412
RanchID28	0.136094	0.947394	0.144	0.885799

RanchID29	0.348959	0.929829	0.375	0.707506
RanchID30	-0.208615	0.922196	-0.226	0.821071
RanchID31	0.325738	0.288524	1.129	0.259125
RanchID32	0.609736	1.924447	0.317	0.751421
RanchID33	0.458848	0.988591	0.464	0.642627
RanchID34	-1.076808	0.665283	-1.619	0.105796
RanchID35	1.197244	1.415436	0.846	0.397801
RanchID36	0.026520	0.920811	0.029	0.977028
RanchID37	0.815408	1.002001	0.814	0.415928

Signif. codes: 0 '***' 0.001 '**' 0.01 '*' 0.05 '.' 0.1 ' ' 1

Approximate significance of smooth terms:

	edf	Ref.df	F	p-value
s(FieldSize)	1.000	1.000	1.760	0.184911
s(Year)	4.572	11.000	17.404	< 2e-16 ***
s(long,lat):Year1997	2.001	2.002	0.529	0.589965
s(long,lat):Year1998	2.000	2.000	2.932	0.053655 .
s(long,lat):Year1999	2.000	2.000	4.598	0.010243 *
s(long,lat):Year2000	2.455	2.704	1.907	0.245288
s(long,lat):Year2001	2.000	2.000	4.711	0.009154 **
s(long,lat):Year2002	2.813	3.022	1.508	0.214565
s(long,lat):Year2003	6.533	7.365	9.864	< 2e-16 ***
s(long,lat):Year2004	2.001	2.002	9.940	5.20e-05 ***
s(long,lat):Year2005	8.687	8.954	25.113	< 2e-16 ***
s(long,lat):Year2006	8.022	8.734	7.152	< 2e-16 ***
s(long,lat):Year2007	2.001	2.001	10.543	2.92e-05 ***
s(long,lat):Year2008	7.051	7.720	31.705	< 2e-16 ***
s(FieldID)	06.318	516.000	0.272	0.000157 ***

Signif. codes: 0 '***' 0.001 '**' 0.01 '*' 0.05 '.' 0.1 ' ' 1

R-sq.(adj) = 0.684   Deviance explained = 73.4%
-REML = 1305.5 Scale est. = 0.2822   n = 1467

2. Effects on insecticides targeting *Lygus* spp.

> summary(*LygusInsecticides*)

Family: gaussian
Link function: identity

Formula:

LygusInsecticides ~ s(FieldSize) + CottonSpecies + RotationCrop + AlfalfaMatrix + AlmondsMatrix + BarleyMatrix + CornMatrix + CottonMatrix + BeansMatrix + GarlicMatrix + GrapesMatrix + LettuceMatrix + MelonsMatrix + OnionsMatrix + PistachiosMatrix + PotatoesMatrix + SafflowerMatrix + SugarbeetsMatrix + TomatoesMatrix + WheatMatrix + NaturalMatrix + FallowMatrix + s(*Year*, bs = "re") + *RanchID* + s(*FieldID*, bs = "re") + s(*Longitude, Latitude*, bs = "tp", by = *Year,* k = 10), method = "REML", data = *CottonFieldSize*

Parametric coefficients:

	Estimate	Std. Error	t value	Pr(>|t|)
(Intercept)	0.149031	0.806767	0.185	0.85347
CottonSpeciesPima	-0.106006	0.060359	-1.756	0.07927 .
CottonSpeciesunknown	0.127959	0.142802	0.896	0.37039
RotationCropBarley	-0.943026	0.359459	-2.623	0.00880 **
RotationCropCarrots	-0.141281	0.365674	-0.386	0.69929
RotationCropCorn	-0.177030	0.390062	-0.454	0.65001
RotationCropCotton	-0.154320	0.159103	-0.970	0.33225
RotationCropBeans	-0.212573	0.244715	-0.869	0.38519
RotationCropGarlic	-0.003120	0.199541	-0.016	0.98753
RotationCropLettuce	-0.931064	0.726486	-1.282	0.20021
RotationCropMelons	-0.120610	0.513110	-0.235	0.81420
RotationCropOnions	-0.538654	0.243407	-2.213	0.02707 *
RotationCropPeppers	0.839633	0.746872	1.124	0.26113
RotationCropPotatoes	0.152805	0.202989	0.753	0.45172
RotationCropSafflower	-0.044865	0.255969	-0.175	0.86089
RotationCropSugarbeets	-0.188306	0.256687	-0.734	0.46332
RotationCropTomatoes	0.016186	0.177385	0.091	0.92731
RotationCropunknown	-0.082894	0.172909	-0.479	0.63173
RotationCropWheat	-0.180069	0.165763	-1.086	0.27754
AlfalfaMatrix	-0.490557	0.272391	-1.801	0.07194 .
AlmondsMatrix	0.682997	0.417942	1.634	0.10246
BarleyMatrix	0.484539	0.787090	0.616	0.53826
CornMatrix	-0.621388	0.623542	-0.997	0.31917
CottonMatrix	-0.210029	0.128488	-1.635	0.10237
BeansMatrix	-0.338170	0.533585	-0.634	0.52634
GarlicMatrix	-0.578876	0.431291	-1.342	0.17976
GrapesMatrix	0.246438	0.907186	0.272	0.78593
LettuceMatrix	-2.682241	0.966030	-2.777	0.00557 **
MelonsMatrix	-2.065852	0.855629	-2.414	0.01590 *
OnionsMatrix	0.755166	0.453574	1.665	0.09616 .
PistachiosMatrix	-0.185086	0.519218	-0.356	0.72154
PotatoesMatrix	0.688104	0.430484	1.598	0.11018
SafflowerMatrix	3.275501	0.588568	5.565	3.17e-08 ***
SugarbeetsMatrix	-0.469802	0.788411	-0.596	0.55135
TomatoesMatrix	-0.474846	0.235792	-2.014	0.04423 *

WheatMatrix	-0.377139	0.189639	-1.989	0.04694 *
NaturalMatrix	0.739417	0.383765	1.927	0.05423 .
FallowMatrix	-0.826141	0.426821	-1.936	0.05313 .
RanchID1	0.615879	2.556986	0.241	0.80970
RanchID2	-0.005319	0.554615	-0.010	0.99235
RanchID3	1.880433	1.177376	1.597	0.11047
RanchID4	-0.205992	0.442995	-0.465	0.64201
RanchID5	0.544074	0.281798	1.931	0.05373 .
RanchID6	0.151373	0.227893	0.664	0.50666
RanchID7	0.329247	2.327345	0.141	0.88752
RanchID8	0.573650	2.355834	0.244	0.80765
RanchID9	-0.052173	0.263428	-0.198	0.84303
RanchID10	2.018089	1.590882	1.269	0.20483
RanchID11	0.705960	0.287222	2.458	0.01410 *
RanchID12	0.955729	2.450375	0.390	0.69657
RanchID13	0.444276	2.424624	0.183	0.85464
RanchID14	0.057111	0.266648	0.214	0.83044
RanchID15	2.252079	1.617277	1.393	0.16400
RanchID16	1.176254	1.101587	1.068	0.28581
RanchID17	1.228289	1.041436	1.179	0.23844
RanchID18	0.644268	0.289814	2.223	0.02638 *
RanchID19	1.283522	1.003633	1.279	0.20117
RanchID20	0.197648	0.216169	0.914	0.36071
RanchID21	1.528214	1.091521	1.400	0.16172
RanchID22	1.843918	1.060500	1.739	0.08232 .
RanchID23	0.721074	2.386574	0.302	0.76259
RanchID24	1.601967	0.995119	1.610	0.10767
RanchID25	0.153350	0.277077	0.553	0.58004
RanchID26	1.400465	1.150478	1.217	0.22371
RanchID27	-0.152796	0.360394	-0.424	0.67166
RanchID28	1.431147	1.077696	1.328	0.18442
RanchID29	1.623866	1.061084	1.530	0.12616
RanchID30	1.618984	1.050842	1.541	0.12364
RanchID31	0.026857	0.319517	0.084	0.93302
RanchID32	0.571535	2.203255	0.259	0.79536
RanchID33	1.508852	1.126974	1.339	0.18085
RanchID34	-0.979219	0.744349	-1.316	0.18856
RanchID35	2.108105	1.604495	1.314	0.18912
RanchID36	1.669089	1.051959	1.587	0.11283
RanchID37	1.609812	1.142164	1.409	0.15894

Signif. codes: 0 ‘***’ 0.001 ‘**’ 0.01 ‘*’ 0.05 ‘.’ 0.1 ‘ ’ 1

Approximate significance of smooth terms:

	edf	Ref.df	F	p-value
s(hectares)	1.000	1.001	7.537	0.00613 **
s(Year)	3.097	11.000	8.686	< 2e-16 ***
s(long,lat):Year1997	2.002	2.004	0.997	0.36982
s(long,lat):Year1998	2.000	2.000	13.510	1.68e-06 ***
s(long,lat):Year1999	2.000	2.001	1.272	0.28069
s(long,lat): Year2000	2.000	2.001	1.889	0.15167
s(long,lat): Year2001	2.000	2.001	11.815	8.36e-06 ***
s(long,lat): Year2002	2.839	3.053	3.900	0.00852 **
s(long,lat): Year2003	8.325	8.729	22.268	< 2e-16 ***
s(long,lat): Year2004	2.014	2.020	10.614	2.23e-05 ***
s(long,lat): Year2005	8.303	8.854	8.042	< 2e-16 ***
s(long,lat): Year2006	5.380	6.486	1.176	0.41381
s(long,lat):Year2007	7.569	8.331	4.983	1.99e-06 ***
s(long,lat):Year2008	8.713	8.909	60.168	< 2e-16 ***
s(FieldID)	9.273	512.000	0.019	0.37720

Signif. codes: 0 ‘***’ 0.001 ‘**’ 0.01 ‘*’ 0.05 ‘.’ 0.1 ‘ ’ 1

R-sq.(adj) = 0.725   Deviance explained = 75.2%
-REML = 1555.1 Scale est. = 0.43765   n = 1464

<u>3. Effects on insecticides targeting *Tetranychus* spp.</u>

> summary(*TetranychusInsecticides*)

Family: gaussian
Link function: identity

Formula:
TetranychusInsecticides ~ s(FieldSize) + CottonSpecies + RotationCrop + AlfalfaMatrix + AlmondsMatrix + BarleyMatrix + CornMatrix + CottonMatrix + BeansMatrix + GarlicMatrix + GrapesMatrix + LettuceMatrix + MelonsMatrix + OnionsMatrix + PistachiosMatrix + PotatoesMatrix + SafflowerMatrix + SugarbeetsMatrix + TomatoesMatrix + WheatMatrix + NaturalMatrix + FallowMatrix + s(*Year*, bs = "re") + *RanchID* + s(*FieldID*, bs = "re") + s(*Longitude, Latitude*, bs = "tp", by = *Year,* k = 10), method = "REML", data = *CottonFieldSize*

Family: gaussian
Link function: identity

Parametric coefficients:

	Estimate	Std. Error	t value	Pr(>|t|)
(Intercept)	0.659599	0.652295	1.011	0.312105
CottonSpeciesPima	-0.516965	0.049075	-10.534	< 2e-16 ***
CottonSpeciesunknown	-0.332823	0.116256	-2.863	0.004264 **
RotationCropBarley	-0.100884	0.291382	-0.346	0.729228
RotationCropCarrots	0.132288	0.296610	0.446	0.655669
RotationCropCorn	0.326006	0.317286	1.027	0.304379
RotationCropCotton	-0.022059	0.129595	-0.170	0.864864
RotationCropBeans	-0.146946	0.198865	-0.739	0.460085
RotationCropGarlic	-0.028401	0.162283	-0.175	0.861097
RotationCropLettuce	0.817343	0.590221	1.385	0.166343
RotationCropMelons	-0.022630	0.417771	-0.054	0.956809
RotationCropOnions	0.215223	0.198050	1.087	0.277361
RotationCropPeppers	1.081194	0.593232	1.823	0.068596 .
RotationCropPotatoes	0.218847	0.163504	1.338	0.180967
RotationCropSafflower	0.425160	0.208533	2.039	0.041666 *
RotationCropSugarbeets	-0.048805	0.208341	-0.234	0.814821
RotationCropTomatoes	-0.060269	0.144564	-0.417	0.676817
RotationCropunknown	-0.117054	0.140900	-0.831	0.406259
RotationCropWheat	-0.055369	0.134893	-0.410	0.681529
AlfalfaMatrix	0.159151	0.221864	0.717	0.473291
AlmondsMatrix	1.032298	0.337596	3.058	0.002274 **
BarleyMatrix	-1.437168	0.636740	-2.257	0.024165 *
CornMatrix	0.315363	0.493039	0.640	0.522522
CottonMatrix	0.207948	0.103487	2.009	0.044695 *
BeansMatrix	0.288746	0.433053	0.667	0.505036
GarlicMatrix	0.700106	0.348699	2.008	0.044870 *
GrapesMatrix	-0.216447	0.735268	-0.294	0.768515
LettuceMatrix	-1.117550	0.785742	-1.422	0.155177
MelonsMatrix	-0.014530	0.695863	-0.021	0.983344
OnionsMatrix	0.189036	0.367242	0.515	0.606818
PistachiosMatrix	0.223868	0.422306	0.530	0.596126
PotatoesMatrix	-0.119001	0.347152	-0.343	0.731809
SafflowerMatrix	-0.256004	0.475836	-0.538	0.590661
SugarbeetsMatrix	0.619514	0.634939	0.976	0.329387
TomatoesMatrix	0.262320	0.192106	1.365	0.172328
WheatMatrix	0.379559	0.153553	2.472	0.013566 *
NaturalMatrix	0.198309	0.310133	0.639	0.522653
FallowMatrix	0.325883	0.347820	0.937	0.348965
RanchID1	2.078798	2.052457	1.013	0.311323
RanchID2	0.061631	0.445534	0.138	0.890000
RanchID3	1.062697	0.948524	1.120	0.262758
RanchID4	-0.818542	0.363448	-2.252	0.024474 *

RanchID5	0.756874	0.223831	3.381	0.000742 ***
RanchID6	0.082400	0.183408	0.449	0.653312
RanchID7	1.894328	1.870197	1.013	0.311290
RanchID8	1.119829	1.893318	0.591	0.554310
RanchID9	0.104239	0.210197	0.496	0.620037
RanchID10	-0.152702	1.281212	-0.119	0.905146
RanchID11	0.666931	0.228801	2.915	0.003618 **
RanchID12	1.075052	1.967927	0.546	0.584960
RanchID13	0.981213	1.948012	0.504	0.614556
RanchID14	0.875297	0.214630	4.078	4.81e-05 ***
RanchID15	-0.027256	1.302610	-0.021	0.983309
RanchID16	1.967541	0.890112	2.210	0.027244 *
RanchID17	1.783638	0.837767	2.129	0.033434 *
RanchID18	0.686499	0.233678	2.938	0.003362 **
RanchID19	2.097601	0.807470	2.598	0.009487 **
RanchID20	-0.006613	0.175117	-0.038	0.969883
RanchID21	1.805099	0.882002	2.047	0.040894 *
RanchID22	1.406246	0.852602	1.649	0.099310 .
RanchID23	1.225946	1.917727	0.639	0.522757
RanchID24	1.525343	0.797778	1.912	0.056092 .
RanchID25	-0.144120	0.222121	-0.649	0.516558
RanchID26	2.393165	0.932575	2.566	0.010391 *
RanchID27	0.226098	0.289863	0.780	0.435520
RanchID28	1.644153	0.867236	1.896	0.058196 .
RanchID29	0.836787	0.854846	0.979	0.327819
RanchID30	1.399745	0.847967	1.651	0.099034 .
RanchID31	0.268024	0.256814	1.044	0.296837
RanchID32	1.389627	1.779011	0.781	0.434869
RanchID33	2.131418	0.908487	2.346	0.019116 *
RanchID34	-1.426739	0.598059	-2.386	0.017190 *
RanchID35	-0.123762	1.292532	-0.096	0.923732
RanchID36	2.054013	0.846252	2.427	0.015348 *
RanchID37	1.215389	0.920602	1.320	0.186992

Signif. codes: 0 ‘***’ 0.001 ‘**’ 0.01 ‘*’ 0.05 ‘.’ 0.1 ‘ ’ 1

Approximate significance of smooth terms:

	edf	Ref.df	F	p-value
s(FieldSize)	4.042159	5.011	2.125	0.0616 .
s(Year)	4.230511	11.000	5.500	< 2e-16 ***
s(long,lat):Year1997	2.000301	2.001	0.261	0.7704
s(long,lat):Year1998	2.580762	2.856	0.505	0.6516
s(long,lat):Year1999	2.000198	2.000	1.695	0.1841
s(long,lat):Year2000	2.000237	2.000	0.074	0.9285

s(long,lat):Year2001	2.000170	2.000	1.296	0.2740
s(long,lat):Year2002	2.000164	2.000	1.942	0.1438
s(long,lat):Year2003	7.958752	8.466	10.401	< 2e-16 ***
s(long,lat):Year2004	6.471480	7.622	9.168	< 2e-16 ***
s(long,lat):Year2005	2.008043	2.012	0.781	0.4566
s(long,lat):Year2006	8.078281	8.763	4.821	2.31e-06 ***
s(long,lat):Year2007	6.402664	7.450	8.686	< 2e-16 ***
s(long,lat):Year2008	4.542328	5.358	7.302	1.20e-06 ***
s(FieldID)	0.001144	512.000	0.000	0.9504

Signif. codes: 0 '***' 0.001 '**' 0.01 '*' 0.05 '.' 0.1 ' ' 1

R-sq.(adj) = 0.694   Deviance explained = 72.1%
-REML =   1261 Scale est. = 0.2916   n = 1464

4. Effects on insecticides targeting *Spodoptera exigua*

> summary(*SpodopteraInsecticides*)

Family: gaussian
Link function: identity

Formula:
SpodopteraInsecticides ~ s(FieldSize) + CottonSpecies + RotationCrop + AlfalfaMatrix + AlmondsMatrix + BarleyMatrix + CornMatrix + CottonMatrix + BeansMatrix + GarlicMatrix + GrapesMatrix + LettuceMatrix + MelonsMatrix + OnionsMatrix + PistachiosMatrix + PotatoesMatrix + SafflowerMatrix + SugarbeetsMatrix + TomatoesMatrix + WheatMatrix + NaturalMatrix + FallowMatrix + s(*Year*, bs = "re") + *RanchID* + s(*FieldID*, bs = "re") + s(*Longitude, Latitude*, bs = "tp", by = *Year,* k = 10), method = "REML", data = *CottonFieldSize*

Family: gaussian
Link function: identity

Parametric coefficients:

	Estimate	Std. Error	t value	Pr(>|t|)
(Intercept)	-0.5068581	0.5079031	-0.998	0.318489
CottonSpeciesPima	-0.1838115	0.0366408	-5.017	5.97e-07 ***
CottonSpeciesunknown	0.0499802	0.0868094	0.576	0.564884
RotationCropBarley	-0.1362538	0.2174763	-0.627	0.531080
RotationCropCarrots	0.0326679	0.2219098	0.147	0.882987
RotationCropCorn	-0.1142530	0.2364196	-0.483	0.628988
RotationCropCotton	-0.0032672	0.0962694	-0.034	0.972932

RotationCropBeans	0.1908436	0.1481018	1.289	0.197762
RotationCropGarlic	-0.0700233	0.1207928	-0.580	0.562217
RotationCropLettuce	1.5081556	0.4400871	3.427	0.000629 ***
RotationCropMelons	0.2031207	0.3109514	0.653	0.513725
RotationCropOnions	-0.1112649	0.1474722	-0.754	0.450694
RotationCropPeppers	0.0763205	0.4468020	0.171	0.864395
RotationCropPotatoes	-0.0099028	0.1227037	-0.081	0.935689
RotationCropSafflower	0.1899915	0.1547519	1.228	0.219770
RotationCropSugarbeets	-0.0079475	0.1555134	-0.051	0.959250
RotationCropTomatoes	0.0274157	0.1075279	0.255	0.798791
RotationCropunknown	0.0434413	0.1045178	0.416	0.677744
RotationCropWheat	-0.0001716	0.1002555	-0.002	0.998635
AlfalfaMatrix	0.0344976	0.1654249	0.209	0.834840
AlmondsMatrix	-0.1734406	0.2537905	-0.683	0.494473
BarleyMatrix	-2.0145853	0.4754826	-4.237	2.42e-05 ***
CornMatrix	0.1428202	0.3739769	0.382	0.702600
CottonMatrix	0.0741165	0.0778194	0.952	0.341059
BeansMatrix	0.8589193	0.3239602	2.651	0.008113 **
GarlicMatrix	0.4248371	0.2640270	1.609	0.107839
GrapesMatrix	-1.1202014	0.5490053	-2.040	0.041506 *
LettuceMatrix	0.5474073	0.5859665	0.934	0.350372
MelonsMatrix	-0.1547583	0.5191486	-0.298	0.765673
OnionsMatrix	-0.2339213	0.2732134	-0.856	0.392049
PistachiosMatrix	-0.0190465	0.3151479	-0.060	0.951817
PotatoesMatrix	0.0577864	0.2603962	0.222	0.824412
SafflowerMatrix	-0.4935344	0.3557509	-1.387	0.165582
SugarbeetsMatrix	-0.0387045	0.4749452	-0.081	0.935062
TomatoesMatrix	-0.0855931	0.1434074	-0.597	0.550707
WheatMatrix	0.2170543	0.1147432	1.892	0.058754 .
NaturalMatrix	0.2404696	0.2309430	1.041	0.297948
FallowMatrix	-0.7460075	0.2594831	-2.875	0.004105 **
RanchID1	0.5852048	1.5345141	0.381	0.702996
RanchID2	-0.0087233	0.3370805	-0.026	0.979358
RanchID3	2.1651716	0.7107644	3.046	0.002363 **
RanchID4	0.1150940	0.2783575	0.413	0.679325
RanchID5	-0.0453768	0.1697062	-0.267	0.789215
RanchID6	-0.0685740	0.1368464	-0.501	0.616382
RanchID7	0.6115090	1.3975248	0.438	0.661772
RanchID8	0.6002242	1.4155175	0.424	0.671611
RanchID9	0.0022443	0.1574102	0.014	0.988626
RanchID10	0.6278027	0.9605296	0.654	0.513482
RanchID11	0.1797631	0.1768402	1.017	0.309563
RanchID12	0.5988777	1.4716701	0.407	0.684119
RanchID13	0.5872232	1.4560836	0.403	0.686800

RanchID14	-0.0178624	0.1609299	-0.111	0.911637
RanchID15	0.5481363	0.9762028	0.561	0.574552
RanchID16	2.7233382	0.6659063	4.090	4.58e-05 ***
RanchID17	2.9048032	0.6283764	4.623	4.16e-06 ***
RanchID18	0.1808289	0.1792310	1.009	0.313199
RanchID19	2.6163763	0.6047341	4.326	1.63e-05 ***
RanchID20	0.0147795	0.1299881	0.114	0.909494
RanchID21	2.4207585	0.6593802	3.671	0.000251 ***
RanchID22	2.2049663	0.6393623	3.449	0.000581 ***
RanchID23	0.6285035	1.4340655	0.438	0.661264
RanchID24	2.1367058	0.5982360	3.572	0.000367 ***
RanchID25	0.0026768	0.1680411	0.016	0.987293
RanchID26	2.0380290	0.6950064	2.932	0.003421 **
RanchID27	-0.0184591	0.2174143	-0.085	0.932352
RanchID28	2.7063526	0.6499112	4.164	3.33e-05 ***
RanchID29	2.3400456	0.6411668	3.650	0.000273 ***
RanchID30	2.4786038	0.6354871	3.900	0.000101 ***
RanchID31	0.0182450	0.1940761	0.094	0.925116
RanchID32	0.4886474	1.3332918	0.366	0.714053
RanchID33	2.9089436	0.6807773	4.273	2.07e-05 ***
RanchID34	0.0322724	0.4502099	0.072	0.942865
RanchID35	0.5634498	0.9687143	0.582	0.560903
RanchID36	2.9755583	0.6348515	4.687	3.06e-06 ***
RanchID37	2.5179606	0.6894491	3.652	0.000270 ***

Signif. codes: 0 ‘***’ 0.001 ‘**’ 0.01 ‘*’ 0.05 ‘.’ 0.1 ‘ ’ 1

Approximate significance of smooth terms:

	edf	Ref.df	F	p-value
s(hectares)	2.426412	3.100	3.132	0.02431 *
s(Year)	4.358754	11.000	25.580	< 2e-16 ***
s(long,lat):Year1997	2.856415	3.045	1.299	0.24935
s(long,lat):Year1998	2.000272	2.001	9.663	6.89e-05 ***
s(long,lat):Year1999	2.000476	2.001	6.651	0.00133 **
s(long,lat):Year2000	2.679975	2.941	0.576	0.58128
s(long,lat):Year2001	2.000167	2.000	1.088	0.33715
s(long,lat):Year2002	2.000115	2.000	2.283	0.10241
s(long,lat):Year2003	8.202535	8.558	9.037	< 2e-16 ***
s(long,lat):Year2004	8.208758	8.802	41.172	< 2e-16 ***
s(long,lat):Year2005	5.839791	6.983	2.156	0.03427 *
s(long,lat):Year2006	2.000117	2.000	0.152	0.85944
s(long,lat):Year2007	6.775072	7.773	45.096	< 2e-16 ***
s(long,lat):Year2008	7.288043	7.944	25.966	< 2e-16 ***
s(FieldID)	0.007352	512.000	0.000	0.77661

Signif. codes: 0 '***' 0.001 '**' 0.01 '*' 0.05 '.' 0.1 ' ' 1

R-sq.(adj) =   0.8   Deviance explained = 81.8%
-REML = 869.47 Scale est. = 0.16182   n = 1464

5. Effects on insecticides targeting *Aphis gossypii*

> summary(*SpodopteraInsecticides*)

Family: gaussian
Link function: identity

Formula:
AphisInsecticides ~ s(FieldSize) + CottonSpecies + RotationCrop + AlfalfaMatrix + AlmondsMatrix + BarleyMatrix + CornMatrix + CottonMatrix + BeansMatrix + GarlicMatrix + GrapesMatrix + LettuceMatrix + MelonsMatrix + OnionsMatrix + PistachiosMatrix + PotatoesMatrix + SafflowerMatrix + SugarbeetsMatrix + TomatoesMatrix + WheatMatrix + NaturalMatrix + FallowMatrix + s(*Year*, bs = "re") + *RanchID* + s(*FieldID,* bs = "re") + s(*Longitude, Latitude*, bs = "tp", by = *Year,* k = 10), method = "REML", data = *CottonFieldSize*

Family: gaussian
Link function: identity

Parametric coefficients:

	Estimate	Std. Error	t value	Pr(>|t|)
(Intercept)	0.390856	0.690247	0.566	0.571315
CottonSpeciesPima	-0.010693	0.051121	-0.209	0.834346
CottonSpeciesunknown	-0.149994	0.121721	-1.232	0.218061
RotationCropBarley	-0.064913	0.305013	-0.213	0.831501
RotationCropCarrots	0.037898	0.310392	0.122	0.902841
RotationCropCorn	-0.174766	0.330860	-0.528	0.597438
RotationCropCotton	-0.171054	0.135122	-1.266	0.205761
RotationCropBeans	-0.328031	0.207110	-1.584	0.113465
RotationCropGarlic	0.074037	0.169490	0.437	0.662311
RotationCropLettuce	2.088948	0.614788	3.398	0.000699 ***
RotationCropMelons	1.504126	0.437203	3.440	0.000599 ***
RotationCropOnions	0.248531	0.205980	1.207	0.227810
RotationCropPeppers	0.318157	0.636129	0.500	0.617055
RotationCropPotatoes	0.170438	0.171695	0.993	0.321047
RotationCropSafflower	-0.441359	0.217542	-2.029	0.042673 *

RotationCropSugarbeets	-0.263421	0.218007	-1.208	0.227142
RotationCropTomatoes	-0.093652	0.150889	-0.621	0.534923
RotationCropunknown	-0.048785	0.146554	-0.333	0.739277
RotationCropWheat	-0.056176	0.140898	-0.399	0.690178
AlfalfaMatrix	-0.313751	0.230877	-1.359	0.174391
AlmondsMatrix	0.170249	0.356496	0.478	0.633040
BarleyMatrix	2.474455	0.652048	3.795	0.000154 ***
CornMatrix	-0.573328	0.511948	-1.120	0.262960
CottonMatrix	0.237689	0.109157	2.178	0.029618 *
BeansMatrix	-0.180798	0.450400	-0.401	0.688177
GarlicMatrix	-0.339611	0.371013	-0.915	0.360167
GrapesMatrix	-0.159334	0.772705	-0.206	0.836664
LettuceMatrix	0.428046	0.822211	0.521	0.602729
MelonsMatrix	0.957688	0.730863	1.310	0.190303
OnionsMatrix	0.332149	0.386414	0.860	0.390182
PistachiosMatrix	-0.001954	0.442355	-0.004	0.996476
PotatoesMatrix	0.217564	0.363718	0.598	0.549830
SafflowerMatrix	-0.648426	0.497857	-1.302	0.192993
SugarbeetsMatrix	-0.207365	0.653792	-0.317	0.751162
TomatoesMatrix	0.052225	0.202754	0.258	0.796771
WheatMatrix	-0.007123	0.160405	-0.044	0.964589
NaturalMatrix	0.213098	0.323971	0.658	0.510799
FallowMatrix	-0.357130	0.363367	-0.983	0.325866
RanchID1	1.002715	2.127194	0.471	0.637447
RanchID2	-0.511145	0.460718	-1.109	0.267435
RanchID3	1.245539	0.985585	1.264	0.206539
RanchID4	0.460890	0.385674	1.195	0.232290
RanchID5	-0.030077	0.234411	-0.128	0.897922
RanchID6	0.043654	0.190582	0.229	0.818861
RanchID7	0.866108	1.938625	0.447	0.655118
RanchID8	0.874870	1.962404	0.446	0.655803
RanchID9	-0.153903	0.217662	-0.707	0.479645
RanchID10	1.067247	1.336067	0.799	0.424550
RanchID11	-0.121730	0.255285	-0.477	0.633553
RanchID12	0.762650	2.040408	0.374	0.708633
RanchID13	0.906176	2.019325	0.449	0.653684
RanchID14	-0.158039	0.223985	-0.706	0.480574
RanchID15	1.060359	1.356696	0.782	0.434604
RanchID16	1.431858	0.924862	1.548	0.121815
RanchID17	1.809298	0.869356	2.081	0.037607 *
RanchID18	-0.111916	0.254861	-0.439	0.660642
RanchID19	1.288325	0.836903	1.539	0.123945
RanchID20	-0.093763	0.182833	-0.513	0.608152
RanchID21	1.525896	0.916120	1.666	0.096027 .

RanchID22	1.005480	0.886646	1.134	0.256988
RanchID23	0.689140	1.988742	0.347	0.729006
RanchID24	0.759964	0.825836	0.920	0.357616
RanchID25	-0.084356	0.229813	-0.367	0.713630
RanchID26	1.650780	0.967017	1.707	0.088039 .
RanchID27	-0.065676	0.304446	-0.216	0.829236
RanchID28	1.817101	0.901642	2.015	0.044071 *
RanchID29	1.169585	0.886936	1.319	0.187502
RanchID30	1.874137	0.881122	2.127	0.033604 *
RanchID31	0.257030	0.267047	0.962	0.335978
RanchID32	1.544304	1.849390	0.835	0.403848
RanchID33	2.027472	0.943462	2.149	0.031816 *
RanchID34	-0.979413	0.627789	-1.560	0.118974
RanchID35	1.398447	1.347715	1.038	0.299624
RanchID36	2.027808	0.877007	2.312	0.020919 *
RanchID37	1.277960	0.956424	1.336	0.181717

Signif. codes: 0 ‘***’ 0.001 ‘**’ 0.01 ‘*’ 0.05 ‘.’ 0.1 ‘ ’ 1

Approximate significance of smooth terms:

	edf	Ref.df	F	p-value
s(FieldSize)	2.9531	3.726	1.307	0.25249
s(Year)	4.5412	11.000	30.441	< 2e-16 ***
s(long,lat):Year1997	2.7639	2.982	5.409	0.00078 ***
s(long,lat):Year1998	2.8986	3.139	2.011	0.10064
s(long,lat):Year1999	2.0002	2.000	10.910	2.05e-05 ***
s(long,lat):Year2000	2.0001	2.000	4.563	0.01059 *
s(long,lat):Year2001	2.0003	2.001	1.229	0.29310
s(long,lat):Year2002	2.8308	3.048	1.441	0.24813
s(long,lat):Year2003	7.6780	8.259	4.139	3.04e-05 ***
s(long,lat):Year2004	8.2270	8.827	10.445	< 2e-16 ***
s(long,lat):Year2005	8.3709	8.879	14.973	< 2e-16 ***
s(long,lat):Year2006	2.0004	2.001	2.607	0.07406 .
s(long,lat):Year2007	2.4914	2.834	0.539	0.56778
s(long,lat):Year2008	5.3839	6.285	6.594	8.38e-07 ***
s(FieldID)	0.0122	512.000	0.000	0.51579

Signif. codes: 0 ‘***’ 0.001 ‘**’ 0.01 ‘*’ 0.05 ‘.’ 0.1 ‘ ’ 1

R-sq.(adj) = 0.678   Deviance explained = 70.7%
-REML = 1327.8 Scale est. = 0.31964   n = 1464

6. Effects on cotton yield

> summary(*CottonYield*)

Family: gaussian
Link function: identity

Formula:
CottonYield ~ s(FieldSize) + CottonSpecies + RotationCrop + AlfalfaMatrix + AlmondsMatrix + BarleyMatrix + CornMatrix + CottonMatrix + BeansMatrix + GarlicMatrix + GrapesMatrix + LettuceMatrix + MelonsMatrix + OnionsMatrix + PistachiosMatrix + PotatoesMatrix + SafflowerMatrix + SugarbeetsMatrix + TomatoesMatrix + WheatMatrix + NaturalMatrix + FallowMatrix + s(*Year*, bs = "re") + *RanchID* + s(*FieldID,* bs = "re") + s(*Longitude, Latitude*, bs = "tp", by = *Year,* k = 10), method = "REML", data = *CottonFieldSize*

Family: gaussian
Link function: identity

Parametric coefficients:

	Estimate	Std. Error	t value	Pr(>|t|)
(Intercept)	1690.110	308.639	5.476	5.64e-08 ***
CottonSpeciesPima	-138.892	19.305	-7.195	1.32e-12 ***
CottonSpeciesunknown	-183.570	77.618	-2.365	0.018239 *
RotationCropBarley	-47.999	114.254	-0.420	0.674507
RotationCropCarrots	147.660	113.852	1.297	0.194980
RotationCropCorn	254.756	164.053	1.553	0.120800
RotationCropCotton	67.830	54.228	1.251	0.211318
RotationCropBeans	177.099	82.067	2.158	0.031193 *
RotationCropGarlic	166.943	65.504	2.549	0.010980 *
RotationCropLettuce	255.378	228.046	1.120	0.263076
RotationCropMelons	312.376	157.512	1.983	0.047648 *
RotationCropOnions	120.597	84.173	1.433	0.152282
RotationCropPotatoes	117.193	71.983	1.628	0.103862
RotationCropSafflower	-51.796	82.574	-0.627	0.530642
RotationCropSugarbeets	-25.208	80.342	-0.314	0.753773
RotationCropTomatoes	103.367	59.493	1.737	0.082645 .
RotationCropunknown	28.687	60.017	0.478	0.632783
RotationCropWheat	81.310	56.375	1.442	0.149560
AlfalfaMatrix	226.075	102.796	2.199	0.028112 *
AlmondsMatrix	207.916	159.580	1.303	0.192944
BarleyMatrix	38.751	242.591	0.160	0.873122
CornMatrix	448.177	235.393	1.904	0.057234 .
CottonMatrix	1.679	46.781	0.036	0.971385

BeansMatrix	-34.592	168.936	-0.205	0.837802
GarlicMatrix	-7.587	142.511	-0.053	0.957553
GrapesMatrix	-279.159	330.056	-0.846	0.397892
LettuceMatrix	-277.252	306.375	-0.905	0.365737
MelonsMatrix	161.440	276.134	0.585	0.558935
OnionsMatrix	-122.968	145.634	-0.844	0.398690
PistachiosMatrix	-143.956	180.207	-0.799	0.424595
PotatoesMatrix	-488.896	148.533	-3.292	0.001035 **
SafflowerMatrix	-269.044	185.896	-1.447	0.148165
SugarbeetsMatrix	76.660	266.717	0.287	0.773856
TomatoesMatrix	-104.302	79.007	-1.320	0.187119
WheatMatrix	-39.025	64.973	-0.601	0.548233
NaturalMatrix	-83.494	149.090	-0.560	0.575604
FallowMatrix	-543.008	161.260	-3.367	0.000791 ***
RanchID1	-705.917	1094.330	-0.645	0.519047
RanchID2	194.866	232.240	0.839	0.401650
RanchID3	254.944	501.314	0.509	0.611190
RanchID4	-261.382	178.810	-1.462	0.144149
RanchID5	-106.765	120.561	-0.886	0.376090
RanchID6	-233.319	105.774	-2.206	0.027647 *
RanchID8	-349.406	1009.301	-0.346	0.729284
RanchID9	-102.992	123.812	-0.832	0.405721
RanchID10	100.577	669.412	0.150	0.880603
RanchID11	-146.775	122.619	-1.197	0.231621
RanchID12	-629.351	1051.926	-0.598	0.549800
RanchID14	-163.337	112.140	-1.457	0.145589
RanchID15	93.402	681.394	0.137	0.891002
RanchID16	261.319	472.028	0.554	0.579983
RanchID17	57.327	440.492	0.130	0.896482
RanchID18	-137.082	124.917	-1.097	0.272768
RanchID19	33.829	419.663	0.081	0.935771
RanchID20	-57.446	98.111	-0.586	0.558341
RanchID21	166.873	469.593	0.355	0.722406
RanchID22	115.396	441.465	0.261	0.793849
RanchID23	-425.822	1020.386	-0.417	0.676547
RanchID24	-304.097	433.574	-0.701	0.483251
RanchID25	-29.727	117.087	-0.254	0.799641
RanchID26	198.887	504.633	0.394	0.693584
RanchID27	182.255	137.492	1.326	0.185319
RanchID28	216.753	458.614	0.473	0.636594
RanchID29	70.219	450.204	0.156	0.876090
RanchID30	142.995	458.661	0.312	0.755291
RanchID31	-55.900	135.540	-0.412	0.680126
RanchID33	165.245	474.704	0.348	0.727845

RanchID34	51.564	322.919	0.160	0.873169
RanchID35	-221.892	674.258	-0.329	0.742163
RanchID36	318.113	441.585	0.720	0.471472
RanchID37	189.938	482.618	0.394	0.694000

Signif. codes: 0 ‘***’ 0.001 ‘**’ 0.01 ‘*’ 0.05 ‘.’ 0.1 ‘ ’ 1

Approximate significance of smooth terms:

	edf	Ref.df	F	p-value
s(hectares)	1.001	1.001	1.502	0.220915
s(Year)	4.418	10.000	135.610	< 2e-16 ***
s(long,lat):Year1998	2.244	2.419	3.644	0.034210 *
s(long,lat):Year1999	2.000	2.000	1.375	0.253431
s(long,lat):Year2000	2.000	2.000	1.159	0.314354
s(long,lat):Year2001	2.000	2.000	2.756	0.064035 .
s(long,lat):Year2002	2.000	2.000	1.213	0.297747
s(long,lat):Year2003	7.650	7.903	12.055	< 2e-16 ***
s(long,lat):Year2004	7.596	8.379	3.345	0.000459 ***
s(long,lat):Year2005	4.591	5.441	1.065	0.350774
s(long,lat):Year2006	2.014	2.019	0.148	0.867684
s(long,lat):Year2007	5.521	6.405	3.009	0.005159 **
s(long,lat):Year2008	5.639	6.439	8.906	< 2e-16 ***
s(FieldID)	213.681	450.000	0.935	< 2e-16 ***

Signif. codes: 0 ‘***’ 0.001 ‘**’ 0.01 ‘*’ 0.05 ‘.’ 0.1 ‘ ’ 1

R-sq.(adj) = 0.772   Deviance explained = 83.4%
-REML = 7864.7 Scale est. = 32807   n = 1236

Table S8. Effects of field size on citrus pest densities, targeted pesticide applications, and yield. Descriptions of GAM modeling results.

1. Effects on *Scirtothrips citri* densities

> summary(*ScirtothripsDensity*)

Family: gaussian
Link function: identity

Formula:
gam(*ScirtothripsDensity* ~ s(*FieldSize*) + *CitrusSpecies* + *PlantingAge* + *CitrusMatrix* + *NaturalMatrix* + s(*Year*, bs = "re") + *RanchID* + s(*FieldID*, bs = "re") + s(*Longitude*, *Latitude*, bs = "tp", by = *Year*, k = 10), method = "REML", data = *CitrusFieldSize*)

Parametric coefficients:

	Estimate	Std. Error	t value	Pr(>|t|)
(Intercept)	3.745e-02	3.072e-02	1.219	0.22293
CitrusMatrix	3.547e-03	6.652e-03	0.533	0.59395
NaturalMatrix	-3.740e-03	9.013e-03	-0.415	0.67822
CitrusSpecies-clementina	-7.041e-03	4.684e-03	-1.503	0.13294
CitrusSpecies-clementina x sinensis	-3.980e-02	7.691e-03	-5.175	2.51e-07 ***
CitrusSpecies-limettioides	-1.024e-02	9.477e-03	-1.081	0.27987
CitrusSpecies-limon	-4.274e-03	8.562e-03	-0.499	0.61773
CitrusSpecies-maxima	-2.839e-03	9.031e-03	-0.314	0.75328
CitrusSpecies-meyeri	-1.581e-02	9.505e-03	-1.663	0.09650 .
CitrusSpecies-paradisi	-4.022e-03	1.145e-02	-0.351	0.72548
CitrusSpecies-reticulata	-1.039e-02	4.109e-03	-2.528	0.01156 *
CitrusSpecies-sinensis	4.628e-03	4.023e-03	1.151	0.25005
CitrusSpecies-tangelo	5.283e-03	5.647e-03	0.936	0.34958
CitrusSpecies-unshiu	9.472e-03	5.302e-03	1.787	0.07417 .
PlantingAge	-7.327e-05	5.554e-05	-1.319	0.18721
RanchID1	2.658e-04	7.005e-03	0.038	0.96974
RanchID2	1.033e-02	2.504e-02	0.413	0.67991
RanchID3	-1.502e-02	2.021e-02	-0.743	0.45748
RanchID4	-2.397e-03	1.659e-02	-0.144	0.88512
RanchID5	5.302e-03	1.132e-02	0.468	0.63968
RanchID6	-5.329e-03	1.284e-02	-0.415	0.67819
RanchID7	-1.085e-02	1.136e-02	-0.955	0.33963
RanchID8	-4.621e-03	1.335e-02	-0.346	0.72923
RanchID9	-1.128e-02	1.519e-02	-0.743	0.45770

RanchID10	3.896e-03	1.252e-02	0.311	0.75569
RanchID11	-7.789e-03	1.181e-02	-0.659	0.50972
RanchID12	1.059e-02	1.691e-02	0.626	0.53143
RanchID13	1.291e-03	1.161e-02	0.111	0.91148
RanchID14	-6.196e-02	6.247e-02	-0.992	0.32135
RanchID15	-5.073e-02	4.807e-02	-1.055	0.29141
RanchID16	-4.823e-02	4.784e-02	-1.008	0.31348
RanchID17	8.091e-03	1.489e-02	0.543	0.58687
RanchID18	-1.074e-02	1.537e-02	-0.699	0.48464
RanchID19	-1.687e-02	1.804e-02	-0.935	0.34996
RanchID20	-1.162e-02	1.419e-02	-0.819	0.41294
RanchID21	-1.336e-02	1.418e-02	-0.943	0.34595
RanchID22	-1.743e-02	2.330e-02	-0.748	0.45446
RanchID23	-1.094e-02	4.042e-02	-0.271	0.78668
RanchID24	-2.137e-02	4.826e-02	-0.443	0.65791
RanchID25	-9.325e-03	4.077e-02	-0.229	0.81912
RanchID26	-3.019e-02	4.880e-02	-0.619	0.53620
RanchID27	5.668e-03	4.216e-02	0.134	0.89308
RanchID28	-1.537e-02	4.176e-02	-0.368	0.71292
RanchID29	-2.329e-02	4.610e-02	-0.505	0.61342
RanchID30	-1.802e-02	3.003e-02	-0.600	0.54843
RanchID31	2.036e-02	4.315e-02	0.472	0.63711
RanchID32	-2.800e-02	4.406e-02	-0.636	0.52511
RanchID33	-7.155e-03	4.421e-02	-0.162	0.87145
RanchID34	1.260e-02	3.888e-02	0.324	0.74582
RanchID35	-2.274e-02	3.187e-02	-0.714	0.47559
RanchID36	-1.684e-02	4.416e-02	-0.381	0.70302
RanchID37	-3.534e-02	4.605e-02	-0.767	0.44298
RanchID38	-6.207e-03	4.150e-02	-0.150	0.88114
RanchID39	-4.314e-02	5.399e-02	-0.799	0.42440
RanchID40	-2.606e-02	4.754e-02	-0.548	0.58369
RanchID41	5.882e-03	1.850e-02	0.318	0.75052
RanchID42	-7.291e-03	3.626e-02	-0.201	0.84067
RanchID43	-1.114e-02	2.867e-02	-0.389	0.69757
RanchID44	-4.194e-02	7.261e-02	-0.578	0.56361
RanchID45	-3.362e-03	3.174e-02	-0.106	0.91566
RanchID46	2.486e-02	2.076e-02	1.198	0.23124
RanchID47	-1.824e-02	3.567e-02	-0.511	0.60918
RanchID48	1.104e-02	1.501e-02	0.736	0.46191
RanchID49	1.123e-02	3.798e-02	0.296	0.76756
RanchID50	-1.047e-02	3.246e-02	-0.322	0.74713
RanchID51	-1.397e-02	2.879e-02	-0.485	0.62748
RanchID52	-8.932e-03	3.959e-02	-0.226	0.82152
RanchID53	-4.116e-03	2.892e-02	-0.142	0.88681

RanchID54	-1.619e-02	4.138e-02	-0.391	0.69558
RanchID55	1.094e-03	4.066e-02	0.027	0.97853
RanchID56	1.585e-02	4.580e-02	0.346	0.72940
RanchID57	-1.473e-02	3.877e-02	-0.380	0.70400
RanchID58	-1.031e-02	3.860e-02	-0.267	0.78941
RanchID59	1.047e-02	4.219e-02	0.248	0.80403
RanchID60	1.753e-02	3.412e-02	0.514	0.60749
RanchID62	-3.947e-02	3.952e-02	-0.999	0.31795
RanchID63	2.254e-02	1.646e-02	1.369	0.17113
RanchID64	-1.550e-02	4.100e-02	-0.378	0.70550
RanchID65	-1.223e-02	3.080e-02	-0.397	0.69132
RanchID66	-1.178e-02	3.409e-02	-0.346	0.72966
RanchID67	-5.963e-03	3.924e-02	-0.152	0.87924
RanchID68	4.121e-02	1.414e-02	2.914	0.00361 **
RanchID69	-1.936e-02	4.716e-02	-0.410	0.68155
RanchID70	6.639e-03	2.086e-02	0.318	0.75032
RanchID71	-2.846e-02	4.788e-02	-0.594	0.55228
RanchID72	-3.130e-02	5.376e-02	-0.582	0.56045
RanchID73	-1.395e-02	3.843e-02	-0.363	0.71662
RanchID74	3.619e-02	4.060e-02	0.891	0.37285
RanchID75	-6.211e-03	3.024e-02	-0.205	0.83729
RanchID76	-1.125e-02	4.585e-02	-0.245	0.80623
RanchID77	-2.564e-02	4.645e-02	-0.552	0.58098
RanchID78	-2.341e-02	4.591e-02	-0.510	0.61023
RanchID79	-1.142e-02	4.318e-02	-0.264	0.79151
RanchID80	-1.551e-02	4.254e-02	-0.365	0.71539
RanchID81	-1.643e-02	4.422e-02	-0.371	0.71031
RanchID82	-3.601e-02	7.116e-02	-0.506	0.61291
RanchID83	-1.631e-02	4.679e-02	-0.349	0.72737

Signif. codes: 0 '***' 0.001 '**' 0.01 '*' 0.05 '.' 0.1 ' ' 1

Approximate significance of smooth terms:

	edf	Ref.df	F	p-value
s(FieldSize)	1.003	1.005	6.093	0.013462 *
s(Year)	12.659	15.000	17.586	< 2e-16 ***
s(Longitude, Latitude):Year2003	2.001	2.002	0.640	0.527242
s(Longitude, Latitude):Year2004	2.002	2.003	0.575	0.562934
s(Longitude, Latitude):Year2005	2.003	2.005	1.179	0.308477
s(Longitude, Latitude):Year2006	2.000	2.001	0.611	0.542903
s(Longitude, Latitude):Year2007	6.096	7.340	1.898	0.059017 .
s(Longitude, Latitude):Year2008	4.992	6.294	1.058	0.384814
s(Longitude, Latitude):Year2009	5.652	6.936	1.333	0.278235
s(Longitude, Latitude):Year2010	2.001	2.001	0.600	0.548731

s(Longitude, Latitude):Year2011	5.478	6.732	1.633	0.138392
s(Longitude, Latitude):Year2012	3.583	4.524	0.471	0.706023
s(Longitude, Latitude):Year2013	5.268	6.439	1.242	0.257592
s(Longitude, Latitude):Year2014	6.067	7.091	1.756	0.092613 .
s(Longitude, Latitude):Year2015	6.677	7.617	3.912	0.000297 ***
s(Longitude, Latitude):Year2016	7.127	7.975	6.076	< 2e-16 ***
s(Longitude, Latitude):Year2017	3.611	4.547	0.437	0.826419
s(Longitude, Latitude):Year2018	2.002	2.003	0.564	0.568802
s(FieldID)	25.599	198.000	0.155	0.055923 .

Signif. codes: 0 '***' 0.001 '**' 0.01 '*' 0.05 '.' 0.1 ' ' 1

R-sq.(adj) = 0.353   Deviance explained = 41.2%
-REML = -4869.2 Scale est. = 0.00041313 n = 2205

2. Effects on insecticides targeting *Scirtothrips citri*

> summary(*ScirtothripsInsecticides*)

Family: gaussian
Link function: identity

Formula:
gam(*ScirtothripsInsecticides* ~ s(*FieldSize*) + *CitrusSpecies* + *PlantingAge* + *CitrusMatrix* + *NaturalMatrix* + s(*Year*, bs = "re") + *RanchID* + s(*FieldID*, bs = "re") + s(*Longitude, Latitude*, bs = "tp", by = *Year*, k = 10), method = "REML", data = *CitrusFieldSize*)

Parametric coefficients:

	Estimate	Std. Error	t value	Pr(>|t|)
(Intercept)	0.871336	0.888393	0.981	0.3268
CitrusMatrix	0.304316	0.194058	1.568	0.1170
NaturalMatrix	0.340464	0.239755	1.420	0.1558
CitrusSpecies-clementina	-0.147201	0.132572	-1.110	0.2670
CitrusSpecies-clementina x sinensis	-0.834183	0.181391	-4.599	4.52e-06 ***
CitrusSpecies-limettioides	-0.540419	0.344771	-1.567	0.1172
CitrusSpecies-limon	-0.325451	0.248247	-1.311	0.1900
CitrusSpecies-maxima	-0.254937	0.225594	-1.130	0.2586
CitrusSpecies-meyeri	-0.794375	0.345810	-2.297	0.0217 *
CitrusSpecies-reticulata	-0.303304	0.126340	-2.401	0.0165 *
CitrusSpecies-sinensis	0.034429	0.122043	0.282	0.7779
CitrusSpecies-tangelo	0.041453	0.188172	0.220	0.8257
CitrusSpecies-unshiu	-0.154362	0.155062	-0.995	0.3196

PlantingAge	0.002463	0.001398	1.762	0.0782 .
RanchID1	0.170944	0.176299	0.970	0.3324
RanchID2	-0.585160	0.590866	-0.990	0.3221
RanchID5	0.045953	0.428972	0.107	0.9147
RanchID6	-0.066769	0.391900	-0.170	0.8647
RanchID8	-0.037372	0.467720	-0.080	0.9363
RanchID9	0.197804	0.431088	0.459	0.6464
RanchID11	-0.413781	0.357738	-1.157	0.2475
RanchID12	-0.079821	0.536992	-0.149	0.8818
RanchID13	0.064301	0.274334	0.234	0.8147
RanchID14	-1.274863	1.633020	-0.781	0.4351
RanchID15	-0.957460	1.243652	-0.770	0.4415
RanchID16	-0.625105	1.242126	-0.503	0.6148
RanchID17	-0.389554	0.375457	-1.038	0.2996
RanchID19	0.325891	0.473092	0.689	0.4910
RanchID20	-0.228397	0.392408	-0.582	0.5606
RanchID21	0.053987	0.359425	0.150	0.8806
RanchID22	0.100462	0.621568	0.162	0.8716
RanchID23	-1.208627	1.064481	-1.135	0.2563
RanchID24	-0.999900	1.280736	-0.781	0.4351
RanchID25	-1.041890	1.080811	-0.964	0.3352
RanchID26	-0.553750	1.261857	-0.439	0.6608
RanchID27	-0.562170	1.027325	-0.547	0.5843
RanchID29	0.145062	1.163413	0.125	0.9008
RanchID30	-0.145243	0.794305	-0.183	0.8549
RanchID31	-0.582141	1.036854	-0.561	0.5746
RanchID32	-0.341455	1.132897	-0.301	0.7631
RanchID33	-1.123301	1.160241	-0.968	0.3331
RanchID34	-0.772118	0.939328	-0.822	0.4112
RanchID36	0.374227	1.084543	0.345	0.7301
RanchID37	-0.326755	1.179784	-0.277	0.7818
RanchID38	-0.888093	1.101384	-0.806	0.4201
RanchID39	-0.664808	1.402192	-0.474	0.6355
RanchID40	-0.031487	1.216791	-0.026	0.9794
RanchID41	0.186052	0.485823	0.383	0.7018
RanchID42	-0.823556	0.956316	-0.861	0.3892
RanchID43	-0.513472	0.757613	-0.678	0.4980
RanchID44	-0.256665	1.853345	-0.138	0.8899
RanchID45	-0.496264	0.822026	-0.604	0.5461
RanchID46	0.059311	0.550757	0.108	0.9143
RanchID47	-0.950915	0.948044	-1.003	0.3160
RanchID48	0.548428	0.388662	1.411	0.1584
RanchID49	-0.610848	0.919056	-0.665	0.5064
RanchID50	-0.127629	0.861966	-0.148	0.8823

RanchID51	-0.427417	0.762078	-0.561	0.5750
RanchID52	-1.079357	1.043407	-1.034	0.3010
RanchID53	-0.371971	0.767923	-0.484	0.6282
RanchID54	-1.938814	1.076359	-1.801	0.0718 .
RanchID55	-0.885197	0.984800	-0.899	0.3688
RanchID56	-0.808196	1.098457	-0.736	0.4620
RanchID57	-0.559588	1.031616	-0.542	0.5876
RanchID58	-1.057778	1.006314	-1.051	0.2933
RanchID59	-0.580585	1.019528	-0.569	0.5691
RanchID60	-0.354001	0.843497	-0.420	0.6748
RanchID61	-1.583820	1.497921	-1.057	0.2905
RanchID62	-2.193984	1.042584	-2.104	0.0355 *
RanchID63	0.216487	0.422179	0.513	0.6082
RanchID64	-1.021106	1.082354	-0.943	0.3456
RanchID65	-0.187298	0.807481	-0.232	0.8166
RanchID66	-0.760021	0.901222	-0.843	0.3991
RanchID67	-1.219734	1.012954	-1.204	0.2287
RanchID68	0.224813	0.359752	0.625	0.5321
RanchID69	-0.958766	1.254437	-0.764	0.4448
RanchID70	-0.355438	0.554396	-0.641	0.5215
RanchID71	-0.438022	1.227192	-0.357	0.7212
RanchID72	-0.539145	1.381476	-0.390	0.6964
RanchID73	-0.520045	1.014475	-0.513	0.6083
RanchID74	-0.860735	0.980694	-0.878	0.3802
RanchID75	-0.734733	0.799728	-0.919	0.3583
RanchID76	0.211957	1.127345	0.188	0.8509
RanchID77	0.236693	1.134878	0.209	0.8348
RanchID78	0.239518	1.134082	0.211	0.8328
RanchID79	0.142568	1.054880	0.135	0.8925
RanchID80	0.145194	1.044831	0.139	0.8895
RanchID81	0.217435	1.089585	0.200	0.8418
RanchID82	-0.405173	1.821537	-0.222	0.8240
RanchID83	-0.946633	1.243239	-0.761	0.4465

Signif. codes: 0 ‘***’ 0.001 ‘**’ 0.01 ‘*’ 0.05 ‘.’ 0.1 ‘ ’ 1

Approximate significance of smooth terms:

	edf	Ref.df	F	p-value
s(FieldSize)	1.000	1.001	0.133	0.715910
s(Year)	13.686	15.000	34.451	< 2e-16 ***
s(Longitude, Latitude):Year2003	2.000	2.001	2.295	0.100943
s(Longitude, Latitude):Year2004	2.000	2.001	1.386	0.250416
s(Longitude, Latitude):Year2005	2.005	2.009	1.170	0.311570
s(Longitude, Latitude):Year2006	5.527	6.604	1.955	0.184140

s(Longitude, Latitude):Year2007	2.001	2.002	0.475	0.622429
s(Longitude, Latitude):Year2008	6.173	7.504	2.915	0.004038 **
s(Longitude, Latitude):Year2009	6.864	8.070	3.444	0.000599 ***
s(Longitude, Latitude):Year2010	5.612	6.971	1.771	0.093894 .
s(Longitude, Latitude):Year2011	7.313	8.342	4.843	6.48e-06 ***
s(Longitude, Latitude):Year2012	2.216	2.394	0.813	0.459370
s(Longitude, Latitude):Year2013	7.221	8.176	2.711	0.004575 **
s(Longitude, Latitude):Year2014	8.529	8.918	14.665	< 2e-16 ***
s(Longitude, Latitude):Year2015	6.166	7.325	2.601	0.010198 *
s(Longitude, Latitude):Year2016	6.224	7.371	2.598	0.010124 *
s(Longitude, Latitude):Year2017	3.174	3.984	0.830	0.511458
s(Longitude, Latitude):Year2018	2.000	2.000	0.656	0.518952
s(FieldID)	7.890	162.000	0.052	0.273060

Signif. codes: 0 ‘***’ 0.001 ‘**’ 0.01 ‘*’ 0.05 ‘.’ 0.1 ‘ ’ 1

R-sq.(adj) = 0.486 Deviance explained = 53%
-REML = 1836.6 Scale est. = 0.26764 n = 2176

3. Effects on *Panonychus citri* densities

> summary(*PanonychusDensity*)

Family: gaussian
Link function: identity

Formula:
gam(*PanonychusDensity* ~ s(*FieldSize*) + *CitrusSpecies* + *PlantingAge* + *CitrusMatrix* + *NaturalMatrix* + s(*Year*, bs = "re") + *RanchID* + s(*FieldID*, bs = "re") + s(*Longitude*, *Latitude*, bs = "tp", by = *Year*, k = 10), method = "REML", data = *CitrusFieldSize*)

Parametric coefficients:

	Estimate	Std. Error	t value	Pr(>|t|)
(Intercept)	-0.0325256	0.2237658	-0.145	0.88445
CitrusMatrix	0.0678277	0.0722458	0.939	0.34800
NaturalMatrix	0.3011457	0.0977019	3.082	0.00210 **
CitrusSpecies-clementina	0.0975791	0.0720990	1.353	0.17618
CitrusSpecies-clementina x sinensis	0.1362332	0.0866863	1.572	0.11631
CitrusSpecies-reticulata	0.0694110	0.0694088	1.000	0.31750
CitrusSpecies-sinensis	0.1494219	0.0707209	2.113	0.03482 *
CitrusSpecies-tangelo	0.0578953	0.0827917	0.699	0.48451

CitrusSpecies-unshiu	0.0640876	0.0806232	0.795	0.42683
PlantingAge	-0.0005478	0.0005782	-0.948	0.34356
RanchID24	-0.1893659	0.1256983	-1.507	0.13220
RanchID25	-0.1782027	0.0816520	-2.182	0.02927 *
RanchID26	-0.0140258	0.2855123	-0.049	0.96083
RanchID27	0.2688442	0.3720086	0.723	0.47002
RanchID28	-0.2365237	0.1053240	-2.246	0.02491 *
RanchID29	-0.0167342	0.3457470	-0.048	0.96141
RanchID30	0.1329450	0.1356528	0.980	0.32726
RanchID31	0.2681748	0.4147684	0.647	0.51804
RanchID32	0.1042871	0.2855671	0.365	0.71503
RanchID33	-0.2353677	0.1029293	-2.287	0.02239 *
RanchID34	0.1446519	0.3741267	0.387	0.69909
RanchID35	0.2329961	0.1816293	1.283	0.19980
RanchID36	-0.1615045	0.4019876	-0.402	0.68793
RanchID37	-0.0121270	0.3071062	-0.039	0.96851
RanchID38	-0.0726560	0.0724900	-1.002	0.31640
RanchID39	-0.0756337	0.3038739	-0.249	0.80348
RanchID40	-0.1305199	0.3007831	-0.434	0.66441
RanchID41	0.1894528	0.2459397	0.770	0.44126
RanchID42	0.2522669	0.1084310	2.327	0.02016 *
RanchID43	0.0670908	0.1439564	0.466	0.64126
RanchID44	-0.3667331	0.4841774	-0.757	0.44894
RanchID45	0.1884298	0.1771198	1.064	0.28761
RanchID46	0.1375865	0.2227627	0.618	0.53693
RanchID47	-0.0156315	0.0894943	-0.175	0.86137
RanchID48	0.2065716	0.3032155	0.681	0.49583
RanchID49	0.2365892	0.3617784	0.654	0.51326
RanchID50	0.1658573	0.1165521	1.423	0.15499
RanchID51	0.2487234	0.1571208	1.583	0.11368
RanchID52	-0.2054883	0.0896333	-2.293	0.02205 *
RanchID53	0.0350476	0.1382828	0.253	0.79997
RanchID55	0.1995241	0.3695663	0.540	0.58937
RanchID56	0.1464418	0.4358026	0.336	0.73691
RanchID57	0.2248097	0.1021807	2.200	0.02799 *
RanchID58	-0.0382540	0.1242146	-0.308	0.75816
RanchID59	0.2140898	0.3868226	0.553	0.58005
RanchID60	0.0415532	0.3386374	0.123	0.90236
RanchID61	-0.3473639	0.2907995	-1.195	0.23251
RanchID62	-0.0606616	0.1761772	-0.344	0.73066
RanchID63	0.2157046	0.2862167	0.754	0.45121
RanchID64	0.0212802	0.0911233	0.234	0.81539
RanchID65	0.2483834	0.1464402	1.696	0.09012 .
RanchID66	0.1488238	0.0967809	1.538	0.12437

RanchID67	-0.3387677	0.1228528	-2.758	0.00591 **
RanchID68	0.2265837	0.2846015	0.796	0.42611
RanchID69	-0.2041798	0.1198951	-1.703	0.08883 .
RanchID70	0.1573118	0.2423198	0.649	0.51634
RanchID71	0.1116753	0.3062690	0.365	0.71545
RanchID72	-0.1584525	0.3380454	-0.469	0.63935
RanchID73	0.0079218	0.0953601	0.083	0.93381
RanchID74	0.2244199	0.4139974	0.542	0.58786
RanchID75	0.0086515	0.1342604	0.064	0.94863
RanchID76	-0.1968986	0.4047179	-0.487	0.62670
RanchID77	-0.1527682	0.4199756	-0.364	0.71610
RanchID78	-0.0628804	0.3925003	-0.160	0.87275
RanchID79	-0.0643424	0.4113682	-0.156	0.87574
RanchID80	-0.1008456	0.3960001	-0.255	0.79903
RanchID81	-0.1969482	0.3904752	-0.504	0.61409
RanchID82	-0.4128396	0.4663774	-0.885	0.37622
RanchID83	-0.2247809	0.1172733	-1.917	0.05551 .

Signif. codes: 0 ‘***’ 0.001 ‘**’ 0.01 ‘*’ 0.05 ‘.’ 0.1 ‘ ’ 1

Approximate significance of smooth terms:

	edf	Ref.df	F	p-value
s(FieldSize)	1.000	1.000	2.922	0.0876 .
s(Year)	9.584	10.000	44.822	<2e-16 ***
s(Longitude, Latitude):Year2007	3.262	3.938	0.769	0.5500
s(Longitude, Latitude):Year2008	4.635	5.625	1.600	0.1373
s(Longitude, Latitude):Year2009	4.568	5.674	1.038	0.5456
s(Longitude, Latitude):Year2010	4.423	5.410	0.954	0.4825
s(Longitude, Latitude):Year2011	7.168	8.198	2.102	0.0400 *
s(Longitude, Latitude):Year2012	8.083	8.685	8.817	<2e-16 ***
s(Longitude, Latitude):Year2013	2.001	2.002	0.765	0.4656
s(Longitude, Latitude):Year2014	2.768	3.296	0.837	0.4403
s(Longitude, Latitude):Year2015	7.953	8.692	9.642	<2e-16 ***
s(Longitude, Latitude):Year2016	4.643	5.859	0.976	0.4439
s(Longitude, Latitude):Year2017	6.293	7.458	2.209	0.0286 *
s(FieldID)	9.702	124.000	0.089	0.1725

Signif. codes: 0 ‘***’ 0.001 ‘**’ 0.01 ‘*’ 0.05 ‘.’ 0.1 ‘ ’ 1

R-sq.(adj) = 0.529   Deviance explained = 57.9%
-REML = -343.33 Scale est. = 0.024483 n = 1350

4. Effects on insecticides targeting *Panonychus citri*

> summary(*PanonychusInsecticides*)

Family: gaussian
Link function: identity

Formula:
gam(*PanonychusInsecticides* ~ s(*FieldSize*) + *CitrusSpecies* + *PlantingAge* + *CitrusMatrix* + *NaturalMatrix* + s(*Year*, bs = "re") + *RanchID* + s(*FieldID*, bs = "re") + s(*Longitude*, *Latitude*, bs = "tp", by = *Year*, k = 10), method = "REML", data = *CitrusFieldSize*)

Parametric coefficients:

```
                                  Estimate   Std. Error  t value  Pr(>|t|)
(Intercept)                       0.5838867  0.9465130   0.617    0.53738
CitrusMatrix                      0.4233059  0.2057903   2.057    0.03982 *
NaturalMatrix                     -0.2873685 0.2339054   -1.229   0.21938
CitrusSpecies-clementina          -0.1137470 0.1327536   -0.857   0.39165
CitrusSpecies-clementina x sinensis -0.5548147 0.1812687 -3.061   0.00224 **
CitrusSpecies-limettioides        -0.0979522 0.3360160   -0.292   0.77069
CitrusSpecies-limon               -0.3253816 0.2544681   -1.279   0.20116
CitrusSpecies-maxima              -0.0618655 0.2304404   -0.268   0.78837
CitrusSpecies-meyeri              -0.0558350 0.3373728   -0.165   0.86857
CitrusSpecies-reticulata          -0.1999980 0.1264670   -1.581   0.11394
CitrusSpecies-sinensis            -0.0257330 0.1219514   -0.211   0.83290
CitrusSpecies-tangelo             0.0782277  0.1917202   0.408    0.68330
CitrusSpecies-unshiu              -0.1893566 0.1553473   -1.219   0.22302
PlantingAge                       0.0005391  0.0014358   0.375    0.70737
RanchID1                          0.3267793  0.1844312   1.772    0.07658 .
RanchID2                          0.2491184  0.6284140   0.396    0.69184
RanchID5                          -0.0780857 0.4123010   -0.189   0.84981
RanchID6                          -0.0157572 0.3865660   -0.041   0.96749
RanchID8                          0.1137745  0.4550959   0.250    0.80261
RanchID9                          -0.1720253 0.4361490   -0.394   0.69332
RanchID11                         -0.0952268 0.3543168   -0.269   0.78814
RanchID12                         0.3404248  0.5225987   0.651    0.51486
RanchID13                         0.1752580  0.2883071   0.608    0.54333
RanchID14                         -0.7355801 1.7452199   -0.421   0.67345
RanchID15                         -1.0518619 1.3286972   -0.792   0.42866
RanchID16                         -0.6770746 1.3270300   -0.510   0.60996
RanchID17                         0.2345042  0.4003193   0.586    0.55808
RanchID19                         -0.2738319 0.5042532   -0.543   0.58716
RanchID20                         -0.3836224 0.4037925   -0.950   0.34221
RanchID21                         -0.1952742 0.3819645   -0.511   0.60924
RanchID22                         -0.2215456 0.6633192   -0.334   0.73842
```

RanchID23	-0.0911094	1.1363109	-0.080	0.93610
RanchID24	-0.2083046	1.3688283	-0.152	0.87906
RanchID25	-0.1408024	1.1547955	-0.122	0.90297
RanchID26	-0.2031918	1.3470496	-0.151	0.88012
RanchID27	1.3882748	1.0957715	1.267	0.20533
RanchID29	-0.0965690	1.2423874	-0.078	0.93805
RanchID30	0.6270165	0.8500498	0.738	0.46083
RanchID31	1.3373785	1.1061944	1.209	0.22681
RanchID32	-0.3593700	1.2101867	-0.297	0.76653
RanchID33	0.0827932	1.2381493	0.067	0.94669
RanchID34	0.9047209	1.0008856	0.904	0.36615
RanchID36	-0.1301489	1.1581994	-0.112	0.91054
RanchID37	-0.4325378	1.2598563	-0.343	0.73139
RanchID38	0.0616432	1.1760301	0.052	0.95820
RanchID39	-0.1800201	1.4982763	-0.120	0.90438
RanchID40	-0.4064906	1.2999784	-0.313	0.75455
RanchID41	-0.0561900	0.5213623	-0.108	0.91418
RanchID42	0.3682637	1.0223239	0.360	0.71872
RanchID43	-0.0390932	0.8105853	-0.048	0.96154
RanchID44	-0.6674171	1.9806919	-0.337	0.73618
RanchID45	0.4150828	0.8770718	0.473	0.63608
RanchID46	0.0001033	0.5912840	0.000	0.99986
RanchID47	0.0401741	1.0129829	0.040	0.96837
RanchID48	0.1696407	0.4167167	0.407	0.68399
RanchID49	1.1360124	0.9799047	1.159	0.24647
RanchID50	0.4610101	0.9215290	0.500	0.61694
RanchID51	0.1333716	0.8154386	0.164	0.87010
RanchID52	-0.0293485	1.1135311	-0.026	0.97898
RanchID53	0.1388245	0.8222112	0.169	0.86594
RanchID54	0.2006047	1.1218349	0.179	0.85810
RanchID55	0.8628590	1.0508056	0.821	0.41167
RanchID56	1.2550793	1.1711914	1.072	0.28402
RanchID57	0.5111499	1.1009130	0.464	0.64249
RanchID58	0.3256614	1.0738070	0.303	0.76171
RanchID59	1.2895060	1.0872298	1.186	0.23575
RanchID60	1.0821944	0.8921082	1.213	0.22525
RanchID61	-0.1163044	1.5813787	-0.074	0.94138
RanchID62	-0.6983963	1.1011382	-0.634	0.52599
RanchID63	-0.0389699	0.4510136	-0.086	0.93115
RanchID64	-0.1333789	1.1547430	-0.116	0.90806
RanchID65	0.6180698	0.8625719	0.717	0.47374
RanchID66	0.4874758	0.9651987	0.505	0.61358
RanchID67	0.4129204	1.0800063	0.382	0.70226
RanchID68	0.2321593	0.3824265	0.607	0.54388

RanchID69	-0.0357014	1.3393734	-0.027	0.97874
RanchID70	-0.6038554	0.5941635	-1.016	0.30961
RanchID71	-0.2191167	1.3111301	-0.167	0.86729
RanchID72	-0.5846642	1.4756313	-0.396	0.69199
RanchID73	0.2552078	1.0839284	0.235	0.81389
RanchID74	0.9559645	1.0436990	0.916	0.35981
RanchID75	0.1152411	0.8559378	0.135	0.89291
RanchID76	-0.3144642	1.2039530	-0.261	0.79397
RanchID77	-0.1152258	1.2124446	-0.095	0.92430
RanchID78	-0.3074664	1.2116619	-0.254	0.79971
RanchID79	0.0348726	1.1269654	0.031	0.97532
RanchID80	-0.1472485	1.1158439	-0.132	0.89503
RanchID81	-0.3644706	1.1643031	-0.313	0.75429
RanchID82	-0.6653735	1.9466633	-0.342	0.73254
RanchID83	-0.1344051	1.3274201	-0.101	0.91936

Signif. codes: 0 '***' 0.001 '**' 0.01 '*' 0.05 '.' 0.1 ' ' 1

Approximate significance of smooth terms:

	edf	Ref.df	F	p-value
s(FieldSize)	1.000	1.000	0.011	0.916139
s(Year)	13.724	15.000	66.009	< 2e-16 ***
s(Longitude, Latitude):Year2003	2.000	2.000	0.232	0.793208
s(Longitude, Latitude):Year2004	2.001	2.003	0.148	0.862950
s(Longitude, Latitude):Year2005	2.001	2.002	0.086	0.918228
s(Longitude, Latitude):Year2006	2.003	2.005	0.099	0.906320
s(Longitude, Latitude):Year2007	5.210	6.325	1.638	0.148838
s(Longitude, Latitude):Year2008	6.761	7.933	1.766	0.061682 .
s(Longitude, Latitude):Year2009	5.962	7.236	1.541	0.137731
s(Longitude, Latitude):Year2010	4.645	5.818	0.533	0.797632
s(Longitude, Latitude):Year2011	5.479	6.729	1.868	0.084652 .
s(Longitude, Latitude):Year2012	6.075	7.252	1.703	0.099781 .
s(Longitude, Latitude):Year2013	8.172	8.743	5.734	9.83e-08 ***
s(Longitude, Latitude):Year2014	8.575	8.921	11.723	< 2e-16 ***
s(Longitude, Latitude):Year2015	7.283	8.147	7.158	< 2e-16 ***
s(Longitude, Latitude):Year2016	8.462	8.850	4.980	8.98e-07 ***
s(Longitude, Latitude):Year2017	6.626	7.643	1.329	0.303932
s(Longitude, Latitude):Year2018	2.000	2.000	0.251	0.778336
s(FieldID)	35.585	162.000	0.330	0.000291 ***

Signif. codes: 0 '***' 0.001 '**' 0.01 '*' 0.05 '.' 0.1 ' ' 1

R-sq.(adj) = 0.578   Deviance explained = 62.1%
-REML = 1720.4 Scale est. = 0.23081   n = 2176

5. Effects on *Scudderia furcata* densities

> summary(*ScudderiaDensity*)

Family: gaussian
Link function: identity

Formula:
gam(*ScudderiaDensity* ~ s(*FieldSize*) + *CitrusSpecies* + *PlantingAge* + *CitrusMatrix* + *NaturalMatrix* + s(*Year*, bs = "re") + *RanchID* + s(*FieldID*, bs = "re") + s(*Longitude*, *Latitude*, bs = "tp", by = *Year*, k = 10), method = "REML", data = *CitrusFieldSize*)

Parametric coefficients:

	Estimate	Std. Error	t value	Pr(>|t|)
(Intercept)	0.0934270	0.0552509	1.691	0.0913 .
CitrusMatrix	0.0246383	0.0180042	1.368	0.1716
NaturalMatrix	0.0253354	0.0225840	1.122	0.2623
CitrusSpecies-clementina	0.0099249	0.0159066	0.624	0.5329
CitrusSpecies-clementina x sinensis	-0.0423249	0.0226071	-1.872	0.0616 .
CitrusSpecies-paradisi	-0.0035387	0.0318945	-0.111	0.9117
CitrusSpecies-reticulata	-0.0144818	0.0149689	-0.967	0.3337
CitrusSpecies-sinensis	0.0171229	0.0151849	1.128	0.2599
CitrusSpecies-tangelo	0.0171138	0.0174723	0.979	0.3277
CitrusSpecies-unshiu	-0.0039618	0.0231471	-0.171	0.8642
PlantingAge	-0.0001541	0.0001757	-0.877	0.3809
RanchID23	-0.0656846	0.0715226	-0.918	0.3588
RanchID24	-0.0859194	0.0838994	-1.024	0.3062
RanchID25	-0.0647982	0.0702484	-0.922	0.3566
RanchID26	-0.1215263	0.0815274	-1.491	0.1365
RanchID27	0.0352826	0.1062582	0.332	0.7400
RanchID28	-0.0929454	0.0794550	-1.170	0.2425
RanchID29	-0.1263724	0.0751512	-1.682	0.0931 .
RanchID30	-0.0270223	0.0523257	-0.516	0.6057
RanchID31	0.0599256	0.1099582	0.545	0.5859
RanchID32	-0.1113697	0.0698905	-1.593	0.1115
RanchID33	-0.0543033	0.0808433	-0.672	0.5020
RanchID34	0.0065159	0.1045263	0.062	0.9503
RanchID36	-0.1294681	0.0741760	-1.745	0.0814 .
RanchID37	-0.1251896	0.0744132	-1.682	0.0930 .
RanchID38	-0.0591932	0.0734547	-0.806	0.4206
RanchID39	-0.1417983	0.0905751	-1.566	0.1179

RanchID40	-0.0973421	0.0770241	-1.264	0.2068
RanchID41	-0.0006914	0.0316529	-0.022	0.9826
RanchID42	-0.0603201	0.0629988	-0.957	0.3387
RanchID43	-0.0441359	0.0500855	-0.881	0.3785
RanchID44	-0.1204250	0.1307456	-0.921	0.3574
RanchID45	-0.0271019	0.0673369	-0.402	0.6875
RanchID46	-0.0187562	0.0329562	-0.569	0.5695
RanchID47	-0.0744618	0.0612379	-1.216	0.2244
RanchID48	-0.0151201	0.0260508	-0.580	0.5618
RanchID49	0.0365801	0.0971663	0.376	0.7067
RanchID50	-0.0479755	0.0567633	-0.845	0.3983
RanchID51	-0.0287294	0.0481857	-0.596	0.5512
RanchID52	-0.0702126	0.0715331	-0.982	0.3267
RanchID53	-0.0237353	0.0487713	-0.487	0.6267
RanchID55	0.0275688	0.1025502	0.269	0.7881
RanchID56	0.0442560	0.1163223	0.380	0.7037
RanchID57	-0.0711047	0.0646154	-1.100	0.2715
RanchID58	-0.0760794	0.0759494	-1.002	0.3168
RanchID59	0.0409296	0.1061253	0.386	0.6999
RanchID63	-0.0442844	0.0314147	-1.410	0.1591
RanchID64	-0.0009795	0.0731931	-0.013	0.9893
RanchID65	0.0110253	0.0595598	0.185	0.8532
RanchID66	-0.0757951	0.0609350	-1.244	0.2140
RanchID67	-0.0410447	0.0766264	-0.536	0.5924
RanchID68	0.0811114	0.0381435	2.126	0.0338 *
RanchID69	-0.0939883	0.0818057	-1.149	0.2510
RanchID70	-0.0303603	0.0415648	-0.730	0.4654
RanchID71	-0.1193224	0.0777991	-1.534	0.1256
RanchID72	-0.1380042	0.0905099	-1.525	0.1278
RanchID73	-0.0082281	0.0655272	-0.126	0.9001
RanchID74	0.0230622	0.1064999	0.217	0.8286
RanchID75	-0.0172503	0.0556537	-0.310	0.7567
RanchID76	-0.1295786	0.0769905	-1.683	0.0928 .
RanchID77	-0.1396971	0.0789697	-1.769	0.0774 .
RanchID78	-0.1370066	0.0771754	-1.775	0.0763 .
RanchID79	-0.1296118	0.0728018	-1.780	0.0755 .
RanchID80	-0.1294565	0.0708723	-1.827	0.0682 .
RanchID81	-0.1257946	0.0734756	-1.712	0.0874 .
RanchID82	-0.1808316	0.1274095	-1.419	0.1563
RanchID83	-0.0900335	0.0815684	-1.104	0.2701

Signif. codes: 0 ‘***’ 0.001 ‘**’ 0.01 ‘*’ 0.05 ‘.’ 0.1 ‘ ’ 1

Approximate significance of smooth terms:

	edf	Ref.df	F	p-value
s(FieldSize)	1.000	1.000	0.644	0.422694
s(Year)	6.813	10.000	4.140	< 2e-16 ***
s(Longitude, Latitude):Year2007	2.000	2.000	0.014	0.986127
s(Longitude, Latitude):Year2008	2.003	2.006	0.432	0.651173
s(Longitude, Latitude):Year2009	3.683	4.565	1.030	0.391259
s(Longitude, Latitude):Year2010	7.389	8.303	5.584	8.50e-07 ***
s(Longitude, Latitude):Year2011	2.000	2.000	0.605	0.546197
s(Longitude, Latitude):Year2012	3.005	3.752	0.378	0.840330
s(Longitude, Latitude):Year2013	6.520	7.569	3.006	0.003427 **
s(Longitude, Latitude):Year2014	5.901	7.059	3.562	0.000842 ***
s(Longitude, Latitude):Year2015	3.519	3.769	7.423	1.01e-05 ***
s(Longitude, Latitude):Year2016	2.000	2.000	0.944	0.389494
s(Longitude, Latitude):Year2017	3.091	3.832	0.552	0.669364
s(FieldID)	14.950	121.000	0.148	0.108531

Signif. codes: 0 ‘***’ 0.001 ‘**’ 0.01 ‘*’ 0.05 ‘.’ 0.1 ‘ ’ 1

R-sq.(adj) = 0.442   Deviance explained = 53.4%
-REML = -1353.8 Scale est. = 0.00081754 n = 792

6. Effects on insecticides targeting *Scudderia furcata*

> summary(*ScudderiaInsecticides*)

Family: gaussian
Link function: identity

Formula:
gam(*PanonychusInsecticides* ~ s(*FieldSize*) + *CitrusSpecies* + *PlantingAge* + *CitrusMatrix* + *NaturalMatrix* + s(*Year*, bs = "re") + *RanchID* + s(*FieldID*, bs = "re") + s(*Longitude*, *Latitude*, bs = "tp", by = *Year*, k = 10), method = "REML", data = *CitrusFieldSize*)

Parametric coefficients:

	Estimate	Std. Error	t value	Pr(>|t|)
(Intercept)	-0.5040713	0.5718283	-0.882	0.378150
CitrusMatrix	0.1553728	0.1358526	1.144	0.252892
NaturalMatrix	-0.0453579	0.1612037	-0.281	0.778456
CitrusSpecies-clementina	0.0305687	0.0869407	0.352	0.725172
CitrusSpecies-clementina x sinensis	-0.1897729	0.1193124	-1.591	0.111869
CitrusSpecies-limettioides	-0.0105785	0.2282683	-0.046	0.963042

CitrusSpecies-limon	-0.1284021	0.1621864	-0.792	0.428634
CitrusSpecies-maxima	-0.0031250	0.1471521	-0.021	0.983059
CitrusSpecies-meyeri	0.0274144	0.2291578	0.120	0.904788
CitrusSpecies-reticulata	0.0219514	0.0828565	0.265	0.791089
CitrusSpecies-sinensis	0.0262423	0.0800729	0.328	0.743150
CitrusSpecies-tangelo	0.2290254	0.1230674	1.861	0.062896 .
CitrusSpecies-unshiu	-0.0817985	0.1019152	-0.803	0.422295
PlantingAge	0.0002066	0.0009131	0.226	0.820971
RanchID1	0.1520033	0.1157337	1.313	0.189204
RanchID2	-0.2110038	0.3842112	-0.549	0.582939
RanchID5	0.0262058	0.2842597	0.092	0.926557
RanchID6	0.0376959	0.2582864	0.146	0.883979
RanchID8	0.5930243	0.3088317	1.920	0.054974 .
RanchID9	0.1473165	0.2846804	0.517	0.604879
RanchID11	0.7928616	0.2367008	3.350	0.000824 ***
RanchID12	0.0720602	0.3543193	0.203	0.838862
RanchID13	-0.0448277	0.1784092	-0.251	0.801636
RanchID14	0.9670375	1.0541432	0.917	0.359061
RanchID15	0.7010156	0.8024700	0.874	0.382457
RanchID16	0.7005643	0.8014693	0.874	0.382170
RanchID17	-0.1038270	0.2444595	-0.425	0.671086
RanchID19	0.4317954	0.3117204	1.385	0.166147
RanchID20	0.3604561	0.2573984	1.400	0.161555
RanchID21	0.3012008	0.2365859	1.273	0.203127
RanchID22	0.3684220	0.4050331	0.910	0.363139
RanchID23	0.6897259	0.6889679	1.001	0.316900
RanchID24	0.8854433	0.8272185	1.070	0.284576
RanchID25	0.7353102	0.6988766	1.052	0.292867
RanchID26	0.9962234	0.8143241	1.223	0.221334
RanchID27	0.0313505	0.6678839	0.047	0.962566
RanchID29	0.9554269	0.7504166	1.273	0.203098
RanchID30	0.6115378	0.5159123	1.185	0.236020
RanchID31	0.0937254	0.6739248	0.139	0.889406
RanchID32	0.9835176	0.7307379	1.346	0.178481
RanchID33	0.8398476	0.7505797	1.119	0.263305
RanchID34	0.2572185	0.6110565	0.421	0.673844
RanchID36	0.8990182	0.6997922	1.285	0.199050
RanchID37	0.7974810	0.7610622	1.048	0.294834
RanchID38	0.8975782	0.7120209	1.261	0.207599
RanchID39	1.2296883	0.9049401	1.359	0.174345
RanchID40	1.0103919	0.7847469	1.288	0.198057
RanchID41	0.6888010	0.3179673	2.166	0.030410 *
RanchID42	0.4554999	0.6197513	0.735	0.462443
RanchID43	0.5249041	0.4923795	1.066	0.286528

RanchID44	1.3158468	1.1956893	1.100	0.271251
RanchID45	0.5620993	0.5343461	1.052	0.292956
RanchID46	0.5420756	0.3598704	1.506	0.132147
RanchID47	0.5835979	0.6146262	0.950	0.342473
RanchID48	0.5199973	0.2546749	2.042	0.041303 *
RanchID49	0.2075404	0.5976829	0.347	0.728447
RanchID50	0.4005625	0.5593639	0.716	0.474012
RanchID51	0.3931447	0.4949473	0.794	0.427106
RanchID52	0.6890490	0.6755638	1.020	0.307871
RanchID53	0.4182226	0.4987844	0.838	0.401860
RanchID54	-0.0815216	0.7050609	-0.116	0.907963
RanchID55	0.1210242	0.6400854	0.189	0.850053
RanchID56	0.0071878	0.7140809	0.010	0.991970
RanchID57	0.7786796	0.6677443	1.166	0.243700
RanchID58	0.4668199	0.6526051	0.715	0.474497
RanchID59	0.1150956	0.6628844	0.174	0.862175
RanchID60	0.3673795	0.5493883	0.669	0.503761
RanchID61	0.2876650	0.9723877	0.296	0.767388
RanchID62	0.7666644	0.6779243	1.131	0.258234
RanchID63	0.5013665	0.2766533	1.812	0.070098 .
RanchID64	0.6434832	0.7004531	0.919	0.358381
RanchID65	0.4461044	0.5248871	0.850	0.395480
RanchID66	0.5500404	0.5842886	0.941	0.346622
RanchID67	0.5297537	0.6571216	0.806	0.420240
RanchID68	0.3383986	0.2363921	1.432	0.152441
RanchID69	0.9545356	0.8103672	1.178	0.238976
RanchID70	0.4856832	0.3641873	1.334	0.182485
RanchID71	1.0180683	0.7914774	1.286	0.198493
RanchID72	1.1260472	0.8909976	1.264	0.206449
RanchID73	0.4552950	0.6566107	0.693	0.488139
RanchID74	0.1537025	0.6368523	0.241	0.809311
RanchID75	0.5819316	0.5198333	1.119	0.263080
RanchID76	0.8798146	0.7272908	1.210	0.226532
RanchID77	0.8992185	0.7320768	1.228	0.219476
RanchID78	0.8697974	0.7314587	1.189	0.234532
RanchID79	0.8471819	0.6805840	1.245	0.213357
RanchID80	0.8808313	0.6742372	1.306	0.191564
RanchID81	0.9058806	0.7029233	1.289	0.197641
RanchID82	1.3649452	1.1752321	1.161	0.245608
RanchID83	1.0940968	0.8032752	1.362	0.173338

Signif. codes: 0 ‘***’ 0.001 ‘**’ 0.01 ‘*’ 0.05 ‘.’ 0.1 ‘ ’ 1

Approximate significance of smooth terms:

	edf	Ref.df	F	p-value
s(FieldSize)	1.000024	1.000	0.003	0.957451
s(Year)	13.392553	15.000	30.123	< 2e-16 ***
s(Longitude, Latitude):Year2003	2.000022	2.000	0.516	0.597196
s(Longitude, Latitude):Year2004	3.023232	3.690	0.624	0.669639
s(Longitude, Latitude):Year2005	2.000040	2.000	0.595	0.551647
s(Longitude, Latitude):Year2006	2.000031	2.000	0.582	0.558898
s(Longitude, Latitude):Year2007	6.593290	7.539	1.799	0.143251
s(Longitude, Latitude):Year2008	8.013375	8.743	6.463	< 2e-16 ***
s(Longitude, Latitude):Year2009	7.006390	8.161	2.573	0.008108 **
s(Longitude, Latitude):Year2010	8.087962	8.772	6.077	3.00e-07 ***
s(Longitude, Latitude):Year2011	7.345785	8.352	4.019	8.49e-05 ***
s(Longitude, Latitude):Year2012	2.017532	2.032	0.512	0.608870
s(Longitude, Latitude):Year2013	7.948158	8.634	6.783	< 2e-16 ***
s(Longitude, Latitude):Year2014	8.455177	8.890	7.350	< 2e-16 ***
s(Longitude, Latitude):Year2015	7.751819	8.490	5.094	8.78e-07 ***
s(Longitude, Latitude):Year2016	7.526591	8.316	3.669	0.000178 ***
s(Longitude, Latitude):Year2017	6.542483	7.623	1.676	0.057637 .
s(Longitude, Latitude):Year2018	2.000024	2.000	0.193	0.824282
s(FieldID)	0.001364	162.000	0.000	0.998897

Signif. codes: 0 ‘***’ 0.001 ‘**’ 0.01 ‘*’ 0.05 ‘.’ 0.1 ‘ ’ 1

R-sq.(adj) =   0.46   Deviance explained = 50.8%
-REML = 1023.4 Scale est. = 0.11906   n = 2176

7. Effects on *Coccus pseudomagnoliarum* densities

> summary(*CoccusDensity*)

Family: gaussian
Link function: identity

Formula:
gam(*CoccusDensity* ~ s(*FieldSize*) + *CitrusSpecies* + *PlantingAge* + *CitrusMatrix* + *NaturalMatrix* + s(*Year*, bs = "re") + *RanchID* + s(*FieldID*, bs = "re") + s(*Longitude*, *Latitude*, bs = "tp", by = *Year*, k = 10), method = "REML", data = *CitrusFieldSize*)

Parametric coefficients:

	Estimate	Std. Error	t value	Pr(>|t|)
(Intercept)	0.5379296	0.2448683	2.197	0.028315 *
CitrusMatrix	-0.0358760	0.0509960	-0.704	0.481943

NaturalMatrix	0.1074326	0.0554496	1.937	0.053032 .
CitrusSpecies-clementina	0.0306245	0.0386307	0.793	0.428154
CitrusSpecies-clementina x sinensis	0.0067724	0.0480572	0.141	0.887965
CitrusSpecies-reticulata	0.0197979	0.0364224	0.544	0.586890
CitrusSpecies-sinensis	0.0207346	0.0375717	0.552	0.581191
CitrusSpecies-tangelo	0.0629483	0.0469784	-1.340	0.180638
CitrusSpecies-unshiu	0.0269619	0.0438101	0.615	0.538445
PlantingAge	0.0011423	0.0003702	3.086	0.002100 **
RanchID8	0.0207443	0.1299272	0.160	0.873188
RanchID11	0.2740519	0.1263667	2.169	0.030394 *
RanchID12	0.2485035	0.1465595	1.696	0.090348 .
RanchID13	-0.3070683	4.3585967	-0.070	0.943852
RanchID14	-1.0386509	0.4042205	-2.570	0.010361 *
RanchID15	-0.6960968	1.1753718	-0.592	0.553857
RanchID16	-0.7147721	0.8252053	-0.866	0.386650
RanchID17	-0.2303325	4.4423914	-0.052	0.958662
RanchID19	-0.2388985	0.1639211	-1.457	0.145392
RanchID20	-0.2404988	0.1717161	-1.401	0.161727
RanchID21	0.7100488	0.1584961	4.480	8.54e-06 ***
RanchID23	-0.9442360	0.2683950	-3.518	0.000459 ***
RanchID24	-1.0125300	0.3136064	-3.229	0.001294 **
RanchID25	-0.9011635	0.2717053	-3.317	0.000951 ***
RanchID26	-0.6781884	0.3146907	-2.155	0.031447 *
RanchID27	-0.8910820	0.2647697	-3.365	0.000800 ***
RanchID28	-0.9493736	0.2749396	-3.453	0.000583 ***
RanchID29	-0.5404362	0.2972461	-1.818	0.069410 .
RanchID30	-0.7464919	0.2160190	-3.456	0.000577 ***
RanchID31	-0.8575724	0.2710557	-3.164	0.001615 **
RanchID32	-0.6207782	0.2890638	-2.148	0.032044 *
RanchID33	-1.0322860	0.2881760	-3.582	0.000361 ***
RanchID34	-0.8158162	0.2514297	-3.245	0.001224 **
RanchID35	-0.8281634	0.2177850	-3.803	0.000154 ***
RanchID36	-0.3287035	0.2834219	-1.160	0.246483
RanchID37	-0.4784328	0.2991665	-1.599	0.110160
RanchID38	-0.9549523	0.2763377	-3.456	0.000577 ***
RanchID39	-0.7231516	0.3438560	-2.103	0.035766 *
RanchID40	-0.6606829	0.3064719	-2.156	0.031394 *
RanchID41	-0.4929012	0.1693069	-2.911	0.003698 **
RanchID42	-0.8183988	0.2469687	-3.314	0.000961 ***
RanchID43	-0.7387464	0.2097308	-3.522	0.000452 ***
RanchID44	-0.6669083	0.4431978	-1.505	0.132773
RanchID45	-0.8604064	0.2222326	-3.872	0.000117 ***
RanchID46	-0.5201075	0.1785034	-2.914	0.003669 **
RanchID47	-0.8206285	0.2449843	-3.350	0.000846 ***

RanchID48	-0.3695248	0.1591029	-2.323	0.020449 *
RanchID49	-0.8581855	0.2487714	-3.450	0.000590 ***
RanchID50	-0.7995638	0.2287168	-3.496	0.000498 ***
RanchID51	-0.6987035	0.2104381	-3.320	0.000939 ***
RanchID52	-0.9345748	0.2646482	-3.531	0.000437 ***
RanchID53	-0.7225731	0.2115694	-3.415	0.000669 ***
RanchID55	-0.8955755	0.2619137	-3.419	0.000659 ***
RanchID56	-0.8946231	0.2879690	-3.107	0.001958 **
RanchID57	-0.8478225	0.2596676	-3.265	0.001140 **
RanchID58	-0.9872617	0.2571964	-3.839	0.000133 ***
RanchID59	-0.8966246	0.2689342	-3.334	0.000895 ***
RanchID60	-0.7928539	0.2321552	-3.415	0.000669 ***
RanchID62	-0.8733480	0.2546021	-3.430	0.000633 ***
RanchID63	-0.4248387	0.1642980	-2.586	0.009889 **
RanchID64	-0.9480317	0.2714121	-3.493	0.000503 ***
RanchID65	-0.8187007	0.2185919	-3.745	0.000193 ***
RanchID66	-0.8731122	0.2374859	-3.676	0.000252 ***
RanchID67	-0.9986282	0.2581422	-3.869	0.000118 ***
RanchID68	-0.4339251	0.1555353	-2.790	0.005396 **
RanchID69	-0.9853012	0.3079094	-3.200	0.001428 **
RanchID70	-0.4893572	0.1789407	-2.735	0.006379 **
RanchID71	-0.6705416	0.3088700	-2.171	0.030223 *
RanchID72	-0.6357879	0.3402183	-1.869	0.062014 .
RanchID73	-0.8575470	0.2581866	-3.321	0.000936 ***
RanchID74	-0.8335797	0.2595252	-3.212	0.001370 **
RanchID75	-0.7847670	0.2167316	-3.621	0.000312 ***
RanchID76	-0.3661056	0.2923250	-1.252	0.210787
RanchID77	-0.3497796	0.2941076	-1.189	0.234672
RanchID78	-0.3666990	0.2928520	-1.252	0.210869
RanchID79	-0.3022390	0.2785213	-1.085	0.278174
RanchID80	-0.3217940	0.2759076	-1.166	0.243830
RanchID81	-0.3483625	0.2846242	-1.224	0.221330
RanchID82	-0.7353679	0.4357546	-1.688	0.091876 .
RanchID83	-0.9930929	0.3060713	-3.245	0.001224 **

Signif. codes: 0 ‘***’ 0.001 ‘**’ 0.01 ‘*’ 0.05 ‘.’ 0.1 ‘ ’ 1

Approximate significance of smooth terms:

	edf	Ref.df	F	p-value
s(FieldSize)	1.000	1.001	1.974	0.160369
s(Year)	4.358	11.000	2.099	1.12e-05 ***
s(Longitude, Latitude):Year2003	1.000	1.000	0.005	0.946151
s(Longitude, Latitude):Year2006	2.000	2.000	2.140	0.118463
s(Longitude, Latitude):Year2007	6.129	7.058	4.060	0.000227 ***

s(Longitude, Latitude):Year2008	2.000	2.000	3.601	0.027732 *
s(Longitude, Latitude):Year2009	2.059	2.115	4.184	0.013818 *
s(Longitude, Latitude):Year2010	6.135	7.092	6.028	9.98e-07 ***
s(Longitude, Latitude):Year2011	7.007	7.654	14.922	< 2e-16 ***
s(Longitude, Latitude):Year2012	4.234	4.734	4.859	0.000267 ***
s(Longitude, Latitude):Year2013	4.426	5.558	2.713	0.016384 *
s(Longitude, Latitude):Year2014	2.001	2.002	5.078	0.006419 **
s(Longitude, Latitude):Year2015	2.000	2.000	4.949	0.007302 **
s(Longitude, Latitude):Year2016	2.741	3.188	3.948	0.007877 **
s(Longitude, Latitude):Year2017	2.000	2.000	5.249	0.005430 **
s(FieldID)	17.908	130.000	0.167	0.081163 .

Signif. codes: 0 ‘***’ 0.001 ‘**’ 0.01 ‘*’ 0.05 ‘.’ 0.1 ‘ ’ 1

Rank: 429/430
R-sq.(adj) = 0.576   Deviance explained =   64%
-REML = -814.51 Scale est. = 0.0059916 n = 961

8. Effects on insecticides targeting *Coccus pseudomagnoliarum*

> summary(*CoccusInsecticides*)

Family: gaussian
Link function: identity

Formula:
gam(*CoccusInsecticides* ~ s(*FieldSize*) + *CitrusSpecies* + *PlantingAge* + *CitrusMatrix* + *NaturalMatrix* + s(*Year*, bs = "re") + *RanchID* + s(*FieldID*, bs = "re") + s(*Longitude*, *Latitude*, bs = "tp", by = *Year*, k = 10), method = "REML", data = *CitrusFieldSize*)

Parametric coefficients:

	Estimate	Std. Error	t value	Pr(>|t|)
(Intercept)	0.2979325	0.4875071	0.611	0.541180
CitrusMatrix	0.1480159	0.1039712	1.424	0.154712
NaturalMatrix	0.1155429	0.1294231	0.893	0.372097
CitrusSpecies-clementina	0.0120273	0.0739878	0.163	0.870883
CitrusSpecies-clementina x sinensis	-0.2258942	0.1010924	-2.235	0.025559 *
CitrusSpecies-limettioides	-0.1716546	0.1950896	-0.880	0.379033
CitrusSpecies-limon	-0.2252686	0.1391836	-1.618	0.105713
CitrusSpecies-maxima	-0.2652557	0.1252991	-2.117	0.034385 *
CitrusSpecies-meyeri	-0.0470388	0.1962126	-0.240	0.810561
CitrusSpecies-reticulata	-0.0507705	0.0704833	-0.720	0.471413

CitrusSpecies-sinensis	-0.1248721	0.0681400	-1.833	0.067014 .
CitrusSpecies-tangelo	-0.1277662	0.1046679	-1.221	0.222351
CitrusSpecies-unshiu	-0.1311971	0.0863880	-1.519	0.128998
PlantingAge	0.0010235	0.0007766	1.318	0.187682
RanchID1	0.0378130	0.0971321	0.389	0.697099
RanchID2	0.0249922	0.3289994	0.076	0.939455
RanchID5	0.2121428	0.2388223	0.888	0.374494
RanchID6	-0.2366901	0.2185060	-1.083	0.278842
RanchID8	0.1823831	0.2579995	0.707	0.479703
RanchID9	0.8002978	0.2358536	3.393	0.000704 ***
RanchID11	0.0008831	0.1982781	0.004	0.996447
RanchID12	0.8169252	0.2874912	2.842	0.004535 **
RanchID13	-0.2534752	0.1517881	-1.670	0.095091 .
RanchID14	0.1654743	0.8972819	0.184	0.853705
RanchID15	-0.0186213	0.6836574	-0.027	0.978273
RanchID16	-0.0484680	0.6830205	-0.071	0.943436
RanchID17	-0.2328884	0.2080443	-1.119	0.263097
RanchID19	-0.0584738	0.2609195	-0.224	0.822697
RanchID20	-0.1351738	0.2175214	-0.621	0.534389
RanchID21	0.3476875	0.1982813	1.754	0.079669 .
RanchID22	0.0757096	0.3458649	0.219	0.826751
RanchID23	-0.1224232	0.5847624	-0.209	0.834192
RanchID24	-0.2091386	0.7036826	-0.297	0.766340
RanchID25	-0.2610142	0.5937046	-0.440	0.660248
RanchID26	-0.2253989	0.6937737	-0.325	0.745300
RanchID27	-0.1957953	0.5625243	-0.348	0.727828
RanchID29	-0.1344560	0.6392481	-0.210	0.833428
RanchID30	-0.3072293	0.4357302	-0.705	0.480836
RanchID31	-0.3088706	0.5675536	-0.544	0.586355
RanchID32	-0.0624508	0.6224723	-0.100	0.920095
RanchID33	-0.1643794	0.6374684	-0.258	0.796539
RanchID34	-0.3962655	0.5150629	-0.769	0.441775
RanchID36	-0.1654946	0.5958679	-0.278	0.781243
RanchID37	-0.1795775	0.6483322	-0.277	0.781821
RanchID38	-0.1887616	0.6055361	-0.312	0.755281
RanchID39	-0.0837643	0.7705138	-0.109	0.913442
RanchID40	-0.2299346	0.6683024	-0.344	0.730839
RanchID41	-0.3277555	0.2662876	-1.231	0.218531
RanchID42	-0.3243892	0.5249635	-0.618	0.536694
RanchID43	-0.1955383	0.4157738	-0.470	0.638192
RanchID44	-0.1395187	1.0175149	-0.137	0.890952
RanchID45	-0.3246618	0.4510856	-0.720	0.471773
RanchID46	-0.2108074	0.3018881	-0.698	0.485073
RanchID47	-0.1810483	0.5206387	-0.348	0.728070

RanchID48	-0.3126941	0.2130864	-1.467	0.142411
RanchID49	-0.3649152	0.5035017	-0.725	0.468688
RanchID50	-0.2294096	0.4729346	-0.485	0.627675
RanchID51	-0.3375796	0.4180000	-0.808	0.419413
RanchID52	-0.1804588	0.5732483	-0.315	0.752946
RanchID53	-0.2388180	0.4212514	-0.567	0.570829
RanchID54	-0.3177870	0.5949324	-0.534	0.593293
RanchID55	-0.2383876	0.5399281	-0.442	0.658887
RanchID56	-0.3138866	0.6017553	-0.522	0.601994
RanchID57	-0.1999311	0.5667619	-0.353	0.724305
RanchID58	-0.3210789	0.5525096	-0.581	0.561220
RanchID59	-0.0771390	0.5585908	-0.138	0.890179
RanchID60	-0.2529274	0.4636909	-0.545	0.585494
RanchID61	-0.1306336	0.8255869	-0.158	0.874291
RanchID62	-0.1255299	0.5739468	-0.219	0.826896
RanchID63	-0.1296625	0.2316789	-0.560	0.575771
RanchID64	-0.2365971	0.5946845	-0.398	0.690781
RanchID65	-0.2433002	0.4428848	-0.549	0.582825
RanchID66	-0.1679205	0.4944773	-0.340	0.734200
RanchID67	-0.3418576	0.5563630	-0.614	0.538988
RanchID68	-0.1266395	0.1975093	-0.641	0.521478
RanchID69	-0.2025829	0.6894623	-0.294	0.768920
RanchID70	-0.2210443	0.3032121	-0.729	0.466082
RanchID71	-0.0680445	0.6742534	-0.101	0.919626
RanchID72	-0.0907452	0.7589556	-0.120	0.904839
RanchID73	-0.2283796	0.5570515	-0.410	0.681865
RanchID74	-0.3776993	0.5377663	-0.702	0.482544
RanchID75	-0.2679762	0.4390428	-0.610	0.541690
RanchID76	-0.1018105	0.6192976	-0.164	0.869435
RanchID77	-0.1749880	0.6232571	-0.281	0.778921
RanchID78	-0.0848613	0.6230436	-0.136	0.891673
RanchID79	-0.1840984	0.5794899	-0.318	0.750753
RanchID80	-0.1410427	0.5741628	-0.246	0.805979
RanchID81	-0.1523933	0.5985648	-0.255	0.799060
RanchID82	0.0820942	1.0000231	0.082	0.934582
RanchID83	-0.1740041	0.6832889	-0.255	0.799015

Signif. codes: 0 '***' 0.001 '**' 0.01 '*' 0.05 '.' 0.1 ' ' 1

Approximate significance of smooth terms:

	edf	Ref.df	F	p-value
s(FieldSize)	1.001e+00	1.001	0.056	0.813781
s(Year)	1.300e+01	15.000	44.937	< 2e-16 ***
s(Longitude, Latitude):Year2003	4.968e+00	5.550	4.563	0.000238 ***

s(Longitude, Latitude):Year2004	4.963e+00	5.710	2.081	0.039832 *
s(Longitude, Latitude):Year2005	5.937e+00	6.969	1.544	0.153543
s(Longitude, Latitude):Year2006	6.828e+00	7.676	3.294	0.000998 ***
s(Longitude, Latitude):Year2007	7.275e+00	8.039	5.877	< 2e-16 ***
s(Longitude, Latitude):Year2008	2.000e+00	2.001	0.029	0.972023
s(Longitude, Latitude):Year2009	8.317e+00	8.875	9.155	< 2e-16 ***
s(Longitude, Latitude):Year2010	4.635e+00	5.947	1.211	0.293455
s(Longitude, Latitude):Year2011	2.851e+00	3.506	0.122	0.962595
s(Longitude, Latitude):Year2012	4.415e+00	5.658	0.801	0.593439
s(Longitude, Latitude):Year2013	8.190e+00	8.764	20.586	< 2e-16 ***
s(Longitude, Latitude):Year2014	3.837e+00	4.944	0.770	0.595902
s(Longitude, Latitude):Year2015	8.263e+00	8.786	3.443	0.000198 ***
s(Longitude, Latitude):Year2016	3.041e+00	3.807	0.147	0.959109
s(Longitude, Latitude):Year2017	2.000e+00	2.000	0.035	0.965591
s(Longitude, Latitude):Year2018	2.000e+00	2.000	4.557	0.010604 *
s(FieldID)	9.717e-04	162.000	0.000	0.998199

Signif. codes: 0 '***' 0.001 '**' 0.01 '*' 0.05 '.' 0.1 ' ' 1

R-sq.(adj) = 0.557   Deviance explained = 59.5%
-REML = 680.8 Scale est. = 0.086386 n = 2176

9. Effects on *Aonidiella aurantii* densities

> summary(*AonidiellaDensity*)

Family: gaussian
Link function: identity

Formula:
gam(*AonidiellaDensity* ~ s(*FieldSize*) + *CitrusSpecies* + *PlantingAge* + *CitrusMatrix* + *NaturalMatrix* + s(*Year*, bs = "re") + *RanchID* + s(*FieldID*, bs = "re") + s(*Longitude*, *Latitude*, bs = "tp", by = *Year*, k = 10), method = "REML", data = *CitrusFieldSize*)

Parametric coefficients:

	Estimate	Std. Error	t value	Pr(>|t|)
(Intercept)	-15.8312	23.2114	-0.682	0.4955
CitrusMatrix	-4.2148	2.0267	-2.080	0.0379 *
NaturalMatrix	-0.7710	2.8143	-0.274	0.7842
CitrusSpecies-clementina	2.8539	3.8201	0.747	0.4553
CitrusSpecies-clementina x sinensis	0.8612	4.3146	0.200	0.8419

CitrusSpecies-reticulata	2.0037	3.6659	0.547	0.5849
CitrusSpecies-sinensis	5.7219	3.7492	1.526	0.1275
CitrusSpecies-tangelo	3.3130	4.2218	0.785	0.4329
CitrusSpecies-unshiu	-0.7862	4.2326	-0.186	0.8527
PlantingAge	-0.2047	0.0272	-7.523	1.8e-13 ***
RanchID23	6.1595	20.4818	0.301	0.7637
RanchID24	13.2196	24.5947	0.537	0.5911
RanchID25	6.9352	21.4259	0.324	0.7463
RanchID26	38.1312	27.2677	1.398	0.1625
RanchID27	-6.2892	11.9162	-0.528	0.5978
RanchID28	2.4561	20.1981	0.122	0.9033
RanchID29	27.1498	26.7476	1.015	0.3105
RanchID30	7.4925	16.8173	0.446	0.6561
RanchID31	-6.7929	11.1469	-0.609	0.5425
RanchID32	18.7449	25.6419	0.731	0.4650
RanchID33	4.2325	21.3651	0.198	0.8430
RanchID34	0.5259	10.6343	0.049	0.9606
RanchID35	5.8803	15.8510	0.371	0.7108
RanchID36	27.5614	26.2502	1.050	0.2941
RanchID37	27.5388	26.4993	1.039	0.2991
RanchID38	5.5168	21.1647	0.261	0.7944
RanchID39	20.8456	29.3081	0.711	0.4772
RanchID40	38.8665	26.8767	1.446	0.1486
RanchID41	9.8443	14.4961	0.679	0.4973
RanchID42	8.4848	19.2523	0.441	0.6596
RanchID43	5.5590	16.3951	0.339	0.7347
RanchID44	30.9586	37.0303	0.836	0.4034
RanchID45	2.8808	14.4909	0.199	0.8425
RanchID46	9.4175	15.4629	0.609	0.5427
RanchID47	7.4595	19.4049	0.384	0.7008
RanchID48	13.3361	14.4410	0.923	0.3561
RanchID49	-4.2660	10.4167	-0.410	0.6823
RanchID50	8.9903	17.6619	0.509	0.6109
RanchID51	6.7210	17.3716	0.387	0.6990
RanchID52	6.2260	20.3138	0.306	0.7593
RanchID53	9.0732	17.0938	0.531	0.5958
RanchID55	-8.1589	11.2349	-0.726	0.4680
RanchID56	-10.7960	11.7690	-0.917	0.3593
RanchID57	12.0443	21.2002	0.568	0.5701
RanchID58	2.6334	18.2163	0.145	0.8851
RanchID59	-8.6455	11.6859	-0.740	0.4597
RanchID60	-2.7028	10.7579	-0.251	0.8017
RanchID62	7.7911	20.0702	0.388	0.6980
RanchID63	9.7999	14.1570	0.692	0.4890

RanchID64	5.8719	20.6800	0.284	0.7765
RanchID65	4.8463	15.6177	0.310	0.7564
RanchID66	7.8808	18.0218	0.437	0.6620
RanchID67	2.0883	18.4321	0.113	0.9098
RanchID68	10.0803	11.9201	0.846	0.3981
RanchID69	9.6393	24.2194	0.398	0.6908
RanchID70	10.1430	15.7794	0.643	0.5206
RanchID71	22.7682	27.0652	0.841	0.4005
RanchID72	21.4939	29.5169	0.728	0.4668
RanchID73	7.4843	20.4122	0.367	0.7140
RanchID74	-7.4184	9.9340	-0.747	0.4555
RanchID75	13.9931	16.5128	0.847	0.3971
RanchID76	24.0248	26.8956	0.893	0.3721
RanchID77	26.0080	27.0668	0.961	0.3370
RanchID78	24.9803	26.8313	0.931	0.3522
RanchID79	25.7373	25.9049	0.994	0.3208
RanchID80	24.3165	25.6568	0.948	0.3436
RanchID81	22.6193	26.2568	0.861	0.3893
RanchID82	29.5983	36.4483	0.812	0.4171
RanchID83	8.8971	23.9508	0.371	0.7104

Signif. codes: 0 ‘***’ 0.001 ‘**’ 0.01 ‘*’ 0.05 ‘.’ 0.1 ‘ ’ 1

Approximate significance of smooth terms:

	edf	Ref.df	F	p-value
s(FieldSize)	1.000	1.000	1.529	0.216675
s(Year)	7.166	10.000	8.264	< 2e-16 ***
s(Longitude, Latitude):Year2007	2.000	2.000	0.580	0.560254
s(Longitude, Latitude):Year2008	2.000	2.000	0.742	0.476583
s(Longitude, Latitude):Year2009	2.000	2.000	0.623	0.536743
s(Longitude, Latitude):Year2010	2.000	2.000	0.601	0.548441
s(Longitude, Latitude):Year2011	2.000	2.000	0.628	0.533919
s(Longitude, Latitude):Year2012	2.000	2.000	0.567	0.567712
s(Longitude, Latitude):Year2013	2.520	2.922	0.491	0.657175
s(Longitude, Latitude):Year2014	2.000	2.000	0.609	0.544169
s(Longitude, Latitude):Year2015	5.530	6.744	1.436	0.159345
s(Longitude, Latitude):Year2016	7.341	8.129	3.658	0.000425 ***
s(Longitude, Latitude):Year2017	2.118	2.220	33.957	< 2e-16 ***
s(FieldID)	39.979	115.000	0.605	5.28e-05 ***

Signif. codes: 0 ‘***’ 0.001 ‘**’ 0.01 ‘*’ 0.05 ‘.’ 0.1 ‘ ’ 1

R-sq.(adj) = 0.419   Deviance explained = 52.8%
-REML = 2134.1 Scale est. = 16.587   n = 793

10. Effects on insecticides targeting *Aonidiella aurantii*

> summary(*AonidiellaInsecticides*)

Family: gaussian
Link function: identity

Formula:
gam(*AonidiellaInsecticides* ~ s(*FieldSize*) + *CitrusSpecies* + *PlantingAge* + *CitrusMatrix* + *NaturalMatrix* + s(*Year*, bs = "re") + *RanchID* + s(*FieldID*, bs = "re") + s(*Longitude*, *Latitude*, bs = "tp", by = *Year*, k = 10), method = "REML", data = *CitrusFieldSize*)

Parametric coefficients:

	Estimate	Std. Error	t value	Pr(>|t|)
(Intercept)	0.5757946	0.9606244	0.599	0.548977
CitrusMatrix	0.3739979	0.2242339	1.668	0.095495 .
NaturalMatrix	-0.2941508	0.2602744	-1.130	0.258546
CitrusSpecies-clementina	0.1064236	0.1427797	0.745	0.456137
CitrusSpecies-clementina x sinensis	0.0270339	0.1951392	0.139	0.889830
CitrusSpecies-limettioides	0.2377745	0.3711336	0.641	0.521810
CitrusSpecies-limon	0.3446015	0.2674895	1.288	0.197798
CitrusSpecies-maxima	0.5925171	0.2432633	2.436	0.014950 *
CitrusSpecies-meyeri	0.4153929	0.3729385	1.114	0.265483
CitrusSpecies-reticulata	0.1348901	0.1360151	0.992	0.321451
CitrusSpecies-sinensis	0.2938606	0.1314161	2.236	0.025455 *
CitrusSpecies-tangelo	0.2605044	0.2028538	1.284	0.199222
CitrusSpecies-unshiu	-0.1035169	0.1665193	-0.622	0.534242
PlantingAge	0.0037230	0.0015046	2.474	0.013428 *
RanchID1	0.5529684	0.1890242	2.925	0.003479 **
RanchID2	1.2948070	0.6380143	2.029	0.042547 *
RanchID5	0.2051165	0.4608982	0.445	0.656342
RanchID6	0.2115873	0.4212496	0.502	0.615523
RanchID8	0.4813408	0.5027482	0.957	0.338472
RanchID9	-0.3038508	0.4638117	-0.655	0.512468
RanchID11	0.0725830	0.3849883	0.189	0.850478
RanchID12	0.6939370	0.5776441	1.201	0.229769
RanchID13	0.9585008	0.2962642	3.235	0.001235 **
RanchID14	-0.4680754	1.7661378	-0.265	0.791016
RanchID15	-0.8009510	1.3444515	-0.596	0.551413
RanchID16	-0.6237817	1.3428097	-0.465	0.642316
RanchID17	1.5054780	0.4034115	3.732	0.000195 ***
RanchID19	-0.7242196	0.5092249	-1.422	0.155125

RanchID20	-0.8021447	0.4227836	-1.897	0.057934 .
RanchID21	-0.5635015	0.3881185	-1.452	0.146693
RanchID22	-0.3390502	0.6722866	-0.504	0.614090
RanchID23	0.0007849	1.1492610	0.001	0.999455
RanchID24	0.2978159	1.3838500	0.215	0.829627
RanchID25	-0.0669862	1.1673089	-0.057	0.954244
RanchID26	-1.1586469	1.3641285	-0.849	0.395779
RanchID27	2.5256351	1.1071225	2.281	0.022638 *
RanchID29	-1.2307832	1.2571363	-0.979	0.327681
RanchID30	0.5535045	0.8565296	0.646	0.518213
RanchID31	2.8259000	1.1176119	2.529	0.011531 *
RanchID32	-0.4374405	1.2241796	-0.357	0.720880
RanchID33	0.4521297	1.2524863	0.361	0.718148
RanchID34	1.7172347	1.0139560	1.694	0.090498 .
RanchID36	-1.0936730	1.1717328	-0.933	0.350737
RanchID37	-0.8851468	1.2751188	-0.694	0.487658
RanchID38	0.5718916	1.1897299	0.481	0.630789
RanchID39	-0.7547928	1.5161074	-0.498	0.618645
RanchID40	-1.3221512	1.3149963	-1.005	0.314807
RanchID41	0.2406602	0.5221492	0.461	0.644918
RanchID42	0.3587116	1.0319403	0.348	0.728171
RanchID43	0.7002043	0.8173573	0.857	0.391731
RanchID44	-1.9109133	2.0041450	-0.953	0.340462
RanchID45	1.8506956	0.8863946	2.088	0.036934 *
RanchID46	-0.0081694	0.5925818	-0.014	0.989002
RanchID47	0.1530374	1.0226267	0.150	0.881055
RanchID48	0.1679885	0.4192084	0.401	0.688664
RanchID49	2.0675838	0.9910091	2.086	0.037075 *
RanchID50	1.0855947	0.9297223	1.168	0.243086
RanchID51	0.8122916	0.8220066	0.988	0.323184
RanchID52	0.0064352	1.1266080	0.006	0.995443
RanchID53	0.3745083	0.8285009	0.452	0.651296
RanchID54	-0.3275084	1.1632918	-0.282	0.778329
RanchID55	1.9794373	1.0628819	1.862	0.062704 .
RanchID56	2.3261387	1.1849995	1.963	0.049787 *
RanchID57	0.3651288	1.1135874	0.328	0.743033
RanchID58	0.9521094	1.0854403	0.877	0.380503
RanchID59	2.4108337	1.0999059	2.192	0.028505 *
RanchID60	1.5273389	0.9092582	1.680	0.093160 .
RanchID61	-1.5679191	1.6207725	-0.967	0.333467
RanchID62	-0.6015558	1.1245048	-0.535	0.592743
RanchID63	-0.0163779	0.4546116	-0.036	0.971265
RanchID64	0.0701127	1.1685579	0.060	0.952162
RanchID65	0.9183159	0.8701866	1.055	0.291412

RanchID66	1.3921826	0.9720863	1.432	0.152255
RanchID67	0.6047284	1.0928284	0.553	0.580079
RanchID68	0.2695182	0.3870757	0.696	0.486326
RanchID69	0.2861195	1.3554602	0.211	0.832841
RanchID70	-0.2734573	0.5961302	-0.459	0.646485
RanchID71	-0.5984176	1.3266386	-0.451	0.651982
RanchID72	-0.9603965	1.4935630	-0.643	0.520283
RanchID73	-0.0176772	1.0949816	-0.016	0.987121
RanchID74	2.2111353	1.0593742	2.087	0.036996 *
RanchID75	0.3267445	0.8638600	0.378	0.705294
RanchID76	-1.1138664	1.2179887	-0.915	0.360558
RanchID77	-0.9150540	1.2261971	-0.746	0.455602
RanchID78	-0.8597526	1.2254481	-0.702	0.483022
RanchID79	-0.7867903	1.1397841	-0.690	0.490087
RanchID80	-0.8759466	1.1288561	-0.776	0.437865
RanchID81	-0.9703662	1.1772051	-0.824	0.409870
RanchID82	-1.7232358	1.9698399	-0.875	0.381783
RanchID83	0.4850662	1.3432637	0.361	0.718055

Signif. codes: 0 ‘***’ 0.001 ‘**’ 0.01 ‘*’ 0.05 ‘.’ 0.1 ‘ ’ 1

Approximate significance of smooth terms:

	edf	Ref.df	F	p-value
s(FieldSize)	1.000	1.001	2.506	0.113553
s(Year)	13.678	15.000	37.233	< 2e-16 ***
s(Longitude, Latitude):Year2003	2.000	2.000	1.165	0.312162
s(Longitude, Latitude):Year2004	2.000	2.001	0.989	0.372295
s(Longitude, Latitude):Year2005	3.959	4.961	1.196	0.353193
s(Longitude, Latitude):Year2006	3.694	4.594	1.211	0.268747
s(Longitude, Latitude):Year2007	5.589	6.700	2.013	0.059191 .
s(Longitude, Latitude):Year2008	3.758	4.849	1.281	0.279799
s(Longitude, Latitude):Year2009	2.005	2.009	2.011	0.133847
s(Longitude, Latitude):Year2010	6.680	7.938	3.324	0.000766 ***
s(Longitude, Latitude):Year2011	7.360	8.384	7.276	< 2e-16 ***
s(Longitude, Latitude):Year2012	7.705	8.545	1.867	0.029918 *
s(Longitude, Latitude):Year2013	2.001	2.001	1.674	0.187648
s(Longitude, Latitude):Year2014	2.001	2.001	1.541	0.214426
s(Longitude, Latitude):Year2015	7.107	8.060	2.643	0.008104 **
s(Longitude, Latitude):Year2016	7.085	8.027	4.500	1.73e-05 ***
s(Longitude, Latitude):Year2017	6.296	7.444	3.835	0.000316 ***
s(Longitude, Latitude):Year2018	2.000	2.000	0.529	0.589442
s(FieldID)	9.641	162.000	0.064	0.261365

Signif. codes: 0 ‘***’ 0.001 ‘**’ 0.01 ‘*’ 0.05 ‘.’ 0.1 ‘ ’ 1

R-sq.(adj) = 0.478   Deviance explained = 52.2%
-REML = 1975.1 Scale est. = 0.308   n = 2176

11. Effects on *Icerya purchasi* densities

> summary(*IceryaDensity*)

Family: gaussian
Link function: identity

Formula:
gam(*IceryaDensity* ~ s(*FieldSize*) + *CitrusSpecies* + *PlantingAge* + *CitrusMatrix* + *NaturalMatrix* + s(*Year*, bs = "re") + *RanchID* + s(*FieldID*, bs = "re") + s(*Longitude*, *Latitude*, bs = "tp", by = *Year*, k = 10), method = "REML", data = *CitrusFieldSize*)

Parametric coefficients:

	Estimate	Std. Error	t value	Pr(>|t|)
(Intercept)	-9.421e-02	1.576e-01	-0.598	0.550131
CitrusMatrix	-2.224e-02	4.357e-02	-0.510	0.609878
NaturalMatrix	-1.581e-02	5.462e-02	-0.289	0.772382
CitrusSpecies-clementina	9.409e-02	4.728e-02	1.990	0.046993 *
CitrusSpecies-clementina x sinensis	-2.086e-03	5.254e-02	-0.040	0.968334
CitrusSpecies-reticulata	7.726e-02	4.582e-02	1.686	0.092221 .
CitrusSpecies-sinensis	1.554e-02	4.640e-02	0.335	0.737714
CitrusSpecies-tangelo	4.176e-02	5.900e-02	0.708	0.479317
CitrusSpecies-unshiu	-3.815e-03	5.263e-02	-0.072	0.942235
PlantingAge	4.252e-05	3.745e-04	0.114	0.909650
RanchID24	9.644e-02	8.506e-02	1.134	0.257289
RanchID25	3.167e-02	5.659e-02	0.560	0.575856
RanchID26	1.102e-01	1.833e-01	0.602	0.547693
RanchID27	3.716e-02	2.457e-01	0.151	0.879846
RanchID28	2.061e-02	6.929e-02	0.297	0.766213
RanchID29	1.139e-01	2.243e-01	0.508	0.611766
RanchID30	4.838e-02	9.493e-02	0.510	0.610444
RanchID31	4.226e-02	2.798e-01	0.151	0.879995
RanchID32	1.020e-01	1.850e-01	0.551	0.581581
RanchID33	6.460e-02	6.886e-02	0.938	0.348510
RanchID34	9.956e-02	2.494e-01	0.399	0.689877
RanchID35	1.090e-01	1.140e-01	0.957	0.339067

RanchID36	1.332e-01	2.626e-01	0.507	0.612104
RanchID37	1.079e-01	1.983e-01	0.544	0.586635
RanchID38	4.850e-02	5.291e-02	0.917	0.359592
RanchID39	9.545e-02	1.985e-01	0.481	0.630724
RanchID40	2.712e-01	1.957e-01	1.386	0.166298
RanchID41	9.986e-02	1.660e-01	0.601	0.547803
RanchID42	1.943e-02	7.238e-02	0.269	0.788376
RanchID43	4.443e-02	9.569e-02	0.464	0.642540
RanchID44	1.254e-01	3.163e-01	0.397	0.691798
RanchID45	5.498e-02	1.219e-01	0.451	0.652067
RanchID46	1.222e-01	1.492e-01	0.819	0.413108
RanchID47	2.490e-01	6.251e-02	3.983	7.56e-05 ***
RanchID48	8.019e-02	2.044e-01	0.392	0.695004
RanchID49	3.231e-02	2.429e-01	0.133	0.894236
RanchID50	5.147e-02	8.024e-02	0.641	0.521447
RanchID51	6.668e-02	1.034e-01	0.645	0.519304
RanchID52	2.609e-01	6.730e-02	3.876	0.000117 ***
RanchID53	4.969e-02	9.324e-02	0.533	0.594256
RanchID55	4.748e-02	2.497e-01	0.190	0.849223
RanchID56	4.148e-02	2.986e-01	0.139	0.889565
RanchID57	6.600e-02	7.360e-02	0.897	0.370186
RanchID58	3.082e-02	8.057e-02	0.382	0.702227
RanchID59	9.133e-03	2.588e-01	0.035	0.971854
RanchID60	1.067e-01	2.272e-01	0.470	0.638847
RanchID62	3.397e-01	7.837e-02	4.334	1.69e-05 ***
RanchID63	1.095e-01	1.933e-01	0.567	0.571084
RanchID64	1.269e-01	6.531e-02	1.944	0.052359 .
RanchID65	2.210e-02	1.013e-01	0.218	0.827317
RanchID66	4.972e-02	6.823e-02	0.729	0.466487
RanchID67	1.768e-01	7.963e-02	2.221	0.026712 *
RanchID68	1.013e-01	1.926e-01	0.526	0.599048
RanchID69	7.317e-02	8.260e-02	0.886	0.376036
RanchID70	6.325e-02	1.705e-01	0.371	0.710765
RanchID71	1.057e-01	1.985e-01	0.533	0.594458
RanchID72	1.030e-01	2.195e-01	0.469	0.638943
RanchID73	2.277e-02	6.308e-02	0.361	0.718280
RanchID74	2.428e-02	2.771e-01	0.088	0.930200
RanchID75	1.284e-01	8.832e-02	1.454	0.146510
RanchID76	1.348e-01	2.647e-01	0.509	0.610773
RanchID77	1.184e-01	2.745e-01	0.432	0.666222
RanchID78	1.145e-01	2.561e-01	0.447	0.654778
RanchID79	1.129e-01	2.704e-01	0.418	0.676359
RanchID80	1.168e-01	2.595e-01	0.450	0.652756
RanchID81	1.127e-01	2.551e-01	0.442	0.658844

RanchID82	1.219e-01	3.045e-01	0.400	0.688982
RanchID83	5.065e-02	8.106e-02	0.625	0.532299

Signif. codes: 0 ‘***’ 0.001 ‘**’ 0.01 ‘*’ 0.05 ‘.’ 0.1 ‘ ’ 1

Approximate significance of smooth terms:

	edf	Ref.df	F	p-value
s(FieldSize)	1.000010	1.000	0.503	0.4784
s(Year)	3.318957	8.000	2.190	9.99e-05 ***
s(Longitude, Latitude):Year2009	2.000021	2.000	0.045	0.9556
s(Longitude, Latitude):Year2010	2.000021	2.000	0.033	0.9677
s(Longitude, Latitude):Year2011	2.000029	2.000	0.072	0.9304
s(Longitude, Latitude):Year2012	2.000016	2.000	0.045	0.9561
s(Longitude, Latitude):Year2013	6.404847	7.411	2.243	0.0676 .
s(Longitude, Latitude):Year2014	2.000014	2.000	0.003	0.9973
s(Longitude, Latitude):Year2015	2.681379	2.898	1.402	0.3398
s(Longitude, Latitude):Year2016	2.827492	3.396	0.119	0.9458
s(Longitude, Latitude):Year2017	4.854251	6.118	0.772	0.6292
s(FieldID)	0.001242	115.000	0.000	0.9994

Signif. codes: 0 ‘***’ 0.001 ‘**’ 0.01 ‘*’ 0.05 ‘.’ 0.1 ‘ ’ 1

R-sq.(adj) = 0.343   Deviance explained = 42.9%
-REML = -621.25 Scale est. = 0.0063626 n = 750

12. Effects on insecticides targeting *Icerya purchasi*

> summary(*IceryaInsecticides*)

Family: gaussian
Link function: identity

Formula:
gam(*IceryaInsecticides* ~ s(*FieldSize*) + *CitrusSpecies* + *PlantingAge* + *CitrusMatrix* + *NaturalMatrix* + s(*Year*, bs = "re") + *RanchID* + s(*FieldID*, bs = "re") + s(*Longitude*, *Latitude*, bs = "tp", by = *Year*, k = 10), method = "REML", data = *CitrusFieldSize*)

Parametric coefficients:

	Estimate	Std. Error	t value	Pr(>|t|)
(Intercept)	1.661e-01	2.576e-01	0.645	0.51915
CitrusMatrix	-8.199e-02	5.274e-02	-1.555	0.12021
NaturalMatrix	-5.670e-02	6.408e-02	-0.885	0.37632
CitrusSpecies-clementina	8.300e-02	3.927e-02	2.114	0.03468 *

CitrusSpecies-clementina x sinensis	5.142e-02	5.367e-02	0.958	0.33813
CitrusSpecies-limettioides	5.932e-02	1.027e-01	0.578	0.56354
CitrusSpecies-limon	6.825e-02	7.275e-02	0.938	0.34829
CitrusSpecies-maxima	4.840e-02	6.640e-02	0.729	0.46612
CitrusSpecies-meyeri	9.428e-02	1.035e-01	0.911	0.36227
CitrusSpecies-reticulata	9.801e-02	3.741e-02	2.620	0.00887 **
CitrusSpecies-sinensis	5.693e-02	3.621e-02	1.572	0.11605
CitrusSpecies-tangelo	7.849e-02	5.550e-02	1.414	0.15742
CitrusSpecies-unshiu	2.932e-02	4.566e-02	0.642	0.52080
PlantingAge	-4.109e-05	4.075e-04	-0.101	0.91968
RanchID1	2.711e-02	5.038e-02	0.538	0.59066
RanchID2	2.888e-02	1.726e-01	0.167	0.86713
RanchID5	4.751e-03	1.271e-01	0.037	0.97018
RanchID6	-5.148e-02	1.156e-01	-0.445	0.65610
RanchID8	-3.476e-02	1.377e-01	-0.252	0.80074
RanchID9	-1.012e-01	1.257e-01	-0.805	0.42068
RanchID11	-6.179e-02	1.049e-01	-0.589	0.55578
RanchID12	-5.615e-02	1.554e-01	-0.361	0.71788
RanchID13	-8.268e-03	8.019e-02	-0.103	0.91790
RanchID14	-2.714e-01	4.775e-01	-0.568	0.56980
RanchID15	-2.171e-01	3.633e-01	-0.598	0.55016
RanchID16	-2.364e-01	3.630e-01	-0.651	0.51499
RanchID17	3.993e-02	1.090e-01	0.366	0.71429
RanchID19	-9.070e-02	1.387e-01	-0.654	0.51326
RanchID20	-2.230e-02	1.154e-01	-0.193	0.84670
RanchID21	-1.658e-02	1.056e-01	-0.157	0.87533
RanchID22	-2.218e-01	1.816e-01	-1.222	0.22197
RanchID23	-3.054e-01	3.104e-01	-0.984	0.32529
RanchID24	-3.114e-01	3.735e-01	-0.834	0.40447
RanchID25	-2.992e-01	3.153e-01	-0.949	0.34289
RanchID26	-2.106e-01	3.687e-01	-0.571	0.56794
RanchID27	-1.991e-01	2.987e-01	-0.667	0.50516
RanchID29	-1.449e-01	3.403e-01	-0.426	0.67024
RanchID30	-2.284e-01	2.304e-01	-0.991	0.32174
RanchID31	-5.938e-02	3.013e-01	-0.197	0.84379
RanchID32	-1.340e-01	3.314e-01	-0.404	0.68605
RanchID33	-3.037e-01	3.384e-01	-0.897	0.36962
RanchID34	-8.064e-02	2.732e-01	-0.295	0.76789
RanchID36	-1.260e-01	3.172e-01	-0.397	0.69114
RanchID37	-1.901e-01	3.449e-01	-0.551	0.58154
RanchID38	-2.797e-01	3.207e-01	-0.872	0.38329
RanchID39	-2.254e-01	4.097e-01	-0.550	0.58222
RanchID40	-2.276e-01	3.557e-01	-0.640	0.52235
RanchID41	-9.059e-02	1.408e-01	-0.644	0.51992

RanchID42	-1.029e-01	2.792e-01	-0.369	0.71239
RanchID43	-2.650e-02	2.196e-01	-0.121	0.90399
RanchID44	-2.748e-01	5.423e-01	-0.507	0.61238
RanchID45	-2.296e-01	2.384e-01	-0.963	0.33573
RanchID46	-1.048e-01	1.593e-01	-0.658	0.51054
RanchID47	-1.453e-01	2.759e-01	-0.527	0.59851
RanchID48	-9.380e-02	1.128e-01	-0.831	0.40581
RanchID49	-1.176e-01	2.670e-01	-0.440	0.65964
RanchID50	-3.543e-01	2.509e-01	-1.412	0.15818
RanchID51	-2.051e-01	2.212e-01	-0.927	0.35385
RanchID52	-3.062e-01	3.038e-01	-1.008	0.31353
RanchID53	-1.571e-01	2.227e-01	-0.705	0.48067
RanchID54	-2.036e-01	3.150e-01	-0.646	0.51819
RanchID55	-9.651e-02	2.862e-01	-0.337	0.73596
RanchID56	-2.921e-01	3.197e-01	-0.914	0.36089
RanchID57	-5.798e-02	2.998e-01	-0.193	0.84668
RanchID58	5.701e-02	2.925e-01	0.195	0.84547
RanchID59	-1.845e-01	2.964e-01	-0.623	0.53360
RanchID60	-1.302e-01	2.458e-01	-0.530	0.59646
RanchID61	-3.100e-01	4.388e-01	-0.707	0.47995
RanchID62	-2.025e-01	3.042e-01	-0.666	0.50562
RanchID63	-8.173e-02	1.227e-01	-0.666	0.50554
RanchID64	-3.161e-01	3.157e-01	-1.001	0.31679
RanchID65	-2.395e-01	2.344e-01	-1.022	0.30705
RanchID66	-2.328e-01	2.623e-01	-0.888	0.37481
RanchID67	-1.806e-01	2.943e-01	-0.614	0.53953
RanchID68	-7.776e-02	1.039e-01	-0.749	0.45412
RanchID69	-2.651e-01	3.660e-01	-0.724	0.46902
RanchID70	-9.596e-02	1.598e-01	-0.600	0.54828
RanchID71	-2.012e-01	3.589e-01	-0.561	0.57516
RanchID72	-2.042e-01	4.043e-01	-0.505	0.61349
RanchID73	-1.362e-01	2.952e-01	-0.462	0.64449
RanchID74	-3.350e-01	2.861e-01	-1.171	0.24178
RanchID75	-2.560e-01	2.318e-01	-1.104	0.26967
RanchID76	-1.343e-01	3.298e-01	-0.407	0.68386
RanchID77	-1.244e-01	3.320e-01	-0.375	0.70797
RanchID78	-1.420e-01	3.321e-01	-0.427	0.66909
RanchID79	-1.302e-01	3.090e-01	-0.421	0.67361
RanchID80	-1.142e-01	3.057e-01	-0.374	0.70871
RanchID81	-1.246e-01	3.186e-01	-0.391	0.69582
RanchID82	-2.917e-01	5.329e-01	-0.547	0.58414
RanchID83	-2.196e-01	3.629e-01	-0.605	0.54513

Signif. codes: 0 ‘***’ 0.001 ‘**’ 0.01 ‘*’ 0.05 ‘.’ 0.1 ‘ ’ 1

Approximate significance of smooth terms:

	edf	Ref.df	F	p-value
s(FieldSize)	2.225e+00	2.813	1.370	0.36236
s(Year)	1.200e+01	15.000	6.477	< 2e-16 ***
s(Longitude, Latitude):Year2003	2.000e+00	2.000	0.215	0.80651
s(Longitude, Latitude):Year2004	2.000e+00	2.000	0.192	0.82542
s(Longitude, Latitude):Year2005	2.000e+00	2.000	0.172	0.84232
s(Longitude, Latitude):Year2006	2.000e+00	2.000	0.188	0.82868
s(Longitude, Latitude):Year2007	7.797e+00	8.392	6.857	2.53e-06 ***
s(Longitude, Latitude):Year2008	8.370e+00	8.903	10.901	< 2e-16 ***
s(Longitude, Latitude):Year2009	2.000e+00	2.000	0.241	0.78576
s(Longitude, Latitude):Year2010	7.148e+00	8.299	2.458	0.00933 **
s(Longitude, Latitude):Year2011	5.595e+00	6.966	1.440	0.17105
s(Longitude, Latitude):Year2012	7.415e+00	8.373	5.564	< 2e-16 ***
s(Longitude, Latitude):Year2013	2.000e+00	2.000	0.192	0.82541
s(Longitude, Latitude):Year2014	2.000e+00	2.000	0.169	0.84413
s(Longitude, Latitude):Year2015	2.000e+00	2.000	0.187	0.82924
s(Longitude, Latitude):Year2016	2.000e+00	2.000	0.163	0.84977
s(Longitude, Latitude):Year2017	2.000e+00	2.000	0.169	0.84420
s(Longitude, Latitude):Year2018	2.000e+00	2.000	0.214	0.80740
s(FieldID)	2.969e-04	162.000	0.000	0.99993

Signif. codes: 0 '***' 0.001 '**' 0.01 '*' 0.05 '.' 0.1 ' ' 1

R-sq.(adj) = 0.244   Deviance explained =   30%
-REML = -660.83 Scale est. = 0.024225 n = 2176

13. Effects on *Marmara gulosa* densities

> summary(*MarmaraDensity*)

Family: gaussian
Link function: identity

Formula:
gam(*MarmaraDensity* ~ s(*FieldSize*) + *CitrusSpecies* + *PlantingAge* + *CitrusMatrix* + *NaturalMatrix* + s(*Year*, bs = "re") + *RanchID* + s(*FieldID*, bs = "re") + s(*Longitude*, *Latitude*, bs = "tp", by = *Year*, k = 10), method = "REML", data = *CitrusFieldSize*)

Parametric coefficients:

	Estimate	Std. Error	t value	Pr(>|t|)
(Intercept)	29.918865	4.564536	6.555	1.13e-10 ***
CitrusMatrix	0.135222	0.187087	0.723	0.4701
NaturalMatrix	0.367665	0.276414	1.330	0.1839
CitrusSpecies-clementina	0.101054	0.445310	0.227	0.8205
CitrusSpecies-clementina x sinensis	0.249462	0.507366	0.492	0.6231
CitrusSpecies-reticulata	0.083494	0.427649	0.195	0.8453
CitrusSpecies-sinensis	0.257821	0.436121	0.591	0.5546
CitrusSpecies-tangelo	0.152369	0.484921	0.314	0.7535
CitrusSpecies-unshiu	0.010703	0.496576	0.022	0.9828
PlantingAge	-0.005382	0.003126	-1.722	0.0856 .
RanchID23	-28.214433	4.380550	-6.441	2.30e-10 ***
RanchID24	-28.739220	4.637916	-6.197	1.02e-09 ***
RanchID25	-28.565792	4.441221	-6.432	2.43e-10 ***
RanchID26	-30.608340	4.864269	-6.292	5.72e-10 ***
RanchID27	-25.870936	3.958074	-6.536	1.27e-10 ***
RanchID28	-27.712932	4.358778	-6.358	3.83e-10 ***
RanchID29	-31.224895	4.837170	-6.455	2.10e-10 ***
RanchID30	-27.944762	4.192599	-6.665	5.61e-11 ***
RanchID31	-24.506454	3.899181	-6.285	5.98e-10 ***
RanchID32	-30.947124	4.748648	-6.517	1.43e-10 ***
RanchID33	-27.949579	4.424745	-6.317	4.93e-10 ***
RanchID34	-24.812861	3.893850	-6.372	3.51e-10 ***
RanchID35	-27.151889	4.125507	-6.581	9.55e-11 ***
RanchID36	-31.853777	4.812875	-6.618	7.56e-11 ***
RanchID37	-31.050749	4.811737	-6.453	2.13e-10 ***
RanchID38	-28.271824	4.421426	-6.394	3.07e-10 ***
RanchID39	-31.125667	4.998572	-6.227	8.50e-10 ***
RanchID40	-31.264383	4.834286	-6.467	1.95e-10 ***
RanchID41	-28.523679	4.100645	-6.956	8.50e-12 ***
RanchID42	-28.280962	4.311263	-6.560	1.09e-10 ***
RanchID43	-28.069801	4.174574	-6.724	3.85e-11 ***
RanchID44	-33.011485	5.613680	-5.881	6.52e-09 ***
RanchID45	-27.372397	4.086263	-6.699	4.53e-11 ***
RanchID46	-28.688826	4.142731	-6.925	1.04e-11 ***
RanchID47	-28.428674	4.327994	-6.569	1.04e-10 ***
RanchID48	-28.836897	4.106648	-7.022	5.49e-12 ***
RanchID49	-25.548269	3.888620	-6.570	1.03e-10 ***
RanchID50	-27.989210	4.225663	-6.624	7.31e-11 ***
RanchID51	-28.492529	4.229101	-6.737	3.54e-11 ***
RanchID52	-28.166773	4.372589	-6.442	2.29e-10 ***
RanchID53	-28.139166	4.216815	-6.673	5.34e-11 ***
RanchID55	-23.473351	3.918884	-5.990	3.46e-09 ***
RanchID56	-24.277655	3.915290	-6.201	9.95e-10 ***

RanchID57	-28.472006	4.418352	-6.444	2.25e-10 ***
RanchID58	-27.418800	4.246576	-6.457	2.08e-10 ***
RanchID59	-24.667678	3.923028	-6.288	5.88e-10 ***
RanchID60	-25.370180	3.919559	-6.473	1.89e-10 ***
RanchID62	-26.341873	4.366722	-6.032	2.70e-09 ***
RanchID63	-28.293543	4.089012	-6.919	1.08e-11 ***
RanchID64	-28.181297	4.391208	-6.418	2.65e-10 ***
RanchID65	-27.738427	4.131866	-6.713	4.13e-11 ***
RanchID66	-28.040963	4.242481	-6.610	7.99e-11 ***
RanchID67	-27.563048	4.262031	-6.467	1.95e-10 ***
RanchID68	-28.040559	3.988839	-7.030	5.21e-12 ***
RanchID69	-28.882504	4.611758	-6.263	6.84e-10 ***
RanchID70	-28.843418	4.163761	-6.927	1.03e-11 ***
RanchID71	-31.198718	4.846309	-6.438	2.35e-10 ***
RanchID72	-31.627202	5.022733	-6.297	5.57e-10 ***
RanchID73	-28.420941	4.385220	-6.481	1.79e-10 ***
RanchID74	-24.897801	3.796514	-6.558	1.11e-10 ***
RanchID75	-27.926684	4.174984	-6.689	4.82e-11 ***
RanchID76	-32.030074	4.855323	-6.597	8.66e-11 ***
RanchID77	-32.103137	4.865281	-6.598	8.58e-11 ***
RanchID78	-31.923121	4.850643	-6.581	9.56e-11 ***
RanchID79	-31.801495	4.786577	-6.644	6.43e-11 ***
RanchID80	-31.782054	4.773986	-6.657	5.90e-11 ***
RanchID81	-31.897584	4.811717	-6.629	7.06e-11 ***
RanchID82	-32.946951	5.563395	-5.922	5.13e-09 ***
RanchID83	-28.900930	4.595659	-6.289	5.85e-10 ***

Signif. codes: 0 '***' 0.001 '**' 0.01 '*' 0.05 '.' 0.1 ' ' 1

Approximate significance of smooth terms:

	edf	Ref.df	F	p-value
s(FieldSize)	1.0001	1.000	0.088	0.76683
s(Year)	0.1206	9.000	0.014	0.36165
s(Longitude, Latitude):Year2007	2.0000	2.000	22.010	5.61e-10 ***
s(Longitude, Latitude):Year2008	2.0000	2.000	1.887	0.15228
s(Longitude, Latitude):Year2009	8.7999	8.975	22.838	< 2e-16 ***
s(Longitude, Latitude):Year2010	2.0001	2.000	2.069	0.12714
s(Longitude, Latitude):Year2011	2.0014	2.003	2.146	0.11777
s(Longitude, Latitude):Year2012	2.0001	2.000	2.270	0.10415
s(Longitude, Latitude):Year2013	5.3042	6.380	2.832	0.00746 **
s(Longitude, Latitude):Year2014	2.0001	2.000	2.237	0.10758
s(Longitude, Latitude):Year2015	2.0001	2.000	2.324	0.09864 .
s(Longitude, Latitude):Year2016	2.0002	2.000	2.383	0.09309 .
s(Longitude, Latitude):Year2017	2.0000	2.000	1.339	0.26285

s(FieldID)	16.5156	115.000	0.166	0.16155

Signif. codes: 0 ‘***’ 0.001 ‘**’ 0.01 ‘*’ 0.05 ‘.’ 0.1 ‘ ’ 1

R-sq.(adj) = 0.391   Deviance explained = 48.3%
-REML = 681.09 Scale est. = 0.2931   n = 774

14. Effects on *Euseius* spp. densities

> summary(*MarmaraDensity*)

Family: gaussian
Link function: identity

Formula:
gam(*MarmaraDensity* ~ s(*FieldSize*) + *CitrusSpecies* + *PlantingAge* + *CitrusMatrix* + *NaturalMatrix* + s(*Year*, bs = "re") + *RanchID* + s(*FieldID*, bs = "re") + s(*Longitude*, *Latitude*, bs = "tp", by = *Year*, k = 10), method = "REML", data = *CitrusFieldSize*)

Parametric coefficients:

	Estimate	Std. Error	t value	Pr(>|t|)
(Intercept)	6.177741	5.187892	1.191	0.23487
CitrusMatrix	0.741264	1.862407	0.398	0.69096
NaturalMatrix	0.421089	1.805297	0.233	0.81576
CitrusSpecies-limettioides	0.470187	0.675624	0.696	0.48713
CitrusSpecies-limon	0.282186	0.810273	0.348	0.72794
CitrusSpecies-maxima	-1.610793	0.650311	-2.477	0.01392 *
CitrusSpecies-meyeri	-0.019338	0.702156	-0.028	0.97805
CitrusSpecies-reticulata	0.381797	0.517465	0.738	0.46132
CitrusSpecies-sinensis	0.495282	0.397079	1.247	0.21346
CitrusSpecies-unshiu	0.123351	0.583538	0.211	0.83276
PlantingAge	0.010616	0.007472	1.421	0.15668
RanchID1	2.697513	0.853644	3.160	0.00177 **
RanchID2	16.332046	7.457314	2.190	0.02945 *
RanchID4	4.281249	2.292338	1.868	0.06299 .
RanchID5	3.980135	2.317104	1.718	0.08710 .
RanchID6	5.671479	3.305773	1.716	0.08748 .
RanchID7	1.587733	1.444360	1.099	0.27272
RanchID8	5.494568	3.381746	1.625	0.10548
RanchID9	-2.149105	4.517461	-0.476	0.63468
RanchID10	5.920822	3.126074	1.894	0.05939 .
RanchID11	4.322785	3.186099	1.357	0.17609

RanchID12	9.568801	4.251059	2.251	0.02527 *
RanchID13	6.455884	2.824251	2.286	0.02311 *
RanchID14	-29.912215	22.648967	-1.321	0.18782
RanchID15	-23.654377	16.635992	-1.422	0.15632
RanchID16	-22.837667	16.821653	-1.358	0.17581
RanchID17	9.406586	4.150973	2.266	0.02431 *
RanchID19	-7.927023	5.999869	-1.321	0.18765
RanchID20	-5.899913	3.621803	-1.629	0.10458
RanchID21	-7.943826	4.290462	-1.852	0.06529 .
RanchID22	-7.238685	8.504843	-0.851	0.39552

Signif. codes: 0 ‘***’ 0.001 ‘**’ 0.01 ‘*’ 0.05 ‘.’ 0.1 ‘ ’ 1

Approximate significance of smooth terms:

	edf	Ref.df	F	p-value
s(FieldSize)	1.000	1.001	1.542	0.215364
s(Year)	5.126	11.000	1.864	0.000118 ***
s(Longitude, Latitude):Year2003	2.000	2.000	1.824	0.163502
s(Longitude, Latitude):Year2004	3.003	3.425	1.222	0.244362
s(Longitude, Latitude):Year2005	2.000	2.000	1.898	0.152108
s(Longitude, Latitude):Year2006	2.986	3.475	1.637	0.154062
s(Longitude, Latitude):Year2007	3.223	3.897	1.731	0.163388
s(Longitude, Latitude):Year2008	3.726	4.488	2.262	0.055766 .
s(Longitude, Latitude):Year2009	2.894	3.156	1.450	0.278851
s(Longitude, Latitude):Year2010	7.842	8.621	5.702	9.16e-07 ***
s(Longitude, Latitude):Year2011	2.000	2.000	2.013	0.135706
s(Longitude, Latitude):Year2012	6.690	7.252	4.669	5.45e-05 ***
s(Longitude, Latitude):Year2013	2.000	2.000	1.758	0.174470
s(Longitude, Latitude):Year2017	2.840	3.100	1.627	0.164846
s(FieldID)	8.880	45.000	0.274	0.083633 .

Signif. codes: 0 ‘***’ 0.001 ‘**’ 0.01 ‘*’ 0.05 ‘.’ 0.1 ‘ ’ 1

R-sq.(adj) = 0.557   Deviance explained = 67.2%
-REML = 500.44 Scale est. = 1.1221   n = 335

15. Effects on citrus yield

> summary(Yield)

Family: gaussian
Link function: identity

Formula:
gam(Yield ~ s(*FieldSize*) + *CitrusSpecies* + *PlantingAge* + *CitrusMatrix* + *NaturalMatrix* + s(*Year*, bs = "re") + *RanchID* + s(*FieldID*, bs = "re") + s(*Longitude*, *Latitude*, bs = "tp", by = *Year*, k = 10), method = "REML", data = *CitrusFieldSize*)

Parametric coefficients:

	Estimate	Std. Error	t value	Pr(>|t|)
(Intercept)	46114.1	35138.3	1.312	0.18961
CitrusMatrix	9668.7	3651.3	2.648	0.00819 **
NaturalMatrix	1783.5	4898.7	0.364	0.71586
CitrusSpecies-clementina	-1239.3	8490.0	-0.146	0.88396
CitrusSpecies-clementina x sinensis	-18418.6	10192.6	-1.807	0.07097 .
CitrusSpecies-reticulata	9239.0	8426.7	1.096	0.27309
CitrusSpecies-sinensis	-2873.4	8660.1	-0.332	0.74009
CitrusSpecies-tangelo	-28398.0	10248.6	-2.771	0.00566 **
CitrusSpecies-unshiu	-3345.1	9545.6	-0.350	0.72606
RanchID2	9409.2	25141.6	0.374	0.70828
RanchID3	-7590.2	19752.4	-0.384	0.70084
RanchID5	-20913.2	13069.1	-1.600	0.10978
RanchID8	2187.9	12739.9	0.172	0.86367
RanchID12	928.4	16507.3	0.056	0.95516
RanchID13	10178.9	11195.1	0.909	0.36339
RanchID14	-44226.8	64171.3	-0.689	0.49081
RanchID15	-18378.4	49185.5	-0.374	0.70872
RanchID16	-27848.8	48974.5	-0.569	0.56969
RanchID17	32950.9	15329.2	2.150	0.03176 *
RanchID18	17320.1	15971.4	1.084	0.27836
RanchID19	-3819.9	18217.8	-0.210	0.83395
RanchID20	-1964.2	13592.4	-0.145	0.88512
RanchID21	12898.1	14550.6	0.886	0.37554
RanchID23	-47989.8	41571.9	-1.154	0.24854
RanchID24	-60630.9	50040.8	-1.212	0.22586
RanchID25	-46350.5	42118.3	-1.100	0.27131
RanchID26	-45392.9	49866.6	-0.910	0.36283
RanchID27	-35726.1	43567.8	-0.820	0.41235
RanchID29	-21317.4	47022.7	-0.453	0.65037
RanchID30	-24421.5	30912.7	-0.790	0.42965
RanchID31	-39949.8	44234.5	-0.903	0.36661
RanchID32	-20630.8	45070.4	-0.458	0.64721
RanchID33	-52235.1	45569.4	-1.146	0.25188
RanchID34	-30357.2	39849.0	-0.762	0.44630
RanchID36	-483.2	44938.4	-0.011	0.99142
RanchID37	-24678.8	47080.1	-0.524	0.60023

RanchID38	-45297.9	43097.8	-1.051	0.29342
RanchID39	-22161.1	55328.6	-0.401	0.68882
RanchID40	-31664.1	48423.2	-0.654	0.51328
RanchID41	-8879.1	18804.4	-0.472	0.63687
RanchID42	-25291.0	37440.4	-0.676	0.49947
RanchID43	-24537.3	29420.6	-0.834	0.40442
RanchID44	-46606.7	74188.9	-0.628	0.52996
RanchID45	-32312.8	32779.7	-0.986	0.32442
RanchID46	-17395.5	21286.7	-0.817	0.41395
RanchID47	-32027.0	36824.4	-0.870	0.38460
RanchID48	-6102.7	15291.1	-0.399	0.68988
RanchID49	-30940.9	38960.7	-0.794	0.42724
RanchID50	-38081.6	33513.3	-1.136	0.25602
RanchID51	-17080.9	29542.6	-0.578	0.56324
RanchID52	-43929.2	40724.7	-1.079	0.28091
RanchID53	-34554.4	29758.8	-1.161	0.24578
RanchID55	-30645.1	41833.2	-0.733	0.46395
RanchID56	-32406.6	46899.0	-0.691	0.48969
RanchID57	-41330.7	40499.9	-1.021	0.30766
RanchID58	-37519.6	39826.5	-0.942	0.34632
RanchID59	-39429.1	43268.7	-0.911	0.36232
RanchID60	-27384.1	34628.7	-0.791	0.42920
RanchID62	-35520.1	39708.6	-0.895	0.37120
RanchID63	-3298.1	16516.8	-0.200	0.84176
RanchID64	-46948.9	42184.4	-1.113	0.26592
RanchID65	-34003.8	31784.3	-1.070	0.28488
RanchID66	-28671.5	35489.3	-0.808	0.41929
RanchID67	-51312.5	39985.1	-1.283	0.19960
RanchID68	-11301.3	14106.2	-0.801	0.42317
RanchID69	-55388.1	48877.6	-1.133	0.25732
RanchID70	-16995.9	21183.2	-0.802	0.42250
RanchID71	-20982.7	48854.7	-0.429	0.66763
RanchID72	-32807.5	55000.4	-0.596	0.55094
RanchID73	-40238.0	39508.1	-1.018	0.30863
RanchID74	-17355.7	41639.6	-0.417	0.67688
RanchID75	-38727.1	31057.8	-1.247	0.21263
RanchID76	-8043.0	46603.1	-0.173	0.86300
RanchID77	5830.4	47209.3	0.124	0.90173
RanchID78	-9310.7	46737.9	-0.199	0.84213
RanchID79	8099.7	43993.8	0.184	0.85395
RanchID80	3166.3	43297.5	0.073	0.94171
RanchID81	-11284.5	45015.6	-0.251	0.80210
RanchID82	-41695.5	72731.9	-0.573	0.56655
RanchID83	-53581.6	48477.8	-1.105	0.26923

Signif. codes: 0 ‘***’ 0.001 ‘**’ 0.01 ‘*’ 0.05 ‘.’ 0.1 ‘ ’ 1

Approximate significance of smooth terms:

	edf	Ref.df	F	p-value
s(FieldSize)	1.003	1.004	4.491	0.0338 *
s(PlantingAge)	8.485	8.823	42.196	<2e-16 ***
s(Year)	10.895	15.000	7.816	<2e-16 ***
s(Longitude, Latitude):Year2003	2.006	2.011	1.001	0.3659
s(Longitude, Latitude):Year2004	2.003	2.006	0.922	0.3980
s(Longitude, Latitude):Year2005	2.001	2.002	0.778	0.4596
s(Longitude, Latitude):Year2006	2.002	2.004	0.964	0.3816
s(Longitude, Latitude):Year2007	4.483	5.753	1.578	0.1513
s(Longitude, Latitude):Year2008	2.001	2.002	0.895	0.4093
s(Longitude, Latitude):Year2009	2.002	2.004	1.049	0.3502
s(Longitude, Latitude):Year2010	2.421	2.785	0.917	0.4956
s(Longitude, Latitude):Year2011	2.001	2.002	0.921	0.3980
s(Longitude, Latitude):Year2012	4.958	6.125	0.864	0.4860
s(Longitude, Latitude):Year2013	6.052	7.293	2.191	0.0254 *
s(Longitude, Latitude):Year2014	6.101	7.334	2.279	0.0250 *
s(Longitude, Latitude):Year2015	6.669	7.797	1.863	0.0705 .
s(Longitude, Latitude):Year2016	6.767	7.880	2.429	0.0225 *
s(Longitude, Latitude):Year2017	3.360	3.754	0.841	0.4204
s(Longitude, Latitude):Year2018	2.001	2.001	0.430	0.6503
s(FieldID)	92.039	138.000	2.381	<2e-16 ***

Signif. codes: 0 ‘***’ 0.001 ‘**’ 0.01 ‘*’ 0.05 ‘.’ 0.1 ‘ ’ 1

R-sq.(adj) = 0.682   Deviance explained =   73%
-REML = 16539 Scale est. = 9.2657e+07 n = 1647

Table S9. Effects of field size on potato pest densities, targeted pesticide applications, and yield. Descriptions of GAM modeling results.

1. Effects on *Premnotrypes* spp. densities

> summary(*PremnotrypesDensity*)

Family: gaussian
Link function: identity

Formula:
PremnotrypesDensity ~ s(*FieldSize*) + *Cultivar* + *RotationCrop* + *PriorPotatoMatrix* + *Storage* + *CurrentPotatoMatrix* + s(Longitude_X, Latitude_Y, bs = "tp", k = 10) + *Observer*, method = "REML", data = *PotatoFieldSize*)

Parametric coefficients:

	Estimate	Std. Error	t value	Pr(>|t|)
(Intercept)	25.6029	4.2882	5.971	2.41e-08 ***
CultivarYungay	-3.1466	3.0300	-1.038	0.30111
RotationCropOther	3.4541	3.1924	1.082	0.28140
RotationCropPotato	13.7576	11.7805	1.168	0.24516
PriorPotatoMatrix	0.4388	0.3415	1.285	0.20132
Storage	3.9929	1.3891	2.875	0.00478 **
CurrentPotatoMatrix	-0.2832	0.1619	-1.750	0.08271 .
Observer1	-0.9207	4.2121	-0.219	0.82734
Observer2	-17.9912	4.2780	-4.206	5.01e-05 ***
Observer3	-8.7677	3.9700	-2.208	0.02909 *

Signif. codes: 0 ‘***’ 0.001 ‘**’ 0.01 ‘*’ 0.05 ‘.’ 0.1 ‘ ’ 1

Approximate significance of smooth terms:

	edf	Ref.df	F	p-value
s(FieldSize)	2.158	2.661	7.027	0.00043 ***
s(Longitude,Latitude)	4.302	5.596	2.295	0.05005 .

Signif. codes: 0 ‘***’ 0.001 ‘**’ 0.01 ‘*’ 0.05 ‘.’ 0.1 ‘ ’ 1

R-sq.(adj) = 0.336   Deviance explained = 41.1%
-REML = 549.38 Scale est. = 233.33   n = 138

2. Effects on insecticides targeting *Premnotrypes* spp.

summary(*PremnotrypesInsecticides*)

Family: gaussian
Link function: identity

Formula:
PremnotrypesInsecticides ~ s(*FieldSize*) + *Cultivar* + *RotationCrop* + *PriorPotatoMatrix* + *Storage* + *CurrentPotatoMatrix* + s(Longitude, Latitude, bs = "tp", k = 10) + *Observer*, method = "REML", data = PotatoFieldSize)

Parametric coefficients:

	Estimate	Std. Error	t value	Pr(>|t|)
(Intercept)	1.611909	0.248232	6.494	1.79e-09 ***
CultivarYungay	0.047949	0.178370	0.269	0.7885
RotationCropOther	0.096726	0.185305	0.522	0.6026
RotationCropPotato	0.133762	0.697771	0.192	0.8483
PriorPotatoMatrix	-0.035646	0.020084	-1.775	0.0784 .
Storage	0.168700	0.080844	2.087	0.0389 *
CurrentPotatoMatrix	0.016055	0.009215	1.742	0.0839 .
Observer1	0.250573	0.247982	1.010	0.3142
Observer2	0.223242	0.241653	0.924	0.3574
Observer3	0.119317	0.230365	0.518	0.6054

Signif. codes: 0 ‘***’ 0.001 ‘**’ 0.01 ‘*’ 0.05 ‘.’ 0.1 ‘ ’ 1

Approximate significance of smooth terms:

	edf	Ref.df	F	p-value
s(FieldSize)	1	1	0.679	0.412
s(Longitude,Latitude)	2	2	3.572	0.031 *

Signif. codes: 0 ‘***’ 0.001 ‘**’ 0.01 ‘*’ 0.05 ‘.’ 0.1 ‘ ’ 1

R-sq.(adj) = 0.0764 Deviance explained = 15.7%
-REML = 194.88 Scale est. = 0.83592 n = 138

3. Effects on *Epitrix* sp. densities

> summary(*EpitrixDensity*)

Family: gaussian
Link function: identity

Formula:
EpitrixDensity ~ s(*FieldSize*) + *Cultivar* + *RotationCrop* + *PriorPotatoMatrix* + *Storage* + *CurrentPotatoMatrix* + s(Longitude, Latitude, bs = "tp", k = 10) + *Observer*, method = "REML", data = PotatoFieldSize)

Parametric coefficients:

	Estimate	Std. Error	t value	Pr(>|t|)
(Intercept)	33.3992	5.3551	6.237	6.52e-09 ***
CultivarYungay	-9.6556	3.8277	-2.523	0.01293 *
RotationCropOther	4.0886	3.9974	1.023	0.30841
RotationCropPotato	9.0474	14.9410	0.606	0.54593
PriorPotatoMatrix	-0.1191	0.4309	-0.276	0.78269
Storage	0.6233	1.7371	0.359	0.72034
CurrentPotatoMatrix	-0.5229	0.1992	-2.625	0.00977 **
Observer1	1.2390	5.3198	0.233	0.81622
Observer2	-10.3160	5.2603	-1.961	0.05212 .
Observer3	2.6754	4.9604	0.539	0.59062

Signif. codes: 0 ‘***’ 0.001 ‘**’ 0.01 ‘*’ 0.05 ‘.’ 0.1 ‘ ’ 1

Approximate significance of smooth terms:

	edf	Ref.df	F	p-value
s(FieldSize)	2.130	2.628	4.81	0.0064 **
s(Longitude,Latitude)	2.665	3.218	1.31	0.2587

Signif. codes: 0 ‘***’ 0.001 ‘**’ 0.01 ‘*’ 0.05 ‘.’ 0.1 ‘ ’ 1

R-sq.(adj) = 0.21 Deviance explained = 29%
-REML = 578.47 Scale est. = 379.14 n = 138

4. Effects on potato yield

> summary(PotatoYield)

Family: gaussian
Link function: identity

Formula:

PotatoYield ~ s(*FieldSize*) + *Cultivar* + *RotationCrop* + *PriorPotatoMatrix* + *Storage* + *CurrentPotatoMatrix* + s(Longitude, Latitude, bs = "tp", k = 10) + *Observer*, method = "REML", data = PotatoFieldSize)

Parametric coefficients:

	Estimate	Std. Error	t value	Pr(>|t|)
(Intercept)	1.059513	0.245461	4.316	3.46e-05 ***
CultivarYungay	0.894762	0.177574	5.039	1.83e-06 ***
RotationCropOther	0.073836	0.187135	0.395	0.69392
RotationCropPotato	-1.981847	0.670441	-2.956	0.00381 **
PriorPotatoMatrix	0.018065	0.020443	0.884	0.37877
Storage	0.101277	0.079106	1.280	0.20312
CurrentPotatoMatrix	0.003204	0.009827	0.326	0.74504
Observer1	0.404583	0.255002	1.587	0.11545
Observer2	0.226437	0.240500	0.942	0.34848
Observer3	0.363932	0.231060	1.575	0.11809

Signif. codes: 0 ‘***’ 0.001 ‘**’ 0.01 ‘*’ 0.05 ‘.’ 0.1 ‘ ’ 1

Approximate significance of smooth terms:

	edf	Ref.df	F	p-value
s(FieldSize)	1.888	2.329	1.847	0.139
s(Longitude,Latitude)	2.000	2.000	0.214	0.808

R-sq.(adj) = 0.233   Deviance explained = 31.3%
-REML = 172.11 Scale est. = 0.75793   n = 125

Table S10. Effects of field size on grape pest densities and targeted pesticide applications. Descriptions of GAM modeling results.

1. Effects on *Lobesia botrana* densities

summary(*LobesiaDensity*)

Family: gaussian
Link function: identity

Formula:
LobesiaDensity ~ s(*FieldSize*) + *Cultivar* + *Irrigation* + *Altitude* + *GrapesMatrix* + *CerealsMatrix* + *OlivesMatrix* + *ShrubsMatrix* + *GrasslandMatrix* + *ForestMatrix* + s(*Year*, bs = "re") + *TechnicianID* + s(*FieldID*, bs = "re") + s(*Longitude*, *Latitude*, bs = "tp", by = *Year*, k = 10), method = "REML", data = *GrapesFieldSize*)

Parametric coefficients:

	Estimate	Std. Error	t value	Pr(>|t|)
(Intercept)	1.124485	0.671340	1.675	0.09437 .
CerealsMatrix	-0.202209	0.312417	-0.647	0.51768
OlivesMatrix	-0.377450	0.691644	-0.546	0.58542
GrapesMatrix	0.019473	0.341488	0.057	0.95454
ShrubsMatrix	-1.231503	1.517729	-0.811	0.41740
GrasslandMatrix	1.800200	3.012168	0.598	0.55027
ForestMatrix	-0.382654	1.358892	-0.282	0.77834
CultivarAiren	1.780103	0.594826	2.993	0.00286 **
CultivarCabernet	0.156472	0.573890	0.273	0.78520
CultivarChardonnay	-0.321943	1.096519	-0.294	0.76914
CultivarJaenblanco	-0.532149	1.264497	-0.421	0.67400
CultivarMerlot	0.030369	0.692056	0.044	0.96501
CultivarMoscatel	0.194759	0.290998	0.669	0.50353
CultivarPalomino	0.176283	0.249661	0.706	0.48036
CultivarPedroximenez	-0.227720	0.298034	-0.764	0.44507
CultivarSYearah	-0.206984	0.332748	-0.622	0.53411
CultivarTempranillo	-0.076785	0.455621	-0.169	0.86622
CultivarVerdejo	-0.435980	0.640686	-0.680	0.49641
CultivarZalema	-0.176292	0.473413	-0.372	0.70971
technicianasp	-0.225934	0.356905	-0.633	0.52691
technicianbpp	-0.318663	0.252824	-1.260	0.20793
techniciancrg	0.177134	1.020846	0.174	0.86229

techniciandc	-0.428407	1.019886	-0.420	0.67457
technicianddb	-0.531658	0.968928	-0.549	0.58338
technicianegg	-0.405571	1.097376	-0.370	0.71180
technicianegm	-0.137214	1.032777	-0.133	0.89434
technicianela	-0.755823	0.817431	-0.925	0.35546
technicianera	-0.276714	0.492688	-0.562	0.57453
technicianeso	-1.211012	0.861309	-1.406	0.16015
technicianfgd	-0.064010	0.967377	-0.066	0.94726
technicianilh	-1.691238	1.150165	-1.470	0.14188
technicianja	-0.234368	1.070020	-0.219	0.82669
technicianjaj	0.553802	1.066780	0.519	0.60383
technicianjap	-0.805751	1.000804	-0.805	0.42102
technicianjbl	-0.388737	0.290280	-1.339	0.18093
technicianjbr	-0.299470	0.290712	-1.030	0.30329
technicianjcb	-0.048541	0.459949	-0.106	0.91598
technicianjcp	-0.845405	0.728696	-1.160	0.24637
technicianjhp	-0.653121	0.315896	-2.068	0.03904 *
technicianjmg	-0.101914	0.277821	-0.367	0.71385
technicianjms	0.288947	0.226241	1.277	0.20195
technicianjnb	0.110878	0.393112	0.282	0.77798
technicianjpl	-1.956542	1.353706	-1.445	0.14880
technicianjpm	-0.910595	0.875464	-1.040	0.29863
technicianjpp	-0.334581	0.492579	-0.679	0.49720
technicianjr	2.614338	2.426855	1.077	0.28173
technicianjrl	-1.646179	0.908811	-1.811	0.07050 .
technicianjrp	0.932211	0.804442	1.159	0.24691
technicianjzm	1.831844	0.861956	2.125	0.03391 *
technicianlda	0.183291	0.291126	0.630	0.52916
technicianlmc	-1.145133	1.077071	-1.063	0.28805
technicianlpp	-0.273829	0.639820	-0.428	0.66879
technicianlpr	-0.792003	1.033716	-0.766	0.44382
technicianlps	0.980044	0.505582	1.938	0.05296 .
technicianmcg	-0.496706	0.415332	-1.196	0.23212
technicianmcp	-0.346160	0.475996	-0.727	0.46732
technicianmjp	-1.399892	1.160487	-1.206	0.22810
technicianmlb	-1.814646	1.056512	-1.718	0.08630 .
technicianmlp	-0.473078	1.285972	-0.368	0.71307
technicianmlr	-0.839517	1.154249	-0.727	0.46726
technicianmvc	0.790925	1.338944	0.591	0.55490
technicianmvd	-0.127454	0.324616	-0.393	0.69471
technicianmvp	-0.705504	0.521741	-1.352	0.17673
technicianpcr	-0.886602	0.744259	-1.191	0.23394
technicianprc	-0.764099	0.373274	-2.047	0.04102 *
technicianrhm	-0.369726	0.423321	-0.873	0.38274

technicianrmg	-0.621904	0.362040	-1.718	0.08626 .
technicianrmm	-0.210709	1.233628	-0.171	0.86443
technicianrsg	-1.068836	1.134289	-0.942	0.34635
techniciansgg	-0.325387	0.335193	-0.971	0.33200
technicianssr	0.625213	1.885063	0.332	0.74024
technicianuk	0.506845	0.604960	0.838	0.40241
technicianvn	-1.139552	0.766669	-1.486	0.13762
technicianvnh	-0.927273	0.768862	-1.206	0.22820
irrigationuk	-0.091341	0.150187	-0.608	0.54326
irrigationwater	-0.293358	0.233975	-1.254	0.21032
Altitude	0.000657	0.001223	0.537	0.59115

Signif. codes: 0 ‘***’ 0.001 ‘**’ 0.01 ‘*’ 0.05 ‘.’ 0.1 ‘ ’ 1

Approximate significance of smooth terms:

	edf	Ref.df	F	p-value
s(FieldSize)	1.021	1.036	0.024	0.93778
s(Year)	11.667	12.000	12.929	< 2e-16 ***
s(FieldID)	132.558	358.000	0.915	< 2e-16 ***
s(Longitude,Latitude):Year2006	2.000	2.000	0.951	0.38671
s(Longitude,Latitude):Year2007	2.000	2.001	3.912	0.02042 *
s(Longitude,Latitude):Year2008	2.301	2.539	0.888	0.39109
s(Longitude,Latitude):Year2009	5.178	6.263	3.222	0.00405 **
s(Longitude,Latitude):Year2010	4.795	5.726	2.988	0.00655 **
s(Longitude,Latitude):Year2011	5.214	6.155	5.110	3.36e-05 ***
s(Longitude,Latitude):Year2012	2.000	2.001	1.954	0.14239
s(Longitude,Latitude):Year2013	2.000	2.000	2.766	0.06356 .
s(Longitude,Latitude):Year2014	6.208	7.018	5.481	3.61e-06 ***
s(Longitude,Latitude):Year2015	4.159	4.975	2.460	0.02647 *
s(Longitude,Latitude):Year2016	2.003	2.005	1.997	0.13665
s(Longitude,Latitude):Year2017	8.531	8.847	67.915	< 2e-16 ***
s(Longitude,Latitude):Year2018	2.000	2.000	2.659	0.07072 .

Signif. codes: 0 ‘***’ 0.001 ‘**’ 0.01 ‘*’ 0.05 ‘.’ 0.1 ‘ ’ 1

R-sq.(adj) = 0.675   Deviance explained = 76.3%
-REML = 1442.8 Scale est. = 0.77447   n = 996

2. Effects on insecticides targeting *Lobesia botrana*

summary(*LobesiaInsecticides*)

Family: gaussian

Link function: identity

Formula:
LobesiaInsecticides ~ s(*FieldSize*) + *Cultivar* + + *Irrigation* + *Altitude* + *GrapesMatrix* + *CerealsMatrix* + *OlivesMatrix* + *ShrubsMatrix* + *GrasslandMatrix* + *ForestMatrix* + s(*Year*, bs = "re") + *TechnicianID* + s(*FieldID*, bs = "re") + s(*Longitude*, *Latitude*, bs = "tp", by = *Year*, k = 10), method = "REML", data = *GrapesFieldSize*)

Parametric coefficients:

	Estimate	Std. Error	z value	Pr(>|z|)
(Intercept)	-1.331e+00	5.739e-01	-2.318	0.02042 *
CerealsMatrix	3.135e-01	3.275e-01	0.957	0.33843
OlivesMatrix	7.019e-01	1.065e+00	0.659	0.50977
GrapesMatrix	9.803e-01	3.417e-01	2.869	0.00412 **
ShrubsMatrix	1.804e+00	2.482e+00	0.727	0.46748
GrasslandMatrix	2.751e-01	2.527e+00	0.109	0.91334
ForestMatrix	1.983e-01	1.964e+00	0.101	0.91958
cultivarairen	-6.410e+01	3.905e+07	0.000	1.00000
cultivarcabernet	1.888e+00	4.482e-01	4.212	2.53e-05 ***
CultivarChardonnay	-7.990e+01	6.711e+07	0.000	1.00000
CultivarJaenblanco	-3.042e-01	1.319e+00	-0.231	0.81754
CultivarMerlot	-1.804e-01	1.290e+00	-0.140	0.88876
CultivarMoscatel	-7.464e-01	4.119e-01	-1.812	0.06997 .
CultivarPalomino	-2.748e-03	2.027e-01	-0.014	0.98918
CultivarPedroximenez	1.932e-03	5.645e-01	0.003	0.99727
CultivarSYearah	5.753e-02	4.595e-01	0.125	0.90037
CultivarTempranillo	-1.097e-01	5.795e-01	-0.189	0.84981
CultivarVerdejo	-5.765e+01	3.487e+07	0.000	1.00000
CultivarZalema	-5.099e-01	6.554e-01	-0.778	0.43660
technicianasp	-1.078e+00	3.873e-01	-2.782	0.00540 **
technicianbpp	1.056e-02	1.568e-01	0.067	0.94631
techniciancrg	-1.931e+00	5.720e+00	-0.338	0.73574
technicianddb	-8.131e+01	4.745e+07	0.000	1.00000
technicianegg	4.797e+00	1.777e+00	2.699	0.00695 **
technicianegm	-8.029e+01	4.745e+07	0.000	1.00000
technicianela	-3.696e-01	1.149e+00	-0.322	0.74776
technicianera	-1.082e+00	7.433e-01	-1.456	0.14543
technicianeso	3.802e+00	2.079e+00	1.829	0.06747 .
technicianfgd	2.211e+00	1.731e+00	1.278	0.20139
technicianilh	-6.918e+01	3.467e+07	0.000	1.00000
technicianja	4.284e+00	1.662e+00	2.578	0.00994 **
technicianjaj	4.447e+00	1.686e+00	2.638	0.00835 **
technicianjap	-7.775e+01	3.875e+07	0.000	1.00000

technicianjbl	-4.906e-01	2.791e-01	-1.758	0.07881 .
technicianjbr	9.983e-02	1.828e-01	0.546	0.58493
technicianjcb	-1.955e-01	3.584e-01	-0.545	0.58546
technicianjcp	-8.072e+01	3.001e+07	0.000	1.00000
technicianjhp	-3.144e+00	1.022e+00	-3.076	0.00209 **
technicianjmg	-3.746e-01	2.254e-01 -	1.662	0.09647 .
technicianjms	7.613e-02	1.505e-01	0.506	0.61287
technicianjnb	-8.034e+01	3.001e+07	0.000	1.00000
technicianjpl	-7.542e+01	4.745e+07	0.000	1.00000
technicianjpm	-7.959e+01	1.678e+07	0.000	1.00000
technicianjpp	-1.133e+00	5.530e-01	-2.049	0.04042 *
technicianjr	-7.294e+01	2.373e+07	0.000	1.00000
technicianjrl	-7.586e+01	2.122e+07	0.000	1.00000
technicianjrp	-7.587e+01	1.451e+07	0.000	1.00000
technicianjzm	2.931e+00	1.492e+00	1.964	0.04953 *
technicianlda	-3.353e-02	2.076e-01	-0.162	0.87167
technicianlmc	-8.093e+01	6.711e+07	0.000	1.00000
technicianlpp	-1.119e+00	7.217e-01	-1.551	0.12095
technicianlps	-4.271e-01	5.685e-01	-0.751	0.45252
technicianmcg	1.891e-01	3.125e-01	0.605	0.54501
technicianmcp	-2.918e-01	4.617e-01	-0.632	0.52738
technicianmjp	-7.439e+01	2.740e+07	0.000	1.00000
technicianmlb	2.883e+00	1.748e+00	1.650	0.09902 .
technicianmlp	-8.060e+01	6.711e+07	0.000	1.00000
technicianmlr	-8.131e+01	4.745e+07	0.000	1.00000
technicianmvd	-2.607e-01	2.900e-01	-0.899	0.36879
technicianmvp	1.278e-01	3.734e-01	0.342	0.73223
technicianpcr	-5.773e+00	2.003e+00	-2.882	0.00395 **
technicianprc	-3.581e-01	2.272e-01	-1.576	0.11492
technicianrhm	-1.226e+00	5.191e-01	-2.363	0.01813 *
technicianrmg	-1.189e+00	3.658e-01	-3.249	0.00116 **
technicianrmm	-7.814e+01	3.001e+07	0.000	1.00000
technicianrsg	-7.948e+01	4.745e+07	0.000	1.00000
techniciansgg	-2.199e-01	2.283e-01	-0.963	0.33535
technicianssr	-7.539e+01	2.536e+07	0.000	1.00000
technicianuk	-1.025e-01	7.287e-01	-0.141	0.88816
technicianvn	-4.243e+00	1.815e+00	-2.338	0.01938 *
technicianvnh	-1.414e+00	1.006e+00	-1.405	0.16015
irrigationuk	8.693e-02	1.511e-01	0.575	0.56499
irrigationwater	1.593e-01	2.766e-01	0.576	0.56464
Altitude	-5.688e-04	1.912e-03	-0.297	0.76615

Signif. codes: 0 ‘***’ 0.001 ‘**’ 0.01 ‘*’ 0.05 ‘.’ 0.1 ‘ ’ 1

Approximate significance of smooth terms:

	edf	Ref.df	Chi.sq	p-value
s(FieldSize)	1.000016	1.000	0.039	0.843157
s(Year)	4.705303	12.000	17.052	0.000646 ***
s(FieldID)	0.001035	303.000	0.001	0.757936
s(Longitude,Latitude):Year2006	4.117868	5.091	11.180	0.053563 .
s(Longitude,Latitude):Year2007	2.000015	2.000	7.503	0.023481 *
s(Longitude,Latitude):Year2008	2.000041	2.000	11.419	0.003315 **
s(Longitude,Latitude):Year2009	2.000054	2.000	14.690	0.000646 ***
s(Longitude,Latitude):Year2010	6.041547	7.006	21.352	0.003656 **
s(Longitude,Latitude):Year2011	2.000029	2.000	21.801	1.93e-05 ***
s(Longitude,Latitude):Year2012	2.000118	2.000	12.030	0.002444 **
s(Longitude,Latitude):Year2013	2.000237	2.000	15.988	0.000339 ***
s(Longitude,Latitude):Year2014	2.204491	2.359	17.621	0.000260 ***
s(Longitude,Latitude):Year2015	2.000027	2.000	11.704	0.002874 **
s(Longitude,Latitude):Year2016	4.902344	5.486	22.094	0.001007 **
s(Longitude,Latitude):Year2017	5.085647	6.034	4.555	0.604869
s(Longitude,Latitude):Year2018	4.531743	5.199	14.437	0.013472 *

Signif. codes: 0 ‘***’ 0.001 ‘**’ 0.01 ‘*’ 0.05 ‘.’ 0.1 ‘ ’ 1

R-sq.(adj) = 0.519   Deviance explained = 58.6%
-REML = 504.87 Scale est. = 1   n = 929

3. Effects on *Jacobiasca* sp. densities

summary(*JacobiascaInsecticides*)

Family: gaussian
Link function: identity

Formula:
JacobiascaDensity ~ s(*FieldSize*) + *Cultivar* + + *Irrigation* + *Altitude* + + *Irrigation* + *Altitude* + *GrapesMatrix* + *CerealsMatrix* + *OlivesMatrix* + *ShrubsMatrix* + *GrasslandMatrix* + *ForestMatrix* + s(*Year*, bs = "re") + *TechnicianID* + s(*FieldID*, bs = "re") + s(*Longitude*, *Latitude*, bs = "tp", by = *Year*, k = 10), method = "REML", data = *GrapesFieldSize*)

Parametric coefficients:

	Estimate	Std. Error	t value	Pr(>|t|)
(Intercept)	0.4036779	0.2122976	1.901	0.057531 .
CerealsMatrix	0.0906976	0.1439246	0.630	0.528726
OlivesMatrix	-0.1255426	0.2923785	-0.429	0.667738

GrapesMatrix	-0.0663977	0.1519417	-0.437	0.662211
ShrubsMatrix	-0.0626019	0.6542702	-0.096	0.923792
GrasslandMatrix	4.2544623	1.1568778	3.678	0.000248 ***
ForestMatrix	-0.3684403	0.5623503	-0.655	0.512505
CultivarAiren	0.0559116	0.2491113	0.224	0.822458
cultivarcabernet	-0.0978992	0.2556960	-0.383	0.701896
CultivarChardonnay	-0.1203986	0.5791700	-0.208	0.835364
CultivarJaenblanco	-0.1813317	0.6684373	-0.271	0.786235
CultivarMerlot	-0.0089081	0.2937238	-0.030	0.975812
CultivarMoscatel	-0.0363868	0.1167349	-0.312	0.755331
CultivarPalomino	0.0396912	0.1094193	0.363	0.716873
CultivarPedroximenez	-0.0272626	0.1249128	-0.218	0.827277
CultivarSYearah	-0.0424096	0.1395478	-0.304	0.761262
CultivarTempranillo	-0.0194849	0.2235634	-0.087	0.930565
CultivarVerdejo	-0.1750033	0.2679383	-0.653	0.513813
CultivarZalema	-0.0286119	0.1979833	-0.145	0.885122
technicianasp	-0.1285412	0.1630048	-0.789	0.430551
technicianbpp	0.0840420	0.0964230	0.872	0.383640
techniciancrg	-0.4027395	0.4684831	-0.860	0.390181
techniciandc	0.0214038	0.5876977	0.036	0.970955
technicianddb	0.4394556	0.4945833	0.889	0.374468
technicianegg	-0.5894407	0.4918430	-1.198	0.231036
technicianegm	-0.1550611	0.4061946	-0.382	0.702736
technicianela	0.3943896	0.3568376	1.105	0.269327
technicianera	0.1064795	0.2467229	0.432	0.666144
technicianeso	-0.1435425	0.4133085	-0.347	0.728439
technicianfgd	1.4433270	0.4831646	2.987	0.002885 **
technicianilh	-0.5361720	0.5456275	-0.983	0.326010
technicianja	-0.6620534	0.4737904	-1.397	0.162621
technicianjaj	-0.3351916	0.4768562	-0.703	0.482271
technicianjap	3.5213580	0.4930780	7.142	1.79e-12 ***
technicianjbl	0.1589069	0.1255848	1.265	0.206050
technicianjbr	-0.1926829	0.1159265	-1.662	0.096807 .
technicianjcb	-0.1625845	0.1621009	-1.003	0.316114
technicianjcp	-1.0196805	0.3068298	-3.323	0.000922 ***
technicianjhp	0.1104691	0.1464547	0.754	0.450856
technicianjmg	0.0144475	0.1227476	0.118	0.906329
technicianjms	0.1685661	0.0927693	1.817	0.069513 .
technicianjnb	-0.3546005	0.1975534	-1.795	0.072966 .
technicianjpl	-0.6555339	0.6261839	-1.047	0.295415
technicianjpm	0.0956025	0.3822900	0.250	0.802579
technicianjpp	0.3699080	0.2625149	1.409	0.159122
technicianjr	-0.4995582	0.5058352	-0.988	0.323595
technicianjrl	-0.1048553	0.4222097	-0.248	0.803916

technicianjrp	-0.4160755	0.3835580	-1.085	0.278284
technicianjzm	-0.2492117	0.3868925	-0.644	0.519636
technicianlda	0.2805909	0.1255689	2.235	0.025669 *
technicianlmc	0.0664059	0.5647603	0.118	0.906422
technicianlpp	0.1793154	0.3341988	0.537	0.591697
technicianlpr	0.1653539	0.4712041	0.351	0.725725
technicianlps	1.0205856	0.3012404	3.388	0.000732 ***
technicianmcg	-0.7712046	0.2336961	-3.300	0.001001 **
technicianmcp	0.1891892	0.2498684	0.757	0.449137
technicianmlb	-0.3415235	0.4862867	-0.702	0.482652
technicianmlp	-0.8144773	0.7300069	-1.116	0.264817
technicianmlr	-1.0747171	0.5295174	-2.030	0.042663 *
technicianmvc	-0.6132384	0.6508189	-0.942	0.346292
technicianmvd	0.2720841	0.1584393	1.717	0.086242 .
technicianpcr	0.6419144	0.3188076	2.013	0.044334 *
technicianprc	0.3104948	0.1401616	2.215	0.026969 *
technicianrhm	-0.1908516	0.1947138	-0.980	0.327245
technicianrmg	0.0295082	0.1561447	0.189	0.850148
technicianrmm	0.1467341	0.4449136	0.330	0.741618
technicianrsg	-0.2161968	0.5356227	-0.404	0.686567
techniciansgg	0.0093202	0.1305872	0.071	0.943117
technicianssr	-0.1998726	0.4736599	-0.422	0.673135
technicianuk	-0.0207335	0.3212933	-0.065	0.948560
technicianvn	0.1036844	0.3037058	0.341	0.732877
technicianvnh	0.4313315	0.3335152	1.293	0.196213
irrigationuk	-0.0099044	0.0668131	-0.148	0.882183
irrigationwater	0.1422870	0.0898519	1.584	0.113611
Altitude	-0.0008572	0.0004675	-1.834	0.067020 .

Signif. codes: 0 ‘***’ 0.001 ‘**’ 0.01 ‘*’ 0.05 ‘.’ 0.1 ‘ ’ 1

Approximate significance of smooth terms:

	edf	Ref.df	F	p-value
s(FieldSize)	1.000	1.000	0.339	0.560649
s(Year)	9.087	12.000	2.305	0.000312 ***
s(FieldID)	10.440	365.000	0.030	0.320382
s(Longitude,Latitude):Year2006	4.932	6.085	0.604	0.624314
s(Longitude,Latitude):Year2007	2.000	2.000	0.548	0.578510
s(Longitude,Latitude):Year2008	2.000	2.000	3.056	0.047537 *
s(Longitude,Latitude):Year2009	2.000	2.000	2.359	0.095045 .
s(Longitude,Latitude):Year2010	2.000	2.000	2.156	0.116285
s(Longitude,Latitude):Year2011	2.000	2.000	1.647	0.193086
s(Longitude,Latitude):Year2012	2.002	2.004	1.608	0.200608
s(Longitude,Latitude):Year2013	2.000	2.000	2.847	0.058484 .

s(Longitude,Latitude):Year2014	2.000	2.000	1.616	0.199301
s(Longitude,Latitude):Year2015	2.461	2.816	3.326	0.024259 *
s(Longitude,Latitude):Year2016	2.000	2.000	7.433	0.000625 ***
s(Longitude,Latitude):Year2017	2.000	2.000	3.074	0.046665 *
s(Longitude,Latitude):Year2018	2.000	2.000	2.015	0.133867

Signif. codes: 0 ‘***’ 0.001 ‘**’ 0.01 ‘*’ 0.05 ‘.’ 0.1 ‘ ’ 1

R-sq.(adj) = 0.323   Deviance explained = 39.8%
-REML = 992.53 Scale est. = 0.30349   n = 1113

4. Effects on insecticides targeting *Jacobiasca sp.*

summary(*JacobiascaInsecticides*)

Family: gaussian
Link function: identity

Formula:
JacobiascaInsecticides ~ s(*FieldSize*) + *Cultivar* + + *Irrigation* + *Altitude* + *GrapesMatrix* + *CerealsMatrix* + *OlivesMatrix* + *ShrubsMatrix* + *GrasslandMatrix* + *ForestMatrix* + s(*Year*, bs = "re") + *TechnicianID* + s(*FieldID*, bs = "re") + s(*Longitude*, *Latitude*, bs = "tp", by = *Year*, k = 10), method = "REML", data = *GrapesFieldSize*)

Family: poisson
Link function: log

Parametric coefficients:

	Estimate	Std. Error	z value	Pr(>|z|)
(Intercept)	-2.021e+00	8.782e-01	-2.301	0.02139 *
CerealsMatrix	3.866e-01	5.944e-01	0.650	0.51541
OlivesMatrix	-1.463e-02	1.148e+00	-0.013	0.98983
GrapesMatrix	-3.294e-01	6.203e-01	-0.531	0.59539
ShrubsMatrix	1.269e+00	2.267e+00	0.560	0.57545
GrasslandMatrix	-1.531e+00	3.331e+00	-0.459	0.64588
ForestMatrix	1.256e+00	1.526e+00	0.823	0.41036
CultivarAiren	5.090e-01	9.244e-01	0.551	0.58186
cultivarcabernet	1.424e+00	9.245e-01	1.541	0.12337
CultivarChardonnay	2.348e+00	1.371e+00	1.713	0.08679 .
CultivarJaenblanco	2.127e-01	7.749e+07	0.000	1.00000
CultivarMerlot	1.246e+00	9.889e-01	1.260	0.20778

CultivarMoscatel	2.237e-01	5.557e-01	0.403	0.68723
CultivarPalomino	1.167e+00	5.648e-01	2.066	0.03882 *
CultivarPedroximenez	-3.506e-01	5.542e-01	-0.633	0.52702
CultivarSYearah	4.813e-01	5.719e-01	0.842	0.40000
CultivarTempranillo	1.967e+00	7.445e-01	2.642	0.00825 **
CultivarVerdejo	-7.636e+01	3.355e+07	0.000	1.00000
CultivarZalema	6.261e-01	6.034e-01	1.038	0.29940
technicianasp	-4.508e-02	4.658e-01	-0.097	0.92289
technicianbpp	1.212e-02	2.412e-01	0.050	0.95991
techniciancrg	-1.680e+00	2.306e+00	-0.728	0.46641
technicianddb	-7.595e+01	4.745e+07	0.000	1.00000
technicianegg	-3.204e+00	2.310e+00	-1.387	0.16545
technicianegm	1.261e+00	5.748e-01	2.195	0.02819 *
technicianela	-4.801e-03	1.541e+00	-0.003	0.99751
technicianera	-4.023e-01	1.255e+00	-0.321	0.74844
technicianeso	-7.736e+01	2.237e+07	0.000	1.00000
technicianfgd	-7.793e+01	3.875e+07	0.000	1.00000
technicianilh	-1.011e+00	3.183e+00	-0.318	0.75069
technicianja	-4.660e+00	2.359e+00	-1.975	0.04823 *
technicianjaj	-5.113e+00	2.408e+00	-2.123	0.03375 *
technicianjap	-2.212e+00	2.031e+00	-1.089	0.27609
technicianjbl	8.183e-01	4.746e-01	1.724	0.08467 .
technicianjbr	7.752e-02	4.633e-01	0.167	0.86711
technicianjcb	-9.439e-01	5.814e-01	-1.623	0.10450
technicianjcp	1.687e+00	7.032e-01	2.399	0.01643 *
technicianjhp	-1.436e+00	8.489e-01	-1.692	0.09064 .
technicianjmg	9.102e-01	5.184e-01	1.756	0.07909 .
technicianjms	-2.165e-01	2.910e-01	-0.744	0.45693
technicianjnb	-7.605e+01	3.001e+07	0.000	1.00000
technicianjpl	-2.389e+00	2.545e+00	-0.939	0.34789
technicianjpm	-2.414e+00	1.405e+00	-1.718	0.08576 .
technicianjpp	9.057e-01	9.555e-01	0.948	0.34315
technicianjr	-7.637e+01	2.373e+07	0.000	1.00000
technicianjrl	-2.120e+00	1.998e+00	-1.061	0.28859
technicianjrp	-4.511e+00	2.843e+00	-1.587	0.11254
technicianjzm	-2.319e+00	1.702e+00	-1.362	0.17323
technicianlda	-7.503e+01	1.186e+07	0.000	0.99999
technicianlmc	-7.550e+01	6.711e+07	0.000	1.00000
technicianlpp	-7.304e+01	3.875e+07	0.000	1.00000
technicianlps	-7.705e+01	2.122e+07	0.000	1.00000
technicianmcg	-7.468e+01	2.122e+07	0.000	1.00000
technicianmcp	-3.012e-01	6.513e-01	-0.463	0.64370
technicianmjp	-7.730e+01	2.740e+07	0.000	1.00000
technicianmlb	-4.972e+00	2.431e+00	-2.045	0.04085 *

technicianmlp	-7.820e+01	6.711e+07	0.000	1.00000
technicianmlr	-7.808e+01	4.745e+07	0.000	1.00000
technicianmvd	3.098e-01	4.967e-01	0.624	0.53284
technicianmvp	-7.556e+01	3.001e+07	0.000	1.00000
technicianpcr	-1.260e+00	1.496e+00	-0.842	0.39968
technicianprc	5.613e-02	4.984e-01	0.113	0.91032
technicianrhm	-7.621e+01	1.937e+07	0.000	1.00000
technicianrmg	4.857e-01	7.024e-01	0.691	0.48930
technicianrmm	-7.618e+01	3.001e+07	0.000	1.00000
technicianrsg	-7.567e+01	4.745e+07	0.000	1.00000
techniciansgg	-3.805e-01	6.901e-01	-0.551	0.58137
technicianssr	-2.163e+00	5.003e+00	-0.432	0.66552
technicianuk	1.089e-01	8.358e-01	0.130	0.89632
technicianvn	4.003e-01	1.497e+00	0.267	0.78918
technicianvnh	-1.582e+00	1.556e+00	-1.017	0.30935
irrigationuk	-5.714e-01	3.166e-01	-1.805	0.07106 .
irrigationwater	-2.503e-04	4.101e-01	-0.001	0.99951
Altitude	1.190e-03	1.595e-03	0.746	0.45577

Signif. codes: 0 ‘***’ 0.001 ‘**’ 0.01 ‘*’ 0.05 ‘.’ 0.1 ‘ ’ 1

Approximate significance of smooth terms:

	edf	Ref.df	Chi.sq	p-value
s(FieldSize)	1.000	1.000	0.019	0.88949
s(Year)	8.342	12.000	36.266	3.97e-06 ***
s(FieldID)	4.911	298.000	4.967	0.42280
s(Longitude,Latitude):Year2006	5.552	6.581	16.373	0.01640 *
s(Longitude,Latitude):Year2007	4.403	5.509	8.239	0.16466
s(Longitude,Latitude):Year2008	2.000	2.000	7.576	0.02265 *
s(Longitude,Latitude):Year2009	5.133	6.128	20.483	0.00337 **
s(Longitude,Latitude):Year2010	4.163	4.824	15.464	0.00999 **
s(Longitude,Latitude):Year2011	2.000	2.000	5.021	0.08127 .
s(Longitude,Latitude):Year2012	2.000	2.001	8.893	0.01173 *
s(Longitude,Latitude):Year2013	3.233	3.912	1.904	0.76236
s(Longitude,Latitude):Year2014	2.000	2.000	5.639	0.05963 .
s(Longitude,Latitude):Year2015	3.383	4.027	3.190	0.50604
s(Longitude,Latitude):Year2016	2.000	2.000	1.064	0.58757
s(Longitude,Latitude):Year2017	2.000	2.000	1.513	0.46942
s(Longitude,Latitude):Year2018	2.000	2.000	0.883	0.64324

Signif. codes: 0 ‘***’ 0.001 ‘**’ 0.01 ‘*’ 0.05 ‘.’ 0.1 ‘ ’ 1

R-sq.(adj) =   0.44   Deviance explained = 59.8%
-REML = 173.66 Scale est. = 1   n = 929

Table S11. Effects of field size on olive pest densities and targeted pesticide applications. Descriptions of GAM modeling results.

1. Effects on *Bactrocera oleae* densities

> summary(BactroceraDensity)

Family: gaussian
Link function: identity

Formula:
BactroceraDensity ~ s(*FieldSize*) + *Cultivar* + *Altitude*+ *Irrigation*+ *OlivesMatrix* + *ShrubsMatrix* + *GrasslandMatrix* + *ForestMatrix* + s(*Year*, bs = "re") + *TechnicianID* + s(*FieldID*, bs = "re") + s(xutm, yutm, bs = "tp", by = *Year*, k = 10)

Parametric coefficients:

	Estimate	Std. Error	t value	Pr(>|t|)
(Intercept)	0.8945765	0.4799628	1.864	0.062364 .
OlivesMatrix	0.6352541	0.1275457	4.981	6.41e-07 ***
ShrubsMatrix	0.7276278	0.2790023	2.608	0.009118 **
GrasslandMatrix	1.3226673	0.4761280	2.778	0.005477 **
ForestMatrix	0.5607636	0.3265723	1.717	0.085979 .
Cultivar.alo	2.6815739	0.9346335	2.869	0.004122 **
Cultivar.arbe	-0.2649031	0.1557344	-1.701	0.088966 .
Cultivar.corni	0.7502656	0.7473369	1.004	0.315435
Cultivar.gordal	-0.3535700	0.2428298	-1.456	0.145403
Cultivar.hoji	-0.3100016	0.0977411	-3.172	0.001519 **
Cultivar.lechin	0.4189037	0.1712966	2.445	0.014477 *
Cultivar.lucio	-0.0261407	0.6058432	-0.043	0.965584
Cultivar.manz	0.0535558	0.1334632	0.401	0.688221
Cultivar.morona	-0.3459738	0.5663341	-0.611	0.541275
Cultivar.nevadillo	-0.5185934	0.3368315	-1.540	0.123674
Cultivar.picolimon	0.2893883	0.4851395	0.597	0.550847
Cultivar.picual	-0.0946646	0.0864853	-1.095	0.273721
Cultivar.picudo	0.3175151	0.2232661	1.422	0.155008
Cultivar.verdial	0.6619827	0.2749785	2.407	0.016079 *
TechnicianID.aagm	-0.6684928	0.4632062	-1.443	0.148990
TechnicianID.aal	1.0970073	0.5184308	2.116	0.034361 *
TechnicianID.abta	0.5245127	0.6669063	0.786	0.431595
TechnicianID.acl	-0.9784616	0.5277955	-1.854	0.063779 .
TechnicianID.acp	0.1974867	0.5229939	0.378	0.705727

TechnicianID.acs	-0.3808805	0.4969546	-0.766	0.443434
TechnicianID.agam	0.3995160	0.4586909	0.871	0.383773
TechnicianID.agl	1.4086811	0.7046700	1.999	0.045620 *
TechnicianID.agr	1.3167675	0.4389888	3.000	0.002708 **
TechnicianID.agr2	3.1713512	0.4418160	7.178	7.42e-13 ***
TechnicianID.ahm	-0.5071033	0.4691505	-1.081	0.279761
TechnicianID.ahs	1.4924900	0.4722591	3.160	0.001579 **
TechnicianID.ajbm2	2.3442478	0.6280821	3.732	0.000190 ***
TechnicianID.ajbr	4.6448402	0.5446047	8.529	< 2e-16 ***
TechnicianID.ajll	2.8115394	0.5497669	5.114	3.19e-07 ***
TechnicianID.ajmt	1.1379192	0.4626978	2.459	0.013932 *
TechnicianID.alp	-0.4375347	0.4890933	-0.895	0.371025
TechnicianID.amc	-0.4330602	0.4124896	-1.050	0.293796
TechnicianID.ampp	-0.8519913	0.5706470	-1.493	0.135452
TechnicianID.amr	1.2267461	0.5290890	2.319	0.020431 *
TechnicianID.apv	4.7843407	0.6500635	7.360	1.94e-13 ***
TechnicianID.ara	2.4489622	0.6283504	3.897	9.76e-05 ***
TechnicianID.argf	1.4945882	0.4735459	3.156	0.001602 **
TechnicianID.asp	1.2654065	0.5280788	2.396	0.016577 *
TechnicianID.asv	0.5800429	0.5076786	1.143	0.253249
TechnicianID.atp	-1.3530595	0.4443906	-3.045	0.002333 **
TechnicianID.bag	3.2254153	0.5959342	5.412	6.32e-08 ***
TechnicianID.bgd	0.2291234	0.4581503	0.500	0.617008
TechnicianID.bgm	2.5815574	0.5096158	5.066	4.12e-07 ***
TechnicianID.brm	3.4213290	0.4950650	6.911	5.02e-12 ***
TechnicianID.caa	0.1828530	0.5618984	0.325	0.744868
TechnicianID.cajm	1.2402513	0.4779209	2.595	0.009466 **
TechnicianID.cco	0.9780837	0.5112630	1.913	0.055758 .
TechnicianID.ccp	4.0931857	0.5821685	7.031	2.14e-12 ***
TechnicianID.ccs	-0.3059342	0.4789829	-0.639	0.523018
TechnicianID.cgfj	0.9507131	0.5629035	1.689	0.091251 .
TechnicianID.cja	-0.2546223	0.4118830	-0.618	0.536459
TechnicianID.cjab	0.5304768	0.4637622	1.144	0.252703
TechnicianID.clm	1.4139860	0.7894030	1.791	0.073281 .
TechnicianID.cmc	-0.2369364	0.4538199	-0.522	0.601613
TechnicianID.cmmj	-0.1118715	0.5488225	-0.204	0.838482
TechnicianID.cmr	1.8667570	0.5209437	3.583	0.000340 ***
TechnicianID.cmsg	5.0247395	0.8396476	5.984	2.22e-09 ***
TechnicianID.crlf	0.4486202	0.4872780	0.921	0.357240
TechnicianID.crr	1.7571833	0.4439950	3.958	7.60e-05 ***
TechnicianID.csa	0.6923496	0.7141814	0.969	0.332346
TechnicianID.csp	0.2952449	0.6755486	0.437	0.662085
TechnicianID.dac	1.4568122	0.6638806	2.194	0.028223 *
TechnicianID.dbg	2.2324307	0.5463540	4.086	4.41e-05 ***

TechnicianID.den	-0.5772812	0.4969607	-1.162	0.245408
TechnicianID.dff	2.5466573	0.6331977	4.022	5.80e-05 ***
TechnicianID.dms	1.8945226	0.4952199	3.826	0.000131 ***
TechnicianID.ecc	-0.2408584	0.6072546	-0.397	0.691642
TechnicianID.ecl	2.9349326	0.4500913	6.521	7.23e-11 ***
TechnicianID.efdj	-0.0866760	0.5334851	-0.162	0.870937
TechnicianID.eglc	2.7319355	0.5397818	5.061	4.22e-07 ***
TechnicianID.egn	-0.7280883	0.5659259	-1.287	0.198274
TechnicianID.em	3.0301046	0.5673097	5.341	9.37e-08 ***
TechnicianID.emcc	1.4050355	0.4975640	2.824	0.004752 **
TechnicianID.emm	1.4470331	0.5062615	2.858	0.004266 **
TechnicianID.emp	0.4300562	0.4875900	0.882	0.377789
TechnicianID.epdc	1.1236530	0.4970692	2.261	0.023802 *
TechnicianID.erc	-0.4698136	0.4616886	-1.018	0.308886
TechnicianID.esp	0.2351043	0.6469805	0.363	0.716321
TechnicianID.fbh	0.3159353	0.5580336	0.566	0.571295
TechnicianID.fcv	-1.0505195	0.5016730	-2.094	0.036274 *
TechnicianID.fcv2	-0.9997096	0.4853090	-2.060	0.039422 *
TechnicianID.fdagdlcc	-0.1194793	1.3801759	-0.087	0.931016
TechnicianID.ffm	2.4663629	0.5281785	4.670	3.05e-06 ***
TechnicianID.fjcg	2.2837542	0.4404498	5.185	2.19e-07 ***
TechnicianID.fjdr	4.6423269	0.5209231	8.912	< 2e-16 ***
TechnicianID.fjdr2	1.6282334	0.5265441	3.092	0.001990 **
TechnicianID.fjmh	-1.2935566	0.5675484	-2.279	0.022670 *
TechnicianID.fjml	-1.9828262	0.4901308	-4.046	5.25e-05 ***
TechnicianID.fjmr	0.7309685	0.5447014	1.342	0.179629
TechnicianID.fjnr	-0.6594503	0.5257467	-1.254	0.209749
TechnicianID.fjp	0.3035156	0.4896366	0.620	0.535347
TechnicianID.fjrc	-0.0693954	0.4952797	-0.140	0.888572
TechnicianID.fjrl	1.1485077	0.4838214	2.374	0.017618 *
TechnicianID.fjvm	1.3743749	0.5725770	2.400	0.016393 *
TechnicianID.fll	-1.3013615	0.7898474	-1.648	0.099454 .
TechnicianID.fmf	2.9354831	0.8144447	3.604	0.000314 ***
TechnicianID.fmg	-0.0109798	0.4560787	-0.024	0.980794
TechnicianID.fmg2	1.7837695	0.4427989	4.028	5.64e-05 ***
TechnicianID.fmr	2.5791732	0.6538496	3.945	8.03e-05 ***
TechnicianID.foc	0.4831532	0.5292311	0.913	0.361292
TechnicianID.gdlbml	0.3969727	0.4831022	0.822	0.411252
TechnicianID.grd	0.1867807	0.5023365	0.372	0.710029
TechnicianID.grr	-0.3954438	0.4500133	-0.879	0.379558
TechnicianID.hce	2.9128586	0.4503892	6.467	1.03e-10 ***
TechnicianID.ibr	-0.6041487	0.5456884	-1.107	0.268256
TechnicianID.imjr	-0.1811485	0.5411851	-0.335	0.737837
TechnicianID.irg	-0.3646795	0.5602823	-0.651	0.515131

TechnicianID.izue	0.8642905	0.4564652	1.893	0.058319 .
TechnicianID.jabg	0.7504795	0.4664106	1.609	0.107626
TechnicianID.jacf	0.2378792	0.4457768	0.534	0.593607
TechnicianID.jag	-0.7614807	0.5504852	-1.383	0.166597
TechnicianID.jaga	0.2397488	0.5071056	0.473	0.636378
TechnicianID.jama	3.7932506	0.5457398	6.951	3.79e-12 ***
TechnicianID.japr	-0.4991343	0.4565198	-1.093	0.274260
TechnicianID.jarg	0.6134881	0.6377692	0.962	0.336102
TechnicianID.jbc	1.2419689	0.4728243	2.627	0.008631 **
TechnicianID.jbj	6.8781251	0.9072470	7.581	3.63e-14 ***
TechnicianID.jcac	-1.6823157	0.4671224	-3.601	0.000318 ***
TechnicianID.jccg	-1.3290116	0.5927497	-2.242	0.024969 *
TechnicianID.jcdcp	4.1561492	0.5969020	6.963	3.48e-12 ***
TechnicianID.jdlcrg	0.5372958	0.5110690	1.051	0.293130
TechnicianID.jecc	0.8529024	0.4658357	1.831	0.067135 .
TechnicianID.jerj	1.1790004	0.6017544	1.959	0.050100 .
TechnicianID.jfcm	-0.2272694	0.7297605	-0.311	0.755478
TechnicianID.jfcm2	1.0138654	0.5018964	2.020	0.043395 *
TechnicianID.jgj	0.5624544	0.5348037	1.052	0.292954
TechnicianID.jjcr	-1.2658614	0.5886576	-2.150	0.031538 *
TechnicianID.jjjl	3.3461790	0.5858425	5.712	1.14e-08 ***
TechnicianID.jjpd	-0.7428088	0.4481601	-1.657	0.097448 .
TechnicianID.jjv	1.9938167	0.4652288	4.286	1.83e-05 ***
TechnicianID.jlac	1.2017481	0.5362583	2.241	0.025042 *
TechnicianID.jlg	2.8511866	0.4785929	5.957	2.62e-09 ***
TechnicianID.jlom	1.3525903	0.6238265	2.168	0.030159 *
TechnicianID.jlrr	1.6231707	0.7676839	2.114	0.034500 *
TechnicianID.jmb	2.0111779	0.5320773	3.780	0.000158 ***
TechnicianID.jmbn	2.7265406	0.5354317	5.092	3.58e-07 ***
TechnicianID.jmcb	2.8163605	0.6981067	4.034	5.51e-05 ***
TechnicianID.jmgg	2.9193038	0.4582306	6.371	1.94e-10 ***
TechnicianID.jmgr	-1.1052624	0.6097672	-1.813	0.069915 .
TechnicianID.jmjm	0.8685276	0.4933067	1.761	0.078323 .
TechnicianID.jml	0.1550502	0.6420702	0.241	0.809183
TechnicianID.jmlg	2.8931065	0.5903060	4.901	9.64e-07 ***
TechnicianID.jmm	-1.0990630	0.5791736	-1.898	0.057763 .
TechnicianID.jmrr	3.6139115	0.4731449	7.638	2.34e-14 ***
TechnicianID.jmvg	-0.6721844	0.7140986	-0.941	0.346564
TechnicianID.jmvg2	0.6653958	0.5627325	1.182	0.237052
TechnicianID.jna	-0.7798427	0.5554306	-1.404	0.160331
TechnicianID.jol	2.2994063	0.5080271	4.526	6.05e-06 ***
TechnicianID.jpl	-0.4171258	0.4931784	-0.846	0.397683
TechnicianID.jpl2	1.1569857	0.4678313	2.473	0.013407 *
TechnicianID.jrgp	-0.3197301	0.5923582	-0.540	0.589372

TechnicianID.jrr	0.2417363	0.6427801	0.376	0.706863
TechnicianID.jsl	2.1166177	0.5235272	4.043	5.30e-05 ***
TechnicianID.jsl2	0.0967366	0.4840934	0.200	0.841616
TechnicianID.jvc	1.7323163	0.8659305	2.001	0.045462 *
TechnicianID.jve	-1.1566998	0.6584120	-1.757	0.078973 .
TechnicianID.ladl	0.4887632	0.6847856	0.714	0.475396
TechnicianID.lbe	-0.2964878	0.5145229	-0.576	0.564463
TechnicianID.ljc	2.1384641	0.4850470	4.409	1.05e-05 ***
TechnicianID.llpa	1.8518878	0.7914679	2.340	0.019307 *
TechnicianID.lmgn	-0.2225222	0.4665189	-0.477	0.633380
TechnicianID.lod	0.2506977	0.4556484	0.550	0.582191
TechnicianID.lrg	-0.8322092	0.5514598	-1.509	0.131295
TechnicianID.lrm	-0.4238719	0.5935878	-0.714	0.475186
TechnicianID.madr	-0.6245327	0.6021627	-1.037	0.299684
TechnicianID.magm	1.2909381	0.5161428	2.501	0.012391 *
TechnicianID.mall	0.1275848	0.5055088	0.252	0.800744
TechnicianID.mamg	1.0915852	0.5792947	1.884	0.059540 .
TechnicianID.mars	0.1909817	0.5065725	0.377	0.706174
TechnicianID.mavp	1.1345589	0.6475464	1.752	0.079780 .
TechnicianID.mba	1.7629053	1.2819381	1.375	0.169095
TechnicianID.mbh	0.9618715	0.5681422	1.693	0.090475 .
TechnicianID.mca	2.8738026	0.5258974	5.465	4.72e-08 ***
TechnicianID.mcco	2.9700045	0.6254600	4.749	2.07e-06 ***
TechnicianID.mceypva	3.8170929	0.5785952	6.597	4.34e-11 ***
TechnicianID.mcf	0.3443599	0.5324078	0.647	0.517773
TechnicianID.mcf2	2.4403209	0.5390618	4.527	6.03e-06 ***
TechnicianID.mcg	-0.5485300	0.4834534	-1.135	0.256559
TechnicianID.mcgg	0.0908034	0.5960448	0.152	0.878918
TechnicianID.mcl	-0.0220642	0.4299712	-0.051	0.959075
TechnicianID.mcrc	-0.9758313	0.4473384	-2.181	0.029169 *
TechnicianID.mdcgh	-1.3804154	0.4881581	-2.828	0.004693 **
TechnicianID.mdcls	-0.2240937	0.6442760	-0.348	0.727979
TechnicianID.mdcmc	-0.1622033	0.4469851	-0.363	0.716698
TechnicianID.mdcmr	1.8245197	0.5356557	3.406	0.000661 ***
TechnicianID.mdcto	-0.6684275	0.5254264	-1.272	0.203336
TechnicianID.mdg	0.9008681	0.5442655	1.655	0.097906 .
TechnicianID.mdmmp	0.2891417	0.5617190	0.515	0.606740
TechnicianID.mdrh	2.0517063	0.5219909	3.931	8.51e-05 ***
TechnicianID.mdrmv	0.6737193	0.4371313	1.541	0.123283
TechnicianID.mdrp	3.8763824	0.4932252	7.859	4.14e-15 ***
TechnicianID.mevl	0.1016266	0.4449846	0.228	0.819352
TechnicianID.mfjj	0.8926562	0.5221351	1.710	0.087356 .
TechnicianID.mgcrb	-0.2226881	0.6112817	-0.364	0.715641
TechnicianID.mgjr	0.9267670	0.6233166	1.487	0.137081

TechnicianID.mgm	-0.1467718	0.5672885	-0.259	0.795851
TechnicianID.misd	1.1201271	0.4573591	2.449	0.014332 *
TechnicianID.mjer	0.6306688	0.5971401	1.056	0.290918
TechnicianID.mjjm	0.3028602	0.5192906	0.583	0.559755
TechnicianID.mjmv	0.5915779	0.4542719	1.302	0.192850
TechnicianID.mjtg	0.4313691	0.5022583	0.859	0.390432
TechnicianID.mla	0.6472257	0.4964205	1.304	0.192328
TechnicianID.mlgm	0.3522777	0.4736859	0.744	0.457073
TechnicianID.mlt	-1.2981886	0.5406422	-2.401	0.016354 *
TechnicianID.mma	0.5846973	0.4463112	1.310	0.190194
TechnicianID.mmj	1.2555900	0.4755505	2.640	0.008292 **
TechnicianID.mmm	0.3539818	0.5213648	0.679	0.497179
TechnicianID.mmmm	2.0337289	0.5125721	3.968	7.29e-05 ***
TechnicianID.mmo	0.3607449	0.4501796	0.801	0.422951
TechnicianID.mmp	0.8490539	0.4765778	1.782	0.074841 .
TechnicianID.mms	2.7053050	0.4423927	6.115	9.89e-10 ***
TechnicianID.mpc	-1.8474114	0.7206567	-2.564	0.010372 *
TechnicianID.mpd	1.0679520	0.4346940	2.457	0.014030 *
TechnicianID.mpl	0.5562614	0.4771837	1.166	0.243748
TechnicianID.mrc	-0.5103164	0.4550880	-1.121	0.262154
TechnicianID.mre	-0.8669258	0.6047759	-1.433	0.151746
TechnicianID.mrp	-0.4426751	0.7310542	-0.606	0.544837
TechnicianID.mrr	1.6320228	0.4931675	3.309	0.000938 ***
TechnicianID.mrr2	-1.2187328	0.5061263	-2.408	0.016054 *
TechnicianID.msldc	-0.2536004	0.7873579	-0.322	0.747389
TechnicianID.msm	1.7254836	0.4546701	3.795	0.000148 ***
TechnicianID.mvf	0.5116850	0.5190616	0.986	0.324253
TechnicianID.mvr	3.7389240	0.4812259	7.770	8.40e-15 ***
TechnicianID.nbp	-1.1246142	0.5207231	-2.160	0.030811 *
TechnicianID.nbs	1.9393260	0.4969193	3.903	9.56e-05 ***
TechnicianID.ngs	2.7907209	0.5993858	4.656	3.25e-06 ***
TechnicianID.nrfj	-0.0972062	0.4272656	-0.228	0.820032
TechnicianID.obm	0.6173868	0.4774873	1.293	0.196035
TechnicianID.oyma	-0.8472348	0.5061821	-1.674	0.094196 .
TechnicianID.pagn	-1.3756027	0.5651941	-2.434	0.014951 *
TechnicianID.pbe	-1.3902549	0.4994827	-2.783	0.005386 **
TechnicianID.pcmo	2.0790464	0.4632650	4.488	7.25e-06 ***
TechnicianID.pcv	2.0958656	0.4532948	4.624	3.80e-06 ***
TechnicianID.pdpmda	-0.4159098	0.4199611	-0.990	0.322018
TechnicianID.pjrc	1.6409284	0.6041033	2.716	0.006609 **
TechnicianID.pjst	0.5632423	0.6121930	0.920	0.357567
TechnicianID.pla	-0.5827414	0.7583860	-0.768	0.442264
TechnicianID.plg	3.1914226	0.6171328	5.171	2.35e-07 ***
TechnicianID.plja	0.3093507	0.4730830	0.654	0.513184

TechnicianID.pmc	1.0862887	0.5995561	1.812	0.070034 .
TechnicianID.pmc2	1.1239639	0.4849352	2.318	0.020476 *
TechnicianID.pmm	1.4779980	0.5313770	2.781	0.005419 **
TechnicianID.pmrc	-0.3814513	0.4839977	-0.788	0.430636
TechnicianID.ppf	1.3128787	0.4658153	2.818	0.004832 **
TechnicianID.ppnv	0.1727878	0.6427329	0.269	0.788062
TechnicianID.ppp	-0.2781432	0.5220311	-0.533	0.594174
TechnicianID.prn	1.0607999	0.4817764	2.202	0.027692 *
TechnicianID.psmt	3.8009545	0.4366173	8.705	< 2e-16 ***
TechnicianID.rab	-0.6168091	0.5068159	-1.217	0.223613
TechnicianID.radc	-0.2543674	0.5668244	-0.449	0.653612
TechnicianID.rbl	-0.4325422	0.4623659	-0.935	0.349548
TechnicianID.rcfg	-0.1851933	0.5059373	-0.366	0.714341
TechnicianID.rcg	3.2471881	0.6250082	5.195	2.07e-07 ***
TechnicianID.rcjm	2.6960697	0.5368056	5.022	5.16e-07 ***
TechnicianID.rev	0.7448648	0.5856986	1.272	0.203481
TechnicianID.rga	0.2270958	0.4570702	0.497	0.619302
TechnicianID.rgr	-0.0413595	0.4542100	-0.091	0.927448
TechnicianID.rljm	4.9061360	0.7494414	6.546	6.09e-11 ***
TechnicianID.rlm	0.4031407	0.4792119	0.841	0.400217
TechnicianID.rmm	1.1801686	0.6075524	1.942	0.052096 .
TechnicianID.rmt	-0.8692797	0.4466652	-1.946	0.051655 .
TechnicianID.rop	-0.0876953	0.5699303	-0.154	0.877714
TechnicianID.rro	-0.1655432	0.4539183	-0.365	0.715342
TechnicianID.rrr	0.2982925	0.5109158	0.584	0.559338
TechnicianID.ryf	-0.2057022	0.6042387	-0.340	0.733536
TechnicianID.shm	-1.6332850	0.6611809	-2.470	0.013513 *
TechnicianID.sjs	-1.3726411	0.6299855	-2.179	0.029359 *
TechnicianID.slf	0.2651080	0.4937231	0.537	0.591306
TechnicianID.slr	0.1160130	0.5276563	0.220	0.825980
TechnicianID.sma	0.0727551	0.5150840	0.141	0.887675
TechnicianID.smc	-1.4972887	0.5665479	-2.643	0.008231 **
TechnicianID.spaj	0.4457914	0.5612660	0.794	0.427057
TechnicianID.spl	3.0084021	0.6245209	4.817	1.47e-06 ***
TechnicianID.suj	-0.6559028	0.5697402	-1.151	0.249656
TechnicianID.tig	0.8903205	0.6013430	1.481	0.138747
TechnicianID.tlq	1.9445887	0.4971617	3.911	9.22e-05 ***
TechnicianID.tmf	-0.1188244	0.4656882	-0.255	0.798604
TechnicianID.uk	4.5960182	0.5893023	7.799	6.66e-15 ***
TechnicianID.vrb	-0.0773940	0.5078019	-0.152	0.878866
TechnicianID.vsa	4.6049761	0.4703176	9.791	< 2e-16 ***
Irrigationuk	-0.1204699	0.0526253	-2.289	0.022082 *
Irrigationwater	0.1838455	0.0970854	1.894	0.058292 .
Altitude	0.0026875	0.0001985	13.536	< 2e-16 ***

Signif. codes: 0 ‘***’ 0.001 ‘**’ 0.01 ‘*’ 0.05 ‘.’ 0.1 ‘ ’ 1

Approximate significance of smooth terms:

	edf	Ref.df	F	p-value
s(FieldSize)	4.924	5.609	4.828	9.44e-05 ***
s(Year)	11.952	12.000	304.215	< 2e-16 ***
s(FieldID)	1317.546	4281.000	0.526	< 2e-16 ***
s(xutm,yutm):Year2006	7.144	8.182	5.902	< 2e-16 ***
s(xutm,yutm):Year2007	8.154	8.740	16.851	< 2e-16 ***
s(xutm,yutm):Year2008	8.389	8.832	14.467	< 2e-16 ***
s(xutm,yutm):Year2009	8.513	8.874	18.734	< 2e-16 ***
s(xutm,yutm):Year2010	7.772	8.540	7.620	< 2e-16 ***
s(xutm,yutm):Year2011	7.877	8.597	7.243	< 2e-16 ***
s(xutm,yutm):Year2012	7.195	8.201	5.446	< 2e-16 ***
s(xutm,yutm):Year2013	7.082	8.118	8.430	< 2e-16 ***
s(xutm,yutm):Year2014	7.713	8.537	6.019	< 2e-16 ***
s(xutm,yutm):Year2015	7.286	8.268	10.435	< 2e-16 ***
s(xutm,yutm):Year2016	7.213	8.226	6.130	< 2e-16 ***
s(xutm,yutm):Year2017	8.422	8.861	19.502	< 2e-16 ***
s(xutm,yutm):Year2018	7.801	8.595	8.147	< 2e-16 ***

Signif. codes: 0 ‘***’ 0.001 ‘**’ 0.01 ‘*’ 0.05 ‘.’ 0.1 ‘ ’ 1

R-sq.(adj) =   0.57   Deviance explained = 61.5%
fREML = 34164 Scale est. = 3.4997   n = 16207

2. Effects on insecticides targeting *Bactrocera oleae*

> summary(*BactroceraInsecticides*)

Family: poisson
Link function: log

Formula:
BactroceraInsecticides ~ s(*FieldSize*) + *Cultivar* + *Altitude*+ *Irrigation*+ *OlivesMatrix* + *ShrubsMatrix* + *GrasslandMatrix* + *ForestMatrix* + s(*Year*, bs = "re") + *TechnicianID* + s(*FieldID*, bs = "re") + s(xutm, yutm, bs = "tp", by = *Year*, k = 10)

Parametric coefficients:

	Estimate	Std. Error	z value	Pr(>|z|)

(Intercept)	-4.129e+00	7.497e-01	-5.508	3.63e-08 ***
OlivesMatrix	4.007e-01	9.479e-02	4.228	2.36e-05 ***
ShrubsMatrix	3.671e-01	1.732e-01	2.120	0.034036 *
GrasslandMatrix	7.016e-01	2.491e-01	2.817	0.004851 **
ForestMatrix	1.209e-01	1.889e-01	0.640	0.522197
Cultivar.alo	8.141e-02	6.442e-01	0.126	0.899439
Cultivar.arbe	-3.599e-01	1.418e-01	-2.538	0.011137 *
Cultivar.gordal	-1.145e-01	1.652e-01	-0.693	0.488148
Cultivar.hoji	-7.636e-02	7.980e-02	-0.957	0.338573
Cultivar.lechin	-1.081e-01	7.160e-02	-1.510	0.131171
Cultivar.lucio	-7.589e-01	1.068e+00	-0.710	0.477515
Cultivar.manz	5.908e-02	7.892e-02	0.749	0.454101
Cultivar.morona	-2.452e-01	2.767e-01	-0.886	0.375598
Cultivar.nevadillo	-3.056e-01	2.903e-01	-1.053	0.292352
Cultivar.picolimon	-5.429e-02	1.940e-01	-0.280	0.779593
Cultivar.picual	-7.344e-02	6.656e-02	-1.103	0.269884
Cultivar.picudo	1.766e-01	1.079e-01	1.636	0.101870
Cultivar.verdial	-1.047e-01	1.369e-01	-0.765	0.444160
irrigationuk	-5.883e-04	4.546e-02	-0.013	0.989674
irrigationwater	2.940e-01	1.171e-01	2.510	0.012085 *
Altitude	1.009e-03	1.318e-04	7.653	1.96e-14 ***
TechnicianID.aagm	1.522e+00	7.600e-01	2.003	0.045197 *
TechnicianID.aal	-1.959e+01	2.983e+04	-0.001	0.999476
TechnicianID.acp	-1.891e+01	2.918e+04	-0.001	0.999483
TechnicianID.acs	3.020e-01	1.242e+00	0.243	0.807877
TechnicianID.agam	3.569e+00	7.292e-01	4.895	9.82e-07 ***
TechnicianID.agr	1.211e+00	1.017e+00	1.191	0.233726
TechnicianID.agr2	3.194e+00	7.288e-01	4.383	1.17e-05 ***
TechnicianID.ahm	-1.938e+01	1.068e+04	-0.002	0.998552
TechnicianID.ahs	3.475e+00	7.465e-01	4.655	3.24e-06 ***
TechnicianID.ajbm2	5.485e+00	9.245e-01	5.932	2.99e-09 ***
TechnicianID.ajbr	4.119e+00	8.067e-01	5.106	3.28e-07 ***
TechnicianID.ajll	3.702e+00	7.578e-01	4.885	1.03e-06 ***
TechnicianID.ajmt	3.875e+00	7.514e-01	5.157	2.51e-07 ***
TechnicianID.alp	2.998e+00	7.450e-01	4.024	5.71e-05 ***
TechnicianID.amc	1.689e+00	7.467e-01	2.262	0.023689 *
TechnicianID.ampp	-1.855e+01	1.372e+04	-0.001	0.998921
TechnicianID.amr	2.270e+00	8.329e-01	2.725	0.006420 **
TechnicianID.apv	4.073e+00	7.759e-01	5.250	1.52e-07 ***
TechnicianID.argf	1.972e+00	7.565e-01	2.607	0.009125 **
TechnicianID.asp	1.390e+00	1.024e+00	1.358	0.174402
TechnicianID.asv	-1.771e+01	8.247e+03	-0.002	0.998287
TechnicianID.atp	-1.884e+01	3.421e+03	-0.006	0.995606
TechnicianID.bag	1.351e+00	1.020e+00	1.325	0.185312

TechnicianID.bgd	3.807e+00	7.510e-01	5.070	3.98e-07 ***
TechnicianID.bgm	2.821e+00	7.625e-01	3.700	0.000216 ***
TechnicianID.brm	3.089e+00	7.425e-01	4.161	3.17e-05 ***
TechnicianID.cajm	2.939e+00	1.048e+00	2.804	0.005040 **
TechnicianID.cco	2.299e+00	8.614e-01	2.669	0.007617 **
TechnicianID.ccp	4.515e+00	7.932e-01	5.693	1.25e-08 ***
TechnicianID.ccs	1.815e+00	7.831e-01	2.318	0.020470 *
TechnicianID.cgfj	4.098e+00	7.817e-01	5.243	1.58e-07 ***
TechnicianID.cja	2.637e+00	7.347e-01	3.589	0.000332 ***
TechnicianID.cjab	-1.858e+01	4.795e+03	-0.004	0.996908
TechnicianID.cmc	-1.137e+00	1.241e+00	-0.916	0.359602
TechnicianID.cmr	3.447e+00	7.559e-01	4.560	5.11e-06 ***
TechnicianID.cmsg	3.017e+00	9.040e-01	3.338	0.000845 ***
TechnicianID.crr	2.732e+00	7.416e-01	3.684	0.000230 ***
TechnicianID.csa	4.481e+00	9.383e-01	4.775	1.79e-06 ***
TechnicianID.csp	-1.946e+01	1.841e+04	-0.001	0.999157
TechnicianID.dac	5.298e+00	9.317e-01	5.686	1.30e-08 ***
TechnicianID.dbg	3.308e+00	7.954e-01	4.159	3.19e-05 ***
TechnicianID.den	-1.977e+01	1.003e+04	-0.002	0.998427
TechnicianID.dff	-1.929e+01	2.420e+04	-0.001	0.999364
TechnicianID.dms	4.608e+00	8.192e-01	5.625	1.85e-08 ***
TechnicianID.ecl	9.358e-01	8.320e-01	1.125	0.260667
TechnicianID.efdj	3.296e+00	7.789e-01	4.232	2.32e-05 ***
TechnicianID.eglc	3.557e+00	8.101e-01	4.390	1.13e-05 ***
TechnicianID.egn	-1.854e+01	8.502e+03	-0.002	0.998260
TechnicianID.em	7.951e-01	9.359e-01	0.850	0.395556
TechnicianID.emcc	3.625e+00	7.451e-01	4.865	1.14e-06 ***
TechnicianID.emm	-1.890e+01	5.380e+03	-0.004	0.997197
TechnicianID.emp	7.257e-01	1.242e+00	0.584	0.559101
TechnicianID.epdc	3.058e+00	7.776e-01	3.933	8.38e-05 ***
TechnicianID.erc	4.885e-01	9.315e-01	0.524	0.599977
TechnicianID.esp	-1.826e+01	2.416e+04	-0.001	0.999397
TechnicianID.fcv	2.194e+00	7.579e-01	2.895	0.003797 **
TechnicianID.ffm	1.242e-01	1.018e+00	0.122	0.902889
TechnicianID.fjcg	2.948e+00	7.252e-01	4.065	4.80e-05 ***
TechnicianID.fjdr	3.615e+00	7.700e-01	4.695	2.67e-06 ***
TechnicianID.fjmh	-1.771e+01	4.225e+04	0.000	0.999666
TechnicianID.fjml	-1.917e+01	7.931e+03	-0.002	0.998072
TechnicianID.fjmr	2.169e+00	7.715e-01	2.811	0.004934 **
TechnicianID.fjnr	4.750e-01	1.016e+00	0.467	0.640233
TechnicianID.fjp	3.387e+00	7.478e-01	4.529	5.92e-06 ***
TechnicianID.fjrc	2.831e+00	7.999e-01	3.539	0.000401 ***
TechnicianID.fjrl	2.195e+00	1.245e+00	1.762	0.077986 .
TechnicianID.fjvm	5.182e-01	8.793e-01	0.589	0.555600

TechnicianID.fll	3.244e+00	7.599e-01	4.269	1.97e-05 ***
TechnicianID.fmf	-1.892e+01	4.225e+04	0.000	0.999643
TechnicianID.fmg	8.918e-01	8.865e-01	1.006	0.314445
TechnicianID.fmg2	2.762e+00	7.393e-01	3.737	0.000186 ***
TechnicianID.fmr	3.980e+00	7.671e-01	5.189	2.12e-07 ***
TechnicianID.foc	1.560e+00	8.581e-01	1.818	0.069048 .
TechnicianID.gdlbml	-9.825e-02	1.017e+00	-0.097	0.923022
TechnicianID.grd	1.100e+00	1.017e+00	1.082	0.279304
TechnicianID.grr	-4.297e-01	1.017e+00	-0.423	0.672597
TechnicianID.hce	4.098e+00	7.390e-01	5.546	2.92e-08 ***
TechnicianID.ibr	1.562e+00	8.582e-01	1.820	0.068761 .
TechnicianID.imjr	-1.782e+01	1.302e+04	-0.001	0.998908
TechnicianID.irg	-1.739e+01	9.324e+03	-0.002	0.998512
TechnicianID.izue	3.136e+00	7.246e-01	4.328	1.51e-05 ***
TechnicianID.jabg	-1.883e+01	6.288e+03	-0.003	0.997610
TechnicianID.jacf	-1.901e+01	3.861e+03	-0.005	0.996070
TechnicianID.jag	3.310e+00	7.424e-01	4.459	8.24e-06 ***
TechnicianID.jaga	1.473e+00	9.234e-01	1.595	0.110728
TechnicianID.jama	4.200e+00	7.934e-01	5.294	1.20e-07 ***
TechnicianID.japr	2.103e+00	7.489e-01	2.808	0.004980 **
TechnicianID.jarg	-1.881e+01	8.898e+03	-0.002	0.998314
TechnicianID.jbc	-1.876e+01	4.572e+03	-0.004	0.996727
TechnicianID.jbj	3.198e+00	9.383e-01	3.408	0.000654 ***
TechnicianID.jcac	-1.879e+01	5.198e+03	-0.004	0.997116
TechnicianID.jccg	3.083e+00	7.397e-01	4.168	3.07e-05 ***
TechnicianID.jcdcp	-1.936e+01	1.191e+04	-0.002	0.998702
TechnicianID.jecc	2.799e+00	7.383e-01	3.792	0.000149 ***
TechnicianID.jerj	-1.933e+01	8.400e+03	-0.002	0.998164
TechnicianID.jfcm	-2.074e+01	4.225e+04	0.000	0.999608
TechnicianID.jfcm2	3.672e+00	7.450e-01	4.930	8.24e-07 ***
TechnicianID.jgj	-1.930e+01	8.189e+03	-0.002	0.998120
TechnicianID.jjcr	2.850e+00	7.760e-01	3.672	0.000241 ***
TechnicianID.jjpd	3.985e-01	8.892e-01	0.448	0.654008
TechnicianID.jjv	2.762e+00	7.382e-01	3.742	0.000183 ***
TechnicianID.jlac	1.796e+00	8.229e-01	2.182	0.029073 *
TechnicianID.jlg	3.371e+00	7.227e-01	4.665	3.09e-06 ***
TechnicianID.jlom	1.784e-01	1.228e+00	0.145	0.884512
TechnicianID.jlrr	2.429e+00	8.376e-01	2.900	0.003732 **
TechnicianID.jmb	-1.967e+01	1.579e+04	-0.001	0.999007
TechnicianID.jmbn	3.331e+00	7.246e-01	4.597	4.29e-06 ***
TechnicianID.jmcb	3.106e+00	7.753e-01	4.006	6.17e-05 ***
TechnicianID.jmgg	3.045e+00	7.413e-01	4.108	4.00e-05 ***
TechnicianID.jmgr	-1.868e+01	9.077e+03	-0.002	0.998358
TechnicianID.jmjm	3.623e+00	7.670e-01	4.723	2.33e-06 ***

TechnicianID.jmlg	-1.930e+01	2.111e+04	-0.001	0.999270
TechnicianID.jmm	3.252e+00	7.275e-01	4.470	7.81e-06 ***
TechnicianID.jmrr	4.401e+00	7.508e-01	5.862	4.57e-09 ***
TechnicianID.jmvg2	3.058e+00	7.601e-01	4.023	5.75e-05 ***
TechnicianID.jna	-1.838e+01	1.262e+04	-0.001	0.998838
TechnicianID.jol	2.062e+00	7.661e-01	2.691	0.007121 **
TechnicianID.jpl	-1.889e+01	7.415e+03	-0.003	0.997967
TechnicianID.jpl2	3.260e+00	8.198e-01	3.977	6.99e-05 ***
TechnicianID.jrr	3.420e+00	9.026e-01	3.789	0.000151 ***
TechnicianID.jsl	3.670e+00	7.675e-01	4.781	1.74e-06 ***
TechnicianID.jsl2	-1.803e+01	7.672e+03	-0.002	0.998125
TechnicianID.jve	1.750e+00	8.295e-01	2.110	0.034870 *
TechnicianID.ladl	-1.768e+01	1.500e+04	-0.001	0.999060
TechnicianID.lbe	-1.822e+01	7.848e+03	-0.002	0.998148
TechnicianID.ljc	-1.907e+01	6.064e+03	-0.003	0.997491
TechnicianID.lmgn	1.945e+00	7.734e-01	2.514	0.011924 *
TechnicianID.lod	3.437e+00	7.304e-01	4.705	2.54e-06 ***
TechnicianID.lrg	2.952e+00	7.506e-01	3.933	8.38e-05 ***
TechnicianID.lrm	2.129e+00	8.166e-01	2.607	0.009143 **
TechnicianID.magm	3.136e+00	7.480e-01	4.193	2.76e-05 ***
TechnicianID.mall	-1.865e+01	5.513e+03	-0.003	0.997301
TechnicianID.mamg	3.808e+00	7.493e-01	5.083	3.72e-07 ***
TechnicianID.mars	1.694e+00	8.746e-01	1.937	0.052715 .
TechnicianID.mavp	-1.721e+01	1.328e+04	-0.001	0.998966
TechnicianID.mbh	-1.867e+01	7.654e+03	-0.002	0.998053
TechnicianID.mca	2.628e+00	7.598e-01	3.459	0.000543 ***
TechnicianID.mcco	5.181e+00	8.577e-01	6.041	1.53e-09 ***
TechnicianID.mceypva	4.239e+00	7.732e-01	5.482	4.20e-08 ***
TechnicianID.mcf	7.053e-01	1.244e+00	0.567	0.570635
TechnicianID.mcf2	-1.937e+01	5.918e+03	-0.003	0.997388
TechnicianID.mcg	2.357e+00	7.599e-01	3.102	0.001920 **
TechnicianID.mcgg	-1.752e+01	8.426e+03	-0.002	0.998341
TechnicianID.mcl	-1.111e+00	1.238e+00	-0.898	0.369441
TechnicianID.mcrc	-1.872e+01	3.646e+03	-0.005	0.995903
TechnicianID.mdcgh	2.727e+00	7.490e-01	3.640	0.000272 ***
TechnicianID.mdcls	1.413e+00	1.032e+00	1.369	0.170973
TechnicianID.mdcmc	1.979e+00	7.685e-01	2.575	0.010021 *
TechnicianID.mdcmr	3.053e+00	8.274e-01	3.689	0.000225 ***
TechnicianID.mdcto	3.488e+00	7.515e-01	4.641	3.46e-06 ***
TechnicianID.mdg	2.241e+00	7.913e-01	2.832	0.004631 **
TechnicianID.mdmmp	-1.683e+01	4.225e+04	0.000	0.999682
TechnicianID.mdrh	3.088e+00	7.699e-01	4.012	6.03e-05 ***
TechnicianID.mdrmv	7.456e-01	8.775e-01	0.850	0.395489
TechnicianID.mdrp	2.397e+00	7.568e-01	3.168	0.001536 **

TechnicianID.mevl	1.786e+00	7.737e-01	2.309	0.020943 *
TechnicianID.mfjj	-1.844e+01	9.985e+03	-0.002	0.998527
TechnicianID.mgcrb	-1.859e+01	9.048e+03	-0.002	0.998360
TechnicianID.mgjr	2.900e+00	7.694e-01	3.769	0.000164 ***
TechnicianID.mgm	3.517e+00	7.840e-01	4.486	7.24e-06 ***
TechnicianID.misd	7.715e-01	1.244e+00	0.620	0.535141
TechnicianID.mjer	-1.888e+01	1.014e+04	-0.002	0.998514
TechnicianID.mjjm	3.325e+00	7.902e-01	4.208	2.58e-05 ***
TechnicianID.mjmv	2.890e+00	7.346e-01	3.934	8.34e-05 ***
TechnicianID.mjtg	-1.783e+01	5.940e+03	-0.003	0.997605
TechnicianID.mla	3.092e+00	8.235e-01	3.754	0.000174 ***
TechnicianID.mlgm	2.298e+00	8.008e-01	2.869	0.004113 **
TechnicianID.mlt	2.124e+00	7.715e-01	2.753	0.005903 **
TechnicianID.mma	2.744e+00	7.374e-01	3.721	0.000199 ***
TechnicianID.mmj	3.292e+00	7.359e-01	4.474	7.69e-06 ***
TechnicianID.mmmm	4.430e+00	9.050e-01	4.895	9.83e-07 ***
TechnicianID.mmo	2.857e+00	7.504e-01	3.807	0.000140 ***
TechnicianID.mmp	3.462e+00	7.506e-01	4.613	3.97e-06 ***
TechnicianID.mms	2.903e+00	7.395e-01	3.926	8.62e-05 ***
TechnicianID.mpc	2.728e+00	8.049e-01	3.390	0.000700 ***
TechnicianID.mpd	2.996e+00	7.425e-01	4.035	5.46e-05 ***
TechnicianID.mpl	3.278e+00	7.449e-01	4.400	1.08e-05 ***
TechnicianID.mrc	2.957e+00	7.378e-01	4.008	6.11e-05 ***
TechnicianID.mre	1.980e+00	1.020e+00	1.940	0.052326 .
TechnicianID.mrp	-1.914e+01	1.017e+04	-0.002	0.998498
TechnicianID.mrr	-1.850e+01	9.531e+03	-0.002	0.998451
TechnicianID.mrr2	1.345e+00	1.245e+00	1.081	0.279901
TechnicianID.msldc	2.603e+00	7.985e-01	3.259	0.001118 **
TechnicianID.msm	7.500e-01	1.235e+00	0.608	0.543517
TechnicianID.mvf	8.678e-01	1.022e+00	0.849	0.395615
TechnicianID.nbp	2.587e+00	7.616e-01	3.397	0.000682 ***
TechnicianID.nbs	-1.761e+01	7.977e+03	-0.002	0.998239
TechnicianID.ngs	5.200e+00	8.731e-01	5.956	2.59e-09 ***
TechnicianID.nrfj	8.576e-01	8.120e-01	1.056	0.290894
TechnicianID.obm	3.305e+00	7.695e-01	4.295	1.75e-05 ***
TechnicianID.oyma	1.345e+00	7.811e-01	1.721	0.085166 .
TechnicianID.pagn	8.817e-01	9.494e-01	0.929	0.353021
TechnicianID.pbe	2.443e+00	8.405e-01	2.906	0.003660 **
TechnicianID.pcmo	3.357e+00	7.315e-01	4.588	4.47e-06 ***
TechnicianID.pcv	3.899e+00	7.460e-01	5.227	1.72e-07 ***
TechnicianID.pdpmda	1.828e+00	7.440e-01	2.456	0.014031 *
TechnicianID.pjrc	-1.850e+01	1.506e+04	-0.001	0.999020
TechnicianID.pla	-1.827e+01	1.481e+04	-0.001	0.999015
TechnicianID.plg	1.729e+00	1.020e+00	1.694	0.090209 .

TechnicianID.plja	-1.886e+01	3.756e+03	-0.005	0.995993
TechnicianID.pmc	-1.838e+01	8.387e+03	-0.002	0.998251
TechnicianID.pmc2	3.110e+00	7.358e-01	4.227	2.37e-05 ***
TechnicianID.pmm	2.150e+00	8.918e-01	2.411	0.015892 *
TechnicianID.pmrc	1.901e+00	7.688e-01	2.473	0.013404 *
TechnicianID.ppf	2.302e+00	7.519e-01	3.061	0.002207 **
TechnicianID.ppnv	1.435e+00	1.024e+00	1.402	0.160894
TechnicianID.ppp	-1.884e+01	2.422e+04	-0.001	0.999379
TechnicianID.prn	8.467e-01	8.240e-01	1.028	0.304162
TechnicianID.psmt	3.452e+00	7.256e-01	4.757	1.96e-06 ***
TechnicianID.radc	-1.848e+01	2.371e+04	-0.001	0.999378
TechnicianID.rbl	1.851e+00	7.675e-01	2.411	0.015893 *
TechnicianID.rcfg	-1.802e+01	1.042e+04	-0.002	0.998620
TechnicianID.rcg	3.377e+00	7.854e-01	4.300	1.71e-05 ***
TechnicianID.rev	3.981e+00	7.501e-01	5.307	1.11e-07 ***
TechnicianID.rga	-1.514e+00	1.240e+00	-1.221	0.221907
TechnicianID.rgr	8.327e-01	8.226e-01	1.012	0.311431
TechnicianID.rljm	2.876e+00	8.408e-01	3.421	0.000625 ***
TechnicianID.rlm	-8.208e-01	1.239e+00	-0.663	0.507491
TechnicianID.rmm	3.283e+00	8.084e-01	4.061	4.89e-05 ***
TechnicianID.rmt	7.262e-01	9.164e-01	0.792	0.428078
TechnicianID.rop	4.013e+00	7.557e-01	5.310	1.10e-07 ***
TechnicianID.rro	1.803e+00	7.594e-01	2.374	0.017614 *
TechnicianID.rrr	3.573e+00	7.239e-01	4.937	7.94e-07 ***
TechnicianID.ryf	-1.675e+01	4.225e+04	0.000	0.999684
TechnicianID.shm	2.910e+00	9.347e-01	3.113	0.001851 **
TechnicianID.sjs	7.038e-01	1.042e+00	0.676	0.499255
TechnicianID.slf	-1.810e+01	1.451e+04	-0.001	0.999005
TechnicianID.slr	2.807e+00	7.955e-01	3.528	0.000418 ***
TechnicianID.sma	1.235e+00	9.332e-01	1.323	0.185719
TechnicianID.smc	3.279e+00	7.573e-01	4.330	1.49e-05 ***
TechnicianID.spaj	2.661e+00	7.594e-01	3.504	0.000459 ***
TechnicianID.spl	3.391e+00	7.491e-01	4.526	6.01e-06 ***
TechnicianID.suj	3.292e+00	7.887e-01	4.174	2.99e-05 ***
TechnicianID.tig	3.861e+00	7.424e-01	5.201	1.98e-07 ***
TechnicianID.tlq	-1.744e+01	9.958e+03	-0.002	0.998603
TechnicianID.tmf	2.871e+00	7.441e-01	3.858	0.000114 ***
TechnicianID.vsa	2.256e+00	7.490e-01	3.012	0.002597 **

Signif. codes: 0 '***' 0.001 '**' 0.01 '*' 0.05 '.' 0.1 ' ' 1

Approximate significance of smooth terms:

	edf	Ref.df	Chi.sq	p-value
s(FieldSize)	1.000e+00	1.000	10.97	0.000926 ***

s(Year)	1.174e+01	12.000	353.95	< 2e-16 ***
s(FieldID)	1.232e-04	2668.000	0.00	1.000000
s(xutm,yutm):Year2006	7.293e+00	8.275	41.22	< 2e-16 ***
s(xutm,yutm):Year2007	6.795e+00	7.951	61.47	< 2e-16 ***
s(xutm,yutm):Year2008	8.384e+00	8.850	127.57	< 2e-16 ***
s(xutm,yutm):Year2009	6.340e+00	7.581	44.35	< 2e-16 ***
s(xutm,yutm):Year2010	7.354e+00	8.318	50.45	< 2e-16 ***
s(xutm,yutm):Year2011	8.421e+00	8.880	86.78	< 2e-16 ***
s(xutm,yutm):Year2012	7.912e+00	8.634	88.87	< 2e-16 ***
s(xutm,yutm):Year2013	6.610e+00	7.829	55.85	< 2e-16 ***
s(xutm,yutm):Year2014	6.721e+00	7.797	52.67	< 2e-16 ***
s(xutm,yutm):Year2015	7.343e+00	8.254	113.20	< 2e-16 ***
s(xutm,yutm):Year2016	5.224e+00	6.432	73.27	< 2e-16 ***
s(xutm,yutm):Year2017	3.333e+00	4.183	37.86	< 2e-16 ***
s(xutm,yutm):Year2018	5.699e+00	7.036	38.87	< 2e-16 ***

Signif. codes: 0 ‘***’ 0.001 ‘**’ 0.01 ‘*’ 0.05 ‘.’ 0.1 ‘ ’ 1

R-sq.(adj) = 0.707   Deviance explained = 68.5%
fREML = 10324 Scale est. = 1   n = 9340

3. Effects on *Prays oleae* densities

> summary(*PraysDensity*)

Family: gaussian
Link function: identity

Formula:
PraysDensity ~ s(*FieldSize*) + *Cultivar* + *Altitude*+ *Irrigation*+ *OlivesMatrix* + *ShrubsMatrix* + *GrasslandMatrix* + *ForestMatrix* + s(*Year*, bs = "re") + *TechnicianID* + s(*FieldID*, bs = "re") + s(xutm, yutm, bs = "tp", by = *Year*, k = 10)

Parametric coefficients:

	Estimate	Std. Error	t value	Pr(>|t|)
(Intercept)	71.327399	5.048831	14.128	< 2e-16 ***
OlivesMatrix	2.092937	1.383831	1.512	0.130448
ShrubsMatrix	-10.075917	3.072119	-3.280	0.001041 **
GrasslandMatrix	-12.098180	5.191049	-2.331	0.019789 *
ForestMatrix	0.095656	3.731057	0.026	0.979547
Cultivar.alo	-2.354971	10.442416	-0.226	0.821578
Cultivar.arbe	-1.755574	1.724407	-1.018	0.308659

Cultivar.corni	-6.311761	7.631594	-0.827	0.408218
Cultivar.gordal	-6.773558	2.625650	-2.580	0.009896 **
Cultivar.hoji	-1.826242	1.072377	-1.703	0.088592 .
Cultivar.lechin	-5.195337	1.853456	-2.803	0.005069 **
Cultivar.lucio	-2.944467	6.362306	-0.463	0.643515
Cultivar.manz	-0.462316	1.465154	-0.316	0.752355
Cultivar.morona	0.963879	6.155581	0.157	0.875573
Cultivar.nevadillo	-4.253312	3.768321	-1.129	0.259042
Cultivar.picolimon	3.737803	5.282428	0.708	0.479210
Cultivar.picual	0.530140	0.971505	0.546	0.585288
Cultivar.picudo	-1.631248	2.393631	-0.681	0.495569
Cultivar.verdial	-0.557141	2.972439	-0.187	0.851322
TechnicianID.aagm	-53.757376	4.994094	-10.764	< 2e-16 ***
TechnicianID.aal	-50.383785	5.532985	-9.106	< 2e-16 ***
TechnicianID.abta	-51.589489	7.387480	-6.983	3.00e-12 ***
TechnicianID.acl	-61.814926	6.207337	-9.958	< 2e-16 ***
TechnicianID.acp	-52.241143	5.770385	-9.053	< 2e-16 ***
TechnicianID.acs	-44.579368	5.345477	-8.340	< 2e-16 ***
TechnicianID.agam	-56.037245	4.966309	-11.283	< 2e-16 ***
TechnicianID.agl	-61.724427	24.211387	-2.549	0.010801 *
TechnicianID.agr	-56.092409	4.856884	-11.549	< 2e-16 ***
TechnicianID.agr2	-37.401651	4.789245	-7.810	6.13e-15 ***
TechnicianID.ahm	-30.942854	5.034376	-6.146	8.13e-10 ***
TechnicianID.ahs	-53.148114	5.105808	-10.409	< 2e-16 ***
TechnicianID.ajbm2	-60.106559	12.915175	-4.654	3.28e-06 ***
TechnicianID.ajbr	-62.318210	6.159078	-10.118	< 2e-16 ***
TechnicianID.ajll	-54.697176	6.213029	-8.804	< 2e-16 ***
TechnicianID.ajmt	-36.603535	5.289367	-6.920	4.70e-12 ***
TechnicianID.alp	-52.029692	5.221945	-9.964	< 2e-16 ***
TechnicianID.amc	-48.986101	4.455501	-10.995	< 2e-16 ***
TechnicianID.ampp	-58.298045	6.393279	-9.119	< 2e-16 ***
TechnicianID.amr	-18.695199	5.933748	-3.151	0.001632 **
TechnicianID.apv	-56.702945	7.209302	-7.865	3.94e-15 ***
TechnicianID.ara	-56.060317	6.950263	-8.066	7.82e-16 ***
TechnicianID.argf	-21.367637	5.006534	-4.268	1.99e-05 ***
TechnicianID.asp	-23.611526	5.598132	-4.218	2.48e-05 ***
TechnicianID.asv	-40.544006	5.664164	-7.158	8.57e-13 ***
TechnicianID.atp	-35.964757	4.791060	-7.507	6.41e-14 ***
TechnicianID.bag	36.073835	6.495767	5.553	2.85e-08 ***
TechnicianID.bgd	-50.565314	5.087820	-9.939	< 2e-16 ***
TechnicianID.bgm	-52.395504	5.571175	-9.405	< 2e-16 ***
TechnicianID.brm	-45.369786	5.527983	-8.207	2.45e-16 ***
TechnicianID.caa	-50.195010	6.003514	-8.361	< 2e-16 ***
TechnicianID.cajm	-55.141261	5.125945	-10.757	< 2e-16 ***

TechnicianID.cco	-42.730062	5.485659	-7.789	7.18e-15 ***
TechnicianID.ccp	-53.096034	6.251749	-8.493	< 2e-16 ***
TechnicianID.ccs	-59.910395	5.128109	-11.683	< 2e-16 ***
TechnicianID.cgfj	-29.856652	6.707642	-4.451	8.60e-06 ***
TechnicianID.cja	-58.297309	4.457116	-13.080	< 2e-16 ***
TechnicianID.cjab	-47.878195	4.979362	-9.615	< 2e-16 ***
TechnicianID.clm	-96.420228	24.321934	-3.964	7.39e-05 ***
TechnicianID.cmc	-44.326735	4.922894	-9.004	< 2e-16 ***
TechnicianID.cmmj	-42.809577	6.830638	-6.267	3.78e-10 ***
TechnicianID.cmr	-54.987786	5.877073	-9.356	< 2e-16 ***
TechnicianID.cmsg	-60.176062	8.563448	-7.027	2.20e-12 ***
TechnicianID.crlf	-55.466523	5.233038	-10.599	< 2e-16 ***
TechnicianID.crr	-57.256989	4.857820	-11.787	< 2e-16 ***
TechnicianID.csa	-61.260044	8.068766	-7.592	3.33e-14 ***
TechnicianID.csp	-57.472724	8.360909	-6.874	6.49e-12 ***
TechnicianID.dac	-52.583425	12.467467	-4.218	2.48e-05 ***
TechnicianID.dbg	-60.542816	6.066695	-9.980	< 2e-16 ***
TechnicianID.den	-55.800936	5.308021	-10.513	< 2e-16 ***
TechnicianID.dff	73.425706	6.899334	10.642	< 2e-16 ***
TechnicianID.dms	-59.032914	5.584435	-10.571	< 2e-16 ***
TechnicianID.ecc	-49.959351	6.801233	-7.346	2.15e-13 ***
TechnicianID.ecl	-40.960778	4.908847	-8.344	< 2e-16 ***
TechnicianID.efdj	-50.779683	5.927157	-8.567	< 2e-16 ***
TechnicianID.eglc	-56.188957	5.777443	-9.726	< 2e-16 ***
TechnicianID.egn	-34.386965	6.063240	-5.671	1.44e-08 ***
TechnicianID.em	-30.520943	6.268237	-4.869	1.13e-06 ***
TechnicianID.emcc	-56.356944	5.395608	-10.445	< 2e-16 ***
TechnicianID.emm	-28.380628	5.490010	-5.170	2.38e-07 ***
TechnicianID.emp	-60.655317	5.273621	-11.502	< 2e-16 ***
TechnicianID.epdc	-56.460624	5.376647	-10.501	< 2e-16 ***
TechnicianID.erc	-10.062514	5.046805	-1.994	0.046188 *
TechnicianID.esp	-58.423717	7.209553	-8.104	5.75e-16 ***
TechnicianID.fbh	-42.569131	6.019917	-7.071	1.60e-12 ***
TechnicianID.fcv	-49.245219	5.592184	-8.806	< 2e-16 ***
TechnicianID.fcv2	-65.419922	5.512546	-11.867	< 2e-16 ***
TechnicianID.fdagdlcc	-58.893137	15.317106	-3.845	0.000121 ***
TechnicianID.ffm	19.931718	5.881831	3.389	0.000704 ***
TechnicianID.fjcg	-26.045001	4.743543	-5.491	4.07e-08 ***
TechnicianID.fjdr	13.115063	6.773588	1.936	0.052862 .
TechnicianID.fjdr2	-41.058978	5.841681	-7.029	2.18e-12 ***
TechnicianID.fjmh	-40.543928	6.231974	-6.506	7.98e-11 ***
TechnicianID.fjml	-47.846231	5.222192	-9.162	< 2e-16 ***
TechnicianID.fjmr	-63.070649	5.874020	-10.737	< 2e-16 ***
TechnicianID.fjnr	-60.902694	5.594881	-10.885	< 2e-16 ***

TechnicianID.fjp	-59.765353	5.298434	-11.280	< 2e-16 ***
TechnicianID.fjrc	-49.817100	5.314884	-9.373	< 2e-16 ***
TechnicianID.fjrl	-56.690476	5.318745	-10.659	< 2e-16 ***
TechnicianID.fjvm	17.981566	6.283166	2.862	0.004217 **
TechnicianID.fll	-46.149974	9.387234	-4.916	8.92e-07 ***
TechnicianID.fmf	-55.134014	9.043021	-6.097	1.11e-09 ***
TechnicianID.fmg	-41.477577	5.006836	-8.284	< 2e-16 ***
TechnicianID.fmg2	-53.720813	4.818830	-11.148	< 2e-16 ***
TechnicianID.fmr	-8.613030	7.142127	-1.206	0.227857
TechnicianID.foc	-56.094769	5.814575	-9.647	< 2e-16 ***
TechnicianID.gdlbml	-21.976901	5.108265	-4.302	1.70e-05 ***
TechnicianID.grd	-44.680157	5.427662	-8.232	< 2e-16 ***
TechnicianID.grr	-7.382716	4.921018	-1.500	0.133573
TechnicianID.hce	-54.381396	4.956440	-10.972	< 2e-16 ***
TechnicianID.ibr	-47.210916	5.957401	-7.925	2.45e-15 ***
TechnicianID.imjr	-50.495366	5.952764	-8.483	< 2e-16 ***
TechnicianID.irg	-59.398281	6.242972	-9.514	< 2e-16 ***
TechnicianID.izue	-55.570303	4.918906	-11.297	< 2e-16 ***
TechnicianID.jabg	-33.138782	5.079944	-6.523	7.09e-11 ***
TechnicianID.jacf	-51.053715	4.984044	-10.243	< 2e-16 ***
TechnicianID.jag	-54.262301	8.535532	-6.357	2.11e-10 ***
TechnicianID.jaga	-61.255324	5.415307	-11.312	< 2e-16 ***
TechnicianID.jama	-65.607656	6.179119	-10.618	< 2e-16 ***
TechnicianID.japr	-51.411459	4.947807	-10.391	< 2e-16 ***
TechnicianID.jarg	40.504883	6.782196	5.972	2.39e-09 ***
TechnicianID.jbc	-37.409690	5.040035	-7.423	1.21e-13 ***
TechnicianID.jbj	-41.301348	10.754150	-3.841	0.000123 ***
TechnicianID.jcac	-55.354421	4.931106	-11.226	< 2e-16 ***
TechnicianID.jccg	-60.844551	6.529875	-9.318	< 2e-16 ***
TechnicianID.jcdcp	52.052575	6.319942	8.236	< 2e-16 ***
TechnicianID.jdlcrg	-57.948045	5.697058	-10.172	< 2e-16 ***
TechnicianID.jecc	-55.329757	4.950096	-11.178	< 2e-16 ***
TechnicianID.jerj	18.906355	6.829285	2.768	0.005640 **
TechnicianID.jfcm	-62.807457	8.168150	-7.689	1.57e-14 ***
TechnicianID.jfcm2	-56.189635	5.435909	-10.337	< 2e-16 ***
TechnicianID.jgj	-63.600442	5.817067	-10.933	< 2e-16 ***
TechnicianID.jjcr	-54.196104	6.537310	-8.290	< 2e-16 ***
TechnicianID.jjjl	-50.616242	6.327982	-7.999	1.35e-15 ***
TechnicianID.jjpd	-54.350926	4.866312	-11.169	< 2e-16 ***
TechnicianID.jjv	-54.140329	4.983719	-10.863	< 2e-16 ***
TechnicianID.jlac	-12.234798	5.795396	-2.111	0.034778 *
TechnicianID.jlg	-22.255701	5.018368	-4.435	9.28e-06 ***
TechnicianID.jlom	83.210148	6.984923	11.913	< 2e-16 ***
TechnicianID.jlrr	-49.604061	8.266761	-6.000	2.01e-09 ***

TechnicianID.jmb	-57.233080	5.874941	-9.742	< 2e-16 ***
TechnicianID.jmbn	-25.443841	5.962474	-4.267	1.99e-05 ***
TechnicianID.jmcb	-58.288138	7.269683	-8.018	1.16e-15 ***
TechnicianID.jmgg	18.108261	5.012228	3.613	0.000304 ***
TechnicianID.jmgr	-11.624481	6.863448	-1.694	0.090347 .
TechnicianID.jmjm	-57.995261	5.406484	-10.727	< 2e-16 ***
TechnicianID.jml	-63.842091	7.344610	-8.692	< 2e-16 ***
TechnicianID.jmlg	-76.262443	6.592990	-11.567	< 2e-16 ***
TechnicianID.jmm	-52.546846	6.345857	-8.280	< 2e-16 ***
TechnicianID.jmrr	-52.590551	5.136849	-10.238	< 2e-16 ***
TechnicianID.jmvg	-61.532812	7.875525	-7.813	5.95e-15 ***
TechnicianID.jmvg2	-55.690646	6.068790	-9.177	< 2e-16 ***
TechnicianID.jna	-42.267109	6.029016	-7.011	2.48e-12 ***
TechnicianID.jol	-51.417741	5.498155	-9.352	< 2e-16 ***
TechnicianID.jpl	-36.142125	5.358654	-6.745	1.59e-11 ***
TechnicianID.jpl2	-56.792752	5.226765	-10.866	< 2e-16 ***
TechnicianID.jrgp	-43.292178	6.705957	-6.456	1.11e-10 ***
TechnicianID.jrr	-44.442555	6.993844	-6.355	2.15e-10 ***
TechnicianID.jsl	-49.821920	5.667046	-8.792	< 2e-16 ***
TechnicianID.jsl2	-56.284329	5.294160	-10.631	< 2e-16 ***
TechnicianID.jvc	-53.739847	10.827928	-4.963	7.02e-07 ***
TechnicianID.jve	-58.758423	7.133061	-8.237	< 2e-16 ***
TechnicianID.ladl	-57.277035	7.777278	-7.365	1.87e-13 ***
TechnicianID.lbe	-58.770699	5.374513	-10.935	< 2e-16 ***
TechnicianID.ljc	-45.660939	5.198960	-8.783	< 2e-16 ***
TechnicianID.llpa	-55.906944	9.147573	-6.112	1.01e-09 ***
TechnicianID.lmgn	-54.053074	5.075894	-10.649	< 2e-16 ***
TechnicianID.lod	-55.000368	4.955068	-11.100	< 2e-16 ***
TechnicianID.lrg	-66.708727	5.996241	-11.125	< 2e-16 ***
TechnicianID.lrm	-55.577765	6.486697	-8.568	< 2e-16 ***
TechnicianID.madr	-27.124886	6.315870	-4.295	1.76e-05 ***
TechnicianID.magm	-59.598027	5.824503	-10.232	< 2e-16 ***
TechnicianID.mall	27.911233	5.442026	5.129	2.95e-07 ***
TechnicianID.mamg	-62.713248	6.478106	-9.681	< 2e-16 ***
TechnicianID.mars	-51.004036	5.665710	-9.002	< 2e-16 ***
TechnicianID.mavp	-52.626918	7.520619	-6.998	2.71e-12 ***
TechnicianID.mbh	58.118831	6.082743	9.555	< 2e-16 ***
TechnicianID.mca	-41.955727	5.691831	-7.371	1.78e-13 ***
TechnicianID.mcco	-59.306461	12.402892	-4.782	1.76e-06 ***
TechnicianID.mceypva	-62.019418	6.414606	-9.668	< 2e-16 ***
TechnicianID.mcf	-51.235092	5.663174	-9.047	< 2e-16 ***
TechnicianID.mcf2	46.858461	5.778860	8.109	5.52e-16 ***
TechnicianID.mcg	-56.682846	5.260991	-10.774	< 2e-16 ***
TechnicianID.mcgg	-48.176405	6.609080	-7.289	3.27e-13 ***

TechnicianID.mcl	-12.771005	4.643785	-2.750	0.005964 **
TechnicianID.mcrc	-31.774082	4.816300	-6.597	4.33e-11 ***
TechnicianID.mdcgh	-52.637213	5.413987	-9.722	< 2e-16 ***
TechnicianID.mdcls	-56.437368	7.154212	-7.889	3.27e-15 ***
TechnicianID.mdcmc	-53.137138	5.072234	-10.476	< 2e-16 ***
TechnicianID.mdcmr	-60.398237	5.905296	-10.228	< 2e-16 ***
TechnicianID.mdcto	-61.622281	5.815633	-10.596	< 2e-16 ***
TechnicianID.mdg	-54.177125	5.945030	-9.113	< 2e-16 ***
TechnicianID.mdmmp	-63.827450	6.242717	-10.224	< 2e-16 ***
TechnicianID.mdrh	-53.294723	5.966912	-8.932	< 2e-16 ***
TechnicianID.mdrmv	-48.305771	4.850439	9.959	< 2e-16 ***
TechnicianID.mdrp	-25.205681	5.309770	-4.747	2.08e-06 ***
TechnicianID.mevl	-48.094154	4.844959	-9.927	< 2e-16 ***
TechnicianID.mfjj	9.824187	5.876725	1.672	0.094603 .
TechnicianID.mgcrb	-52.699533	6.743656	-7.815	5.88e-15 ***
TechnicianID.mgjr	-55.974407	6.736479	-8.309	< 2e-16 ***
TechnicianID.mgm	-63.178788	6.380107	-9.902	< 2e-16 ***
TechnicianID.misd	-60.935549	5.067371	-12.025	< 2e-16 ***
TechnicianID.mjer	-46.856902	6.900047	-6.791	1.16e-11 ***
TechnicianID.mjjm	-53.087807	5.786672	-9.174	< 2e-16 ***
TechnicianID.mjmv	-31.148387	4.939128	-6.306	2.94e-10 ***
TechnicianID.mjtg	-53.990753	5.590733	-9.657	< 2e-16 ***
TechnicianID.mla	-51.819790	5.306787	-9.765	< 2e-16 ***
TechnicianID.mlgm	-53.566185	5.141719	-10.418	< 2e-16 ***
TechnicianID.mlt	-53.626285	6.008324	-8.925	< 2e-16 ***
TechnicianID.mma	-48.989095	5.088010	-9.628	< 2e-16 ***
TechnicianID.mmj	-54.939451	5.307647	-10.351	< 2e-16 ***
TechnicianID.mmm	-39.815782	5.678353	-7.012	2.45e-12 ***
TechnicianID.mmmm	-59.708963	5.655110	-10.558	< 2e-16 ***
TechnicianID.mmo	-44.618914	4.975747	-8.967	< 2e-16 ***
TechnicianID.mmp	-52.135907	5.125308	-10.172	< 2e-16 ***
TechnicianID.mms	26.914947	4.816385	5.588	2.33e-08 ***
TechnicianID.mpc	-55.788126	7.625701	-7.316	2.69e-13 ***
TechnicianID.mpd	-51.971472	4.740373	-10.964	< 2e-16 ***
TechnicianID.mpl	-41.025243	5.209332	-7.875	3.63e-15 ***
TechnicianID.mrc	-66.474599	4.931081	-13.481	< 2e-16 ***
TechnicianID.mre	-56.476360	6.702452	-8.426	< 2e-16 ***
TechnicianID.mrp	-53.938438	8.339389	-6.468	1.02e-10 ***
TechnicianID.mrr	-38.090861	5.315163	-7.166	8.06e-13 ***
TechnicianID.mrr2	-54.503452	5.420710	-10.055	< 2e-16 ***
TechnicianID.msldc	-55.419277	7.188363	-7.710	1.34e-14 ***
TechnicianID.msm	-57.054266	5.033203	-11.336	< 2e-16 ***
TechnicianID.mvf	-46.531743	5.619369	-8.281	< 2e-16 ***
TechnicianID.mvr	-38.174612	5.975522	-6.388	1.73e-10 ***

TechnicianID.nbp	-51.216012	5.770555	-8.875	< 2e-16 ***
TechnicianID.nbs	-61.552936	5.278632	-11.661	< 2e-16 ***
TechnicianID.ngs	-59.029505	8.083478	-7.302	2.97e-13 ***
TechnicianID.nrfj	-48.330447	4.630186	-10.438	< 2e-16 ***
TechnicianID.obm	-50.357225	5.175259	-9.730	< 2e-16 ***
TechnicianID.oyma	-58.414791	5.474383	-10.671	< 2e-16 ***
TechnicianID.pagn	-54.907820	6.149568	-8.929	< 2e-16 ***
TechnicianID.pbe	-61.933191	5.381075	-11.509	< 2e-16 ***
TechnicianID.pcmo	-52.736835	5.007967	-10.531	< 2e-16 ***
TechnicianID.pcv	-58.640354	4.966453	-11.807	< 2e-16 ***
TechnicianID.pdpmda	-41.089502	4.663935	-8.810	< 2e-16 ***
TechnicianID.pjrc	-59.565596	6.321679	-9.422	< 2e-16 ***
TechnicianID.pjst	-62.808851	6.835886	-9.188	< 2e-16 ***
TechnicianID.pla	-60.242166	8.515586	-7.074	1.57e-12 ***
TechnicianID.plg	21.697036	6.860362	3.163	0.001566 **
TechnicianID.plja	-52.113547	5.038182	-10.344	< 2e-16 ***
TechnicianID.pmc	-23.340845	6.764711	-3.450	0.000561 ***
TechnicianID.pmc2	-48.113422	5.146430	-9.349	< 2e-16 ***
TechnicianID.pmm	-50.045160	5.748516	-8.706	< 2e-16 ***
TechnicianID.pmrc	-61.516162	5.259634	-11.696	< 2e-16 ***
TechnicianID.ppf	-53.827839	5.002831	-10.759	< 2e-16 ***
TechnicianID.ppnv	-33.348607	6.678801	-4.993	6.01e-07 ***
TechnicianID.ppp	-56.166915	5.653495	-9.935	< 2e-16 ***
TechnicianID.prn	33.527060	5.191671	6.458	1.10e-10 ***
TechnicianID.psmt	-42.408783	4.730297	-8.965	< 2e-16 ***
TechnicianID.rab	-55.676531	5.619670	-9.907	< 2e-16 ***
TechnicianID.radc	-56.620130	6.136590	-9.227	< 2e-16 ***
TechnicianID.rbl	-69.488208	4.988146	-13.931	< 2e-16 ***
TechnicianID.rcfg	-47.720434	5.506287	-8.667	< 2e-16 ***
TechnicianID.rcg	-64.327908	6.878362	-9.352	< 2e-16 ***
TechnicianID.rcjm	-54.877524	5.696662	-9.633	< 2e-16 ***
TechnicianID.rev	-24.114076	7.576110	-3.183	0.001461 **
TechnicianID.rga	-51.664702	4.875678	-10.596	< 2e-16 ***
TechnicianID.rgr	-6.512096	4.963078	-1.312	0.189504
TechnicianID.rljm	-54.465362	8.485986	-6.418	1.42e-10 ***
TechnicianID.rlm	-15.811548	5.140357	-3.076	0.002102 **
TechnicianID.rmm	-46.970927	6.807475	-6.900	5.42e-12 ***
TechnicianID.rmt	-39.612812	4.955458	-7.994	1.41e-15 ***
TechnicianID.rop	-24.606641	7.712482	-3.190	0.001423 **
TechnicianID.rro	-57.809956	4.910796	-11.772	< 2e-16 ***
TechnicianID.rrr	-61.472688	5.718906	-10.749	< 2e-16 ***
TechnicianID.ryf	-65.910488	6.673627	-9.876	< 2e-16 ***
TechnicianID.shm	-53.089870	7.421099	-7.154	8.83e-13 ***
TechnicianID.sjs	-58.630310	6.945289	-8.442	< 2e-16 ***

TechnicianID.slf	-58.253993	5.451125	-10.687	< 2e-16 ***
TechnicianID.slr	-63.977799	5.783956	-11.061	< 2e-16 ***
TechnicianID.sma	-41.975375	5.654145	-7.424	1.20e-13 ***
TechnicianID.smc	-25.252358	6.197246	-4.075	4.63e-05 ***
TechnicianID.spaj	-58.909062	6.226544	-9.461	< 2e-16 ***
TechnicianID.spl	-40.801382	6.538273	-6.240	4.48e-10 ***
TechnicianID.suj	-59.845849	6.329792	-9.455	< 2e-16 ***
TechnicianID.tig	-8.448056	6.718505	-1.257	0.208618
TechnicianID.tlq	-59.753971	5.316780	-11.239	< 2e-16 ***
TechnicianID.tmf	-66.884944	5.047862	-13.250	< 2e-16 ***
TechnicianID.uk	-55.523398	7.545785	-7.358	1.96e-13 ***
TechnicianID.vrb	-63.587837	5.638810	-11.277	< 2e-16 ***
TechnicianID.vsa	-22.561029	5.049418	-4.468	7.95e-06 ***
irrigationuk	1.805924	0.582199	3.102	0.001926 **
irrigationwater	2.260518	1.101348	2.053	0.040139 *
Altitude	-0.003501	0.002205	-1.588	0.112393

Signif. codes: 0 ‘***’ 0.001 ‘**’ 0.01 ‘*’ 0.05 ‘.’ 0.1 ‘ ’ 1

Approximate significance of smooth terms:

	edf	Ref.df	F	p-value
s(FieldSize)	4.107	4.864	1.990	0.0726 .
s(Year)	11.929	12.000	136.180	< 2e-16 ***
s(FieldID)	770.542	4264.000	0.239	< 2e-16 ***
s(xutm,yutm):Year2006	8.887	8.986	66.778	< 2e-16 ***
s(xutm,yutm):Year2007	8.045	8.735	6.492	< 2e-16 ***
s(xutm,yutm):Year2008	7.761	8.589	5.233	< 2e-16 ***
s(xutm,yutm):Year2009	8.687	8.945	18.769	< 2e-16 ***
s(xutm,yutm):Year2010	8.092	8.738	8.366	< 2e-16 ***
s(xutm,yutm):Year2011	7.659	8.531	4.694	2.71e-05 ***
s(xutm,yutm):Year2012	5.963	7.260	1.583	0.1126
s(xutm,yutm):Year2013	7.910	8.675	13.285	< 2e-16 ***
s(xutm,yutm):Year2014	8.444	8.887	21.291	< 2e-16 ***
s(xutm,yutm):Year2015	7.156	8.239	4.764	5.74e-06 ***
s(xutm,yutm):Year2016	5.293	6.611	2.357	0.0231 *
s(xutm,yutm):Year2017	7.478	8.468	4.988	4.96e-07 ***
s(xutm,yutm):Year2018	8.181	8.804	13.008	< 2e-16 ***

Signif. codes: 0 ‘***’ 0.001 ‘**’ 0.01 ‘*’ 0.05 ‘.’ 0.1 ‘ ’ 1

R-sq.(adj) = 0.595   Deviance explained = 62.5%
fREML = 72341 Scale est. = 522.8   n = 15944

4. Effects on insecticides targeting *Prays oleae*

> summary(*PraysInsecticides*)

Family: poisson
Link function: log

Formula:
PraysInsecticides ~ s(*FieldSize*) + *Cultivar* + *Altitude* + *Irrigation* + *OlivesMatrix* + *ShrubsMatrix* + *GrasslandMatrix* + *ForestMatrix* + s(*Year*, bs = "re") + *TechnicianID* + s(*FieldID*, bs = "re") + s(xutm, yutm, bs = "tp", by = *Year*, k = 10)

Parametric coefficients:

	Estimate	Std. Error	z value	Pr(>|z|)
(Intercept)	-1.741e+00	3.548e-01	-4.906	9.28e-07 ***
OlivesMatrix	-2.866e-02	9.249e-02	-0.310	0.756676
ShrubsMatrix	-1.077e-01	2.257e-01	-0.477	0.633196
GrasslandMatrix	3.280e-01	4.130e-01	0.794	0.427127
ForestMatrix	2.606e-01	3.361e-01	0.775	0.438174
Cultivar.alo	-1.791e+01	3.256e+03	-0.006	0.995610
Cultivar.arbe	-7.495e-02	1.198e-01	-0.626	0.531440
Cultivar.gordal	7.930e-02	1.515e-01	0.524	0.600620
Cultivar.hoji	-2.285e-03	8.502e-02	-0.027	0.978557
Cultivar.lechin	-8.386e-02	1.464e-01	-0.573	0.566714
Cultivar.lucio	-9.434e-01	7.445e-01	-1.267	0.205059
Cultivar.manz	8.517e-02	9.881e-02	0.862	0.388692
Cultivar.morona	4.296e-01	2.684e-01	1.601	0.109474
Cultivar.nevadillo	-9.888e-01	5.688e-01	-1.739	0.082100 .
Cultivar.picolimon	-1.567e+01	9.319e+02	-0.017	0.986580
Cultivar.picual	4.100e-02	8.815e-02	0.465	0.641865
Cultivar.picudo	-2.784e-01	2.293e-01	-1.214	0.224757
Cultivar.verdial	1.637e-01	1.830e-01	0.895	0.370977
irrigationuk	6.899e-02	3.741e-02	1.844	0.065177 .
irrigationwater	1.776e-01	7.931e-02	2.240	0.025122 *
Altitude	-3.130e-04	2.027e-04	-1.544	0.122619
TechnicianID.aagm	1.246e+00	3.674e-01	3.391	0.000695 ***
TechnicianID.aal	-1.676e+01	4.042e+03	-0.004	0.996692
TechnicianID.acp	-1.655e+01	3.997e+03	-0.004	0.996696
TechnicianID.acs	1.384e+00	4.135e-01	3.347	0.000818 ***
TechnicianID.agam	-1.613e+01	5.702e+02	-0.028	0.977427
TechnicianID.agr	-6.098e-01	7.874e-01	-0.774	0.438659
TechnicianID.agr2	2.403e-01	4.159e-01	0.578	0.563461

TechnicianID.ahm	1.670e+00	4.291e-01	3.891	9.98e-05 ***
TechnicianID.ahs	3.132e-01	4.008e-01	0.781	0.434509
TechnicianID.ajbm2	-1.473e+01	1.439e+03	-0.010	0.991833
TechnicianID.ajbr	-8.591e-02	6.333e-01	-0.136	0.892094
TechnicianID.ajll	-1.586e+01	1.151e+03	-0.014	0.989002
TechnicianID.ajmt	-2.028e+00	1.066e+00	-1.902	0.057158 .
TechnicianID.alp	4.507e-01	4.211e-01	1.070	0.284511
TechnicianID.amc	9.885e-01	3.568e-01	2.771	0.005596 **
TechnicianID.ampp	-1.689e+01	1.900e+03	-0.009	0.992910
TechnicianID.amr	8.358e-01	5.340e-01	1.565	0.117562
TechnicianID.apv	-1.678e+01	1.162e+03	-0.014	0.988473
TechnicianID.argf	1.470e+00	3.541e-01	4.152	3.30e-05 ***
TechnicianID.asp	2.285e+00	3.879e-01	5.892	3.82e-09 ***
TechnicianID.asv	-6.359e-02	7.909e-01	-0.080	0.935916
TechnicianID.atp	1.758e+00	3.528e-01	4.981	6.32e-07 ***
TechnicianID.bag	1.754e+00	4.312e-01	4.068	4.75e-05 ***
TechnicianID.bgd	-1.862e+00	1.062e+00	-1.753	0.079620 .
TechnicianID.bgm	-1.611e+01	8.869e+02	-0.018	0.985504
TechnicianID.brm	-1.614e+01	8.376e+02	-0.019	0.984630
TechnicianID.cajm	-1.716e+01	5.718e+03	-0.003	0.997605
TechnicianID.cco	2.211e+00	3.871e-01	5.712	1.12e-08 ***
TechnicianID.ccp	-1.708e+01	1.508e+03	-0.011	0.990961
TechnicianID.ccs	8.537e-01	4.571e-01	1.868	0.061804 .
TechnicianID.cgfj	-1.694e+01	1.353e+03	-0.013	0.990005
TechnicianID.cja	8.641e-01	3.630e-01	2.381	0.017282 *
TechnicianID.cjab	1.388e+00	3.674e-01	3.777	0.000159 ***
TechnicianID.cmc	1.557e+00	3.811e-01	4.084	4.43e-05 ***
TechnicianID.cmr	-2.242e+00	1.070e+00	-2.096	0.036108 *
TechnicianID.cmsg	4.163e-01	5.923e-01	0.703	0.482125
TechnicianID.crr	1.983e-01	4.284e-01	0.463	0.643545
TechnicianID.csa	-1.468e+01	1.983e+03	-0.007	0.994093
TechnicianID.csp	8.671e-01	7.988e-01	1.085	0.277721
TechnicianID.dac	-1.528e+01	2.141e+03	-0.007	0.994304
TechnicianID.dbg	1.720e+00	3.967e-01	4.336	1.45e-05 ***
TechnicianID.den	1.308e+00	5.038e-01	2.597	0.009409 **
TechnicianID.dff	1.137e+00	7.887e-01	1.442	0.149254
TechnicianID.dms	1.128e-01	7.997e-01	0.141	0.887812
TechnicianID.ecl	1.569e+00	3.581e-01	4.380	1.18e-05 ***
TechnicianID.efdj	2.227e+00	3.857e-01	5.772	7.81e-09 ***
TechnicianID.eglc	-1.652e+01	2.556e+03	-0.006	0.994843
TechnicianID.egn	1.467e+00	4.434e-01	3.308	0.000938 ***
TechnicianID.em	1.544e+00	4.044e-01	3.818	0.000135 ***
TechnicianID.emcc	-1.478e+00	6.179e-01	-2.391	0.016788 *
TechnicianID.emm	1.763e+00	3.711e-01	4.750	2.04e-06 ***

TechnicianID.emp	-4.507e-01	6.736e-01	-0.669	0.503487
TechnicianID.epdc	1.720e+00	3.865e-01	4.450	8.60e-06 ***
TechnicianID.erc	1.724e+00	3.621e-01	4.762	1.92e-06 ***
TechnicianID.esp	2.249e+00	6.877e-01	3.271	0.001072 **
TechnicianID.fcv	1.260e+00	3.901e-01	3.230	0.001236 **
TechnicianID.ffm	1.763e+00	3.848e-01	4.582	4.60e-06 ***
TechnicianID.fjcg	1.637e+00	3.348e-01	4.888	1.02e-06 ***
TechnicianID.fjdr	-1.627e+01	1.798e+03	-0.009	0.992781
TechnicianID.fjmh	-1.584e+01	5.718e+03	-0.003	0.997790
TechnicianID.fjml	-1.591e+00	1.067e+00	-1.491	0.136036
TechnicianID.fjmr	-1.775e-01	5.245e-01	-0.338	0.735050
TechnicianID.fjnr	1.677e+00	4.141e-01	4.050	5.12e-05 ***
TechnicianID.fjp	-3.216e+00	1.065e+00	-3.020	0.002527 **
TechnicianID.fjrc	8.731e-01	5.004e-01	1.745	0.081010 .
TechnicianID.fjrl	-1.536e+01	1.694e+03	-0.009	0.992766
TechnicianID.fjvm	1.160e+00	4.243e-01	2.734	0.006249 **
TechnicianID.fll	-1.683e+01	1.790e+03	-0.009	0.992498
TechnicianID.fmf	-1.662e+01	5.718e+03	-0.003	0.997681
TechnicianID.fmg	1.161e+00	3.960e-01	2.931	0.003383 **
TechnicianID.fmg2	1.242e+00	3.783e-01	3.284	0.001022 **
TechnicianID.fmr	1.528e+00	4.780e-01	3.196	0.001393 **
TechnicianID.foc	1.429e+00	4.104e-01	3.482	0.000499 ***
TechnicianID.gdlbml	1.763e+00	3.663e-01	4.813	1.49e-06 ***
TechnicianID.grd	1.962e+00	3.861e-01	5.083	3.71e-07 ***
TechnicianID.grr	1.575e+00	3.597e-01	4.377	1.20e-05 ***
TechnicianID.hce	-4.036e-01	5.383e-01	-0.750	0.453378
TechnicianID.ibr	1.180e+00	4.367e-01	2.703	0.006880 **
TechnicianID.imjr	1.781e+00	5.360e-01	3.322	0.000893 ***
TechnicianID.irg	-1.596e+01	1.344e+03	-0.012	0.990526
TechnicianID.izue	-2.486e+00	1.051e+00	-2.366	0.017998 *
TechnicianID.jabg	6.040e-01	4.542e-01	1.330	0.183572
TechnicianID.jacf	-1.616e+01	5.443e+02	-0.030	0.976312
TechnicianID.jag	7.715e-01	4.661e-01	1.655	0.097885 .
TechnicianID.jaga	9.867e-01	4.256e-01	2.319	0.020419 *
TechnicianID.jama	-4.455e-01	7.995e-01	-0.557	0.577403
TechnicianID.japr	1.234e+00	3.681e-01	3.352	0.000803 ***
TechnicianID.jarg	2.008e+00	4.017e-01	5.000	5.74e-07 ***
TechnicianID.jbc	1.485e+00	3.612e-01	4.112	3.93e-05 ***
TechnicianID.jbj	-1.715e+00	1.060e+00	-1.618	0.105719
TechnicianID.jcac	2.258e+00	3.581e-01	6.306	2.86e-10 ***
TechnicianID.jccg	-5.361e-02	7.988e-01	-0.067	0.946493
TechnicianID.jcdcp	1.447e+00	4.574e-01	3.164	0.001557 **
TechnicianID.jecc	-1.610e+01	7.665e+02	-0.021	0.983243
TechnicianID.jerj	1.150e+00	4.479e-01	2.567	0.010270 *

TechnicianID.jfcm	-6.837e-01	5.793e+03	0.000	0.999906	
TechnicianID.jfcm2	-2.188e+00	7.968e-01	-2.746	0.006035	**
TechnicianID.jgj	1.152e+00	4.448e-01	2.589	0.009612	**
TechnicianID.jjcr	1.657e+00	3.971e-01	4.173	3.00e-05	***
TechnicianID.jjpd	2.128e+00	3.621e-01	5.876	4.20e-09	***
TechnicianID.jjv	-1.635e+01	8.060e+02	-0.020	0.983812	
TechnicianID.jlac	1.488e+00	4.066e-01	3.659	0.000253	***
TechnicianID.jlg	4.361e-01	4.001e-01	1.090	0.275740	
TechnicianID.jlom	1.015e+00	4.486e-01	2.262	0.023706	*
TechnicianID.jlrr	4.110e-01	5.662e-01	0.726	0.467888	
TechnicianID.jmb	-1.703e+01	2.148e+03	-0.008	0.993674	
TechnicianID.jmbn	-1.652e+01	8.046e+02	-0.021	0.983618	
TechnicianID.jmcb	2.091e-02	5.567e-01	0.038	0.970035	
TechnicianID.jmgg	1.298e+00	3.645e-01	3.562	0.000368	***
TechnicianID.jmgr	1.831e+00	4.041e-01	4.532	5.83e-06	***
TechnicianID.jmjm	2.604e-01	6.027e-01	0.432	0.665651	
TechnicianID.jmlg	1.794e+00	5.764e-01	3.112	0.001857	**
TechnicianID.jmm	-1.643e+00	1.052e+00	-1.562	0.118216	
TechnicianID.jmrr	-9.206e-01	5.201e-01	-1.770	0.076708	.
TechnicianID.jmvg2	-1.654e+01	9.615e+02	-0.017	0.986279	
TechnicianID.jna	2.128e+00	4.350e-01	4.892	9.99e-07	***
TechnicianID.jol	-1.637e+01	9.904e+02	-0.017	0.986814	
TechnicianID.jpl	1.477e+00	3.987e-01	3.705	0.000212	***
TechnicianID.jpl2	-1.654e+01	7.942e+02	-0.021	0.983386	
TechnicianID.jrr	7.724e-01	7.984e-01	0.967	0.333333	
TechnicianID.jsl	2.480e+00	3.645e-01	6.802	1.03e-11	***
TechnicianID.jsl2	-1.566e+01	1.037e+03	-0.015	0.987947	
TechnicianID.jve	8.151e-01	5.130e-01	1.589	0.112063	
TechnicianID.ladl	3.561e+00	8.214e-01	4.335	1.45e-05	***
TechnicianID.lbe	8.754e-01	4.080e-01	2.145	0.031931	*
TechnicianID.ljc	1.856e+00	3.555e-01	5.222	1.77e-07	***
TechnicianID.lmgn	2.244e+00	3.683e-01	6.093	1.10e-09	***
TechnicianID.lod	-1.612e+01	5.639e+02	-0.029	0.977202	
TechnicianID.lrg	1.533e+00	4.025e-01	3.810	0.000139	***
TechnicianID.lrm	1.419e+00	4.057e-01	3.498	0.000469	***
TechnicianID.magm	-1.158e+00	8.013e-01	-1.445	0.148458	
TechnicianID.mall	1.972e+00	3.636e-01	5.422	5.89e-08	***
TechnicianID.mamg	-1.259e+00	8.000e-01	-1.574	0.115532	
TechnicianID.mars	3.989e-01	5.526e-01	0.722	0.470448	
TechnicianID.mavp	2.264e+00	5.351e-01	4.230	2.34e-05	***
TechnicianID.mbh	1.641e+00	3.958e-01	4.145	3.40e-05	***
TechnicianID.mca	-1.661e+01	1.338e+03	-0.012	0.990095	
TechnicianID.mcco	-1.483e+01	1.315e+03	-0.011	0.991001	
TechnicianID.mceypva	-1.761e+01	9.291e+02	-0.019	0.984881	

TechnicianID.mcf	5.128e-01	5.700e-01	0.900	0.368366
TechnicianID.mcf2	-1.645e-01	5.009e-01	-0.328	0.742569
TechnicianID.mcg	1.202e+00	3.819e-01	3.146	0.001654 **
TechnicianID.mcgg	1.367e+00	4.675e-01	2.924	0.003457 **
TechnicianID.mcl	1.406e+00	3.627e-01	3.877	0.000106 ***
TechnicianID.mcrc	1.726e+00	3.553e-01	4.858	1.18e-06 ***
TechnicianID.mdcgh	1.539e+00	3.769e-01	4.083	4.45e-05 ***
TechnicianID.mdcls	-1.720e-01	1.064e+00	-0.162	0.871623
TechnicianID.mdcmc	1.428e+00	3.669e-01	3.891	1.00e-04 ***
TechnicianID.mdcmr	2.104e+00	4.042e-01	5.204	1.95e-07 ***
TechnicianID.mdcto	6.367e-01	4.611e-01	1.381	0.167276
TechnicianID.mdg	1.677e+00	4.013e-01	4.180	2.92e-05 ***
TechnicianID.mdmmp	-1.539e+01	5.718e+03	-0.003	0.997853
TechnicianID.mdrh	1.436e-01	5.581e-01	0.257	0.796984
TechnicianID.mdrmv	1.156e+00	3.738e-01	3.092	0.001991 **
TechnicianID.mdrp	1.196e+00	3.988e-01	2.998	0.002718 **
TechnicianID.mevl	1.484e+00	3.762e-01	3.945	8.00e-05 ***
TechnicianID.mfjj	1.600e+00	4.676e-01	3.422	0.000621 ***
TechnicianID.mgcrb	1.085e+00	4.945e-01	2.194	0.028202 *
TechnicianID.mgjr	1.602e+00	4.442e-01	3.606	0.000311 ***
TechnicianID.mgm	1.331e+00	4.461e-01	2.984	0.002844 **
TechnicianID.misd	-5.034e-02	6.760e-01	-0.074	0.940641
TechnicianID.mjer	-1.609e+01	1.369e+03	-0.012	0.990619
TechnicianID.mjjm	1.980e+00	3.860e-01	5.129	2.92e-07 ***
TechnicianID.mjmv	4.167e-01	4.197e-01	0.993	0.320861
TechnicianID.mjtg	-1.575e+01	7.979e+02	-0.020	0.984248
TechnicianID.mla	1.362e+00	4.785e-01	2.847	0.004409 **
TechnicianID.mlgm	-2.496e-01	7.977e-01	-0.313	0.754401
TechnicianID.mlt	1.549e+00	4.040e-01	3.836	0.000125 ***
TechnicianID.mma	3.705e-01	4.441e-01	0.834	0.404108
TechnicianID.mmj	-1.644e+01	9.176e+02	-0.018	0.985707
TechnicianID.mmmm	3.325e-01	1.075e+00	0.309	0.757094
TechnicianID.mmo	1.388e+00	3.798e-01	3.653	0.000259 ***
TechnicianID.mmp	8.953e-01	4.803e-01	1.864	0.062305 .
TechnicianID.mms	1.494e+00	3.558e-01	4.198	2.69e-05 ***
TechnicianID.mpc	-1.655e+01	1.217e+03	-0.014	0.989144
TechnicianID.mpd	1.971e-01	5.282e-01	0.373	0.708992
TechnicianID.mpl	1.313e+00	3.772e-01	3.480	0.000501 ***
TechnicianID.mrc	1.751e+00	3.541e-01	4.946	7.59e-07 ***
TechnicianID.mre	2.104e+00	3.977e-01	5.291	1.22e-07 ***
TechnicianID.mrp	1.686e+00	5.463e-01	3.087	0.002025 **
TechnicianID.mrr	1.789e+00	4.107e-01	4.355	1.33e-05 ***
TechnicianID.mrr2	-1.630e+01	1.710e+03	-0.010	0.992395
TechnicianID.msldc	1.208e+00	5.266e-01	2.294	0.021791 *

TechnicianID.msm	-1.596e+01	1.300e+03	-0.012	0.990206
TechnicianID.mvf	-5.044e-01	7.910e-01	-0.638	0.523660
TechnicianID.nbp	1.319e+00	4.057e-01	3.252	0.001145 **
TechnicianID.nbs	1.247e+00	5.085e-01	2.452	0.014225 *
TechnicianID.ngs	-1.490e+01	1.494e+03	-0.010	0.992040
TechnicianID.nrfj	1.617e+00	3.427e-01	4.719	2.37e-06 ***
TechnicianID.obm	1.415e+00	4.796e-01	2.950	0.003181 **
TechnicianID.oyma	5.343e-01	4.087e-01	1.307	0.191148
TechnicianID.pagn	1.554e+00	5.579e-01	2.785	0.005346 **
TechnicianID.pbe	-1.674e+01	2.333e+03	-0.007	0.994273
TechnicianID.pcmo	-1.621e+01	6.261e+02	-0.026	0.979342
TechnicianID.pcv	-1.633e+01	9.036e+02	-0.018	0.985581
TechnicianID.pdpmda	-1.620e+01	5.649e+02	-0.029	0.977119
TechnicianID.pjrc	2.088e+00	5.241e-01	3.984	6.78e-05 ***
TechnicianID.pla	1.329e+00	7.928e-01	1.676	0.093670 .
TechnicianID.plg	1.601e+00	4.299e-01	3.723	0.000197 ***
TechnicianID.plja	9.240e-01	3.855e-01	2.397	0.016540 *
TechnicianID.pmc	8.559e-01	5.392e-01	1.587	0.112426
TechnicianID.pmc2	1.410e+00	4.037e-01	3.493	0.000478 ***
TechnicianID.pmm	-1.673e+01	2.858e+03	-0.006	0.995329
TechnicianID.pmrc	1.603e+00	3.733e-01	4.293	1.76e-05 ***
TechnicianID.ppf	1.419e+00	3.751e-01	3.783	0.000155 ***
TechnicianID.ppnv	2.123e+00	4.242e-01	5.004	5.62e-07 ***
TechnicianID.ppp	1.832e+00	6.932e-01	2.643	0.008222 **
TechnicianID.prn	1.842e+00	3.593e-01	5.128	2.93e-07 ***
TechnicianID.psmt	-7.199e-01	5.259e-01	-1.369	0.171043
TechnicianID.radc	-1.666e+01	3.299e+03	-0.005	0.995972
TechnicianID.rbl	1.562e+00	3.659e-01	4.269	1.96e-05 ***
TechnicianID.rcfg	1.801e+00	4.290e-01	4.197	2.70e-05 ***
TechnicianID.rcg	-1.615e+01	1.573e+03	-0.010	0.991805
TechnicianID.rev	-1.660e+01	1.520e+03	-0.011	0.991290
TechnicianID.rga	1.033e+00	3.796e-01	2.721	0.006511 **
TechnicianID.rgr	1.427e+00	3.631e-01	3.930	8.51e-05 ***
TechnicianID.rljm	1.265e+00	6.969e-01	1.815	0.069479 .
TechnicianID.rlm	1.092e+00	3.889e-01	2.808	0.004987 **
TechnicianID.rmm	2.087e+00	4.027e-01	5.182	2.20e-07 ***
TechnicianID.rmt	-2.058e+00	1.049e+00	-1.963	0.049637 *
TechnicianID.rop	-1.665e+01	1.642e+03	-0.010	0.991910
TechnicianID.rro	2.069e+00	3.545e-01	5.837	5.30e-09 ***
TechnicianID.rrr	5.335e-01	4.280e-01	1.246	0.212621
TechnicianID.ryf	-1.559e+01	5.718e+03	-0.003	0.997824
TechnicianID.shm	1.288e+00	7.895e-01	1.632	0.102733
TechnicianID.sjs	7.498e-01	5.422e-01	1.383	0.166646
TechnicianID.slf	-1.328e+00	1.108e+00	-1.199	0.230703

TechnicianID.slr	1.690e+00	3.845e-01	4.396	1.10e-05 ***
TechnicianID.sma	7.377e-01	5.086e-01	1.450	0.146940
TechnicianID.smc	1.754e+00	3.951e-01	4.440	9.00e-06 ***
TechnicianID.spaj	-4.919e-01	6.218e-01	-0.791	0.428883
TechnicianID.spl	-1.692e+01	1.101e+03	-0.015	0.987733
TechnicianID.suj	5.709e-01	6.191e-01	0.922	0.356495
TechnicianID.tig	-1.665e+01	1.449e+03	-0.011	0.990829
TechnicianID.tlq	1.225e+00	5.993e-01	2.044	0.040913 *
TechnicianID.tmf	1.617e+00	3.706e-01	4.363	1.29e-05 ***
TechnicianID.vsa	1.276e+00	3.739e-01	3.414	0.000641 ***

Signif. codes: 0 ‘***’ 0.001 ‘**’ 0.01 ‘*’ 0.05 ‘.’ 0.1 ‘ ’ 1

Approximate significance of smooth terms:

	edf	Ref.df	Chi.sq	p-value
s(FieldSize)	1.000e+00	1.000	0.212	0.644939
s(Year)	1.096e+01	12.000	79.106	< 2e-16 ***
s(FieldID)	3.536e-04	2749.000	0.000	1.000000
s(xutm,yutm):Year2006	4.177e+00	5.392	5.392	0.411426
s(xutm,yutm):Year2007	7.721e+00	8.582	49.404	< 2e-16 ***
s(xutm,yutm):Year2008	7.214e+00	8.284	21.836	0.003960 **
s(xutm,yutm):Year2009	2.000e+00	2.000	3.472	0.176261
s(xutm,yutm):Year2010	2.760e+00	3.344	1.671	0.643687
s(xutm,yutm):Year2011	2.000e+00	2.000	1.627	0.443415
s(xutm,yutm):Year2012	5.319e+00	6.677	27.275	0.000267 ***
s(xutm,yutm):Year2013	3.984e+00	5.107	21.640	0.000673 ***
s(xutm,yutm):Year2014	3.042e+00	3.803	2.835	0.591291
s(xutm,yutm):Year2015	2.124e+00	2.240	9.172	0.013749 *
s(xutm,yutm):Year2016	2.000e+00	2.000	10.610	0.004969 **
s(xutm,yutm):Year2017	2.000e+00	2.000	12.757	0.001698 **
s(xutm,yutm):Year2018	5.730e+00	7.111	17.646	0.014492 *

Signif. codes: 0 ‘***’ 0.001 ‘**’ 0.01 ‘*’ 0.05 ‘.’ 0.1 ‘ ’ 1

R-sq.(adj) = 0.455   Deviance explained =   50%
fREML = 10405 Scale est. = 1   n = 9340

SI References

1. B. N. Cass, L. M. Hack, E. E. Grafton-Cardwell, J. A. Rosenheim, Impacts of fruit-feeding arthropod pests on oranges and mandarins in California. *J. Econ. Entomol.* **112**, 2268-2277 (2019).
2. B. N. Cass, L. M. Hack, T. Mueller, D. Buckman, E. E. Grafton-Cardwell, J. A. Rosenheim, Arthropod infestation levels on mandarins in California. *J. Econ. Entomol.* **113**, 2335-2342 (2020).
3. G. Livingston, L. Hack, K. P. Steinmann, E. E. Grafton-Cardwell, J. A. Rosenheim, An ecoinformatics approach to field-scale evaluation of insecticide effects in California citrus: are citrus thrips and citrus red mite induced pests? *J. Econ. Entomol.* **111**, 1290-1297 (2018).
4. M. H. Meisner., T. Zaviezo, J. A. Rosenheim, Landscape effects on *Lygus hesperus* densities, cotton yield, and pesticide use. *Pest Manag. Sci.* **73**, 232-239 (2017).
5. S. Parsa, R. Ccanto, J. A. Rosenheim, Resource concentration dilutes a key pest in indigenous potato agriculture**.** *Ecol. Appl.* **21**, 539-546 (2011).
6. J. A. Rosenheim, M. H. Meisner, Ecoinformatics can reveal yield gaps associated with crop-pest interactions: a proof-of-concept. *PLoS ONE* **8(**11), e80518 (2013).
7. D. Paredes, J. A. Rosenheim, R. Chaplin-Kramer, S. Winter, D. S. Karp, Landscape simplification increases vineyard pest outbreaks and insecticide use. *Ecol. Lett.* **24**, 73-83 (2021).
8. D. Paredes, J. A. Rosenheim, D. S. Karp, Causes and consequences of pest population stability in agricultural landscapes. *Ecol. Appl.* (*in press*) (2022).
